# Understanding visual perception in visual snow syndrome: a battery of psychophysical tests plus the 30-day clinical diary

**DOI:** 10.1093/braincomms/fcae341

**Published:** 2024-09-30

**Authors:** Simona Garobbio, Reza Mazloum, Michael Rosio, Jeanette Popovova, Raphaela Schöpfer, Fabienne C Fierz, Leah R Disse, Konrad Peter Weber, Christoph J Schankin, Lars Michels, Michael H Herzog

**Affiliations:** Laboratory of Psychophysics, Brain Mind Institute, École Polytechnique Fédérale de Lausanne (EPFL), 1015 Lausanne, Switzerland; Department of Neuroradiology, University Hospital Zurich, 8091 Zurich, Switzerland; Department of Neuroradiology, University Hospital Zurich, 8091 Zurich, Switzerland; Department of Neuroradiology, University Hospital Zurich, 8091 Zurich, Switzerland; Department of Neuroradiology, University Hospital Zurich, 8091 Zurich, Switzerland; Department of Ophthalmology, University Hospital Zurich, 8091 Zurich, Switzerland; Department of Ophthalmology, University Hospital Zurich, 8091 Zurich, Switzerland; Department of Neurology, University Hospital Zurich, 8091 Zurich, Switzerland; Department of Ophthalmology, University Hospital Zurich, 8091 Zurich, Switzerland; Department of Neurology, University Hospital Zurich, 8091 Zurich, Switzerland; Department of Neurology, Inselspital, Bern University Hospital, University of Bern, 3010 Bern, Switzerland; Department of Neuroradiology, University Hospital Zurich, 8091 Zurich, Switzerland; Clinical Neuroscience Center, University Hospital Zurich, 8091 Zurich, Switzerland; Laboratory of Psychophysics, Brain Mind Institute, École Polytechnique Fédérale de Lausanne (EPFL), 1015 Lausanne, Switzerland

**Keywords:** visual snow syndrome, psychophysics, 30-day clinical diary, objective diagnosis, individual differences

## Abstract

Patients with visual snow syndrome (VSS) experience uncountable flickering tiny dots in the entire visual field. Symptoms often persist over the years. Very little is known about altered perception in VSS. VSS is diagnosed based on subjective reports because there is no manual with objective measures. In this study, 20 patients with VSS and 17 healthy controls performed a battery of tests assessing visual acuity, contrast sensitivity, illusion perception, spatial–temporal vision, motion perception, visual attention, and selective attention. Surprisingly, except for one test, which is the honeycomb illusion, patients performed at the same level as controls. Patients reporting black and white visual snow performed better in the Stroop test compared to patients reporting other visual snow colours. In addition to a clinical visit, the 30-day clinical diary was administered to patients to broadly measure their symptom severity. We found that better performance in the tests, in particular in the contrast and coherent motion tests, was correlated with lower VSS symptoms, weaker VS characteristics (e.g. density and size) and lower VS severity. Our results suggest that, even if visual abilities are not deteriorated by VSS, they can determine how severe symptoms are, and show that VSS is an heterogenous disorder where symptoms and visual abilities vary between patients, for instance depending on the VS colour. The study was primarily designed to identify tests where performance differs between controls and patients. In addition, exploratory analyses were conducted to initiate an understanding of the overall pattern of relationships between patients’ visual abilities and symptoms, which is of clinical relevance. Future studies with more power are necessary to validate our findings.

## Teaser

Garobbio *et al*. reported that patients with visual snow syndrome are generally not impaired in psychophysical tests. However, better test performances are correlated with lower severity of patient’s symptoms. The findings suggest that measures of visual perception could be used in the future to better describe patients with visual snow syndrome.

## Graphical Abstract

**Graphical abstract:**
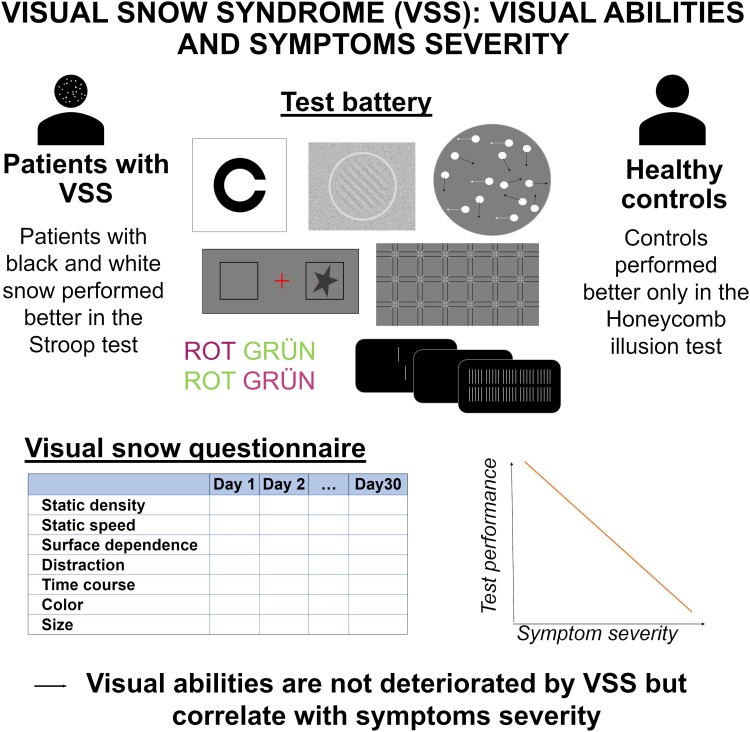

## Introduction

**Figure 1:**
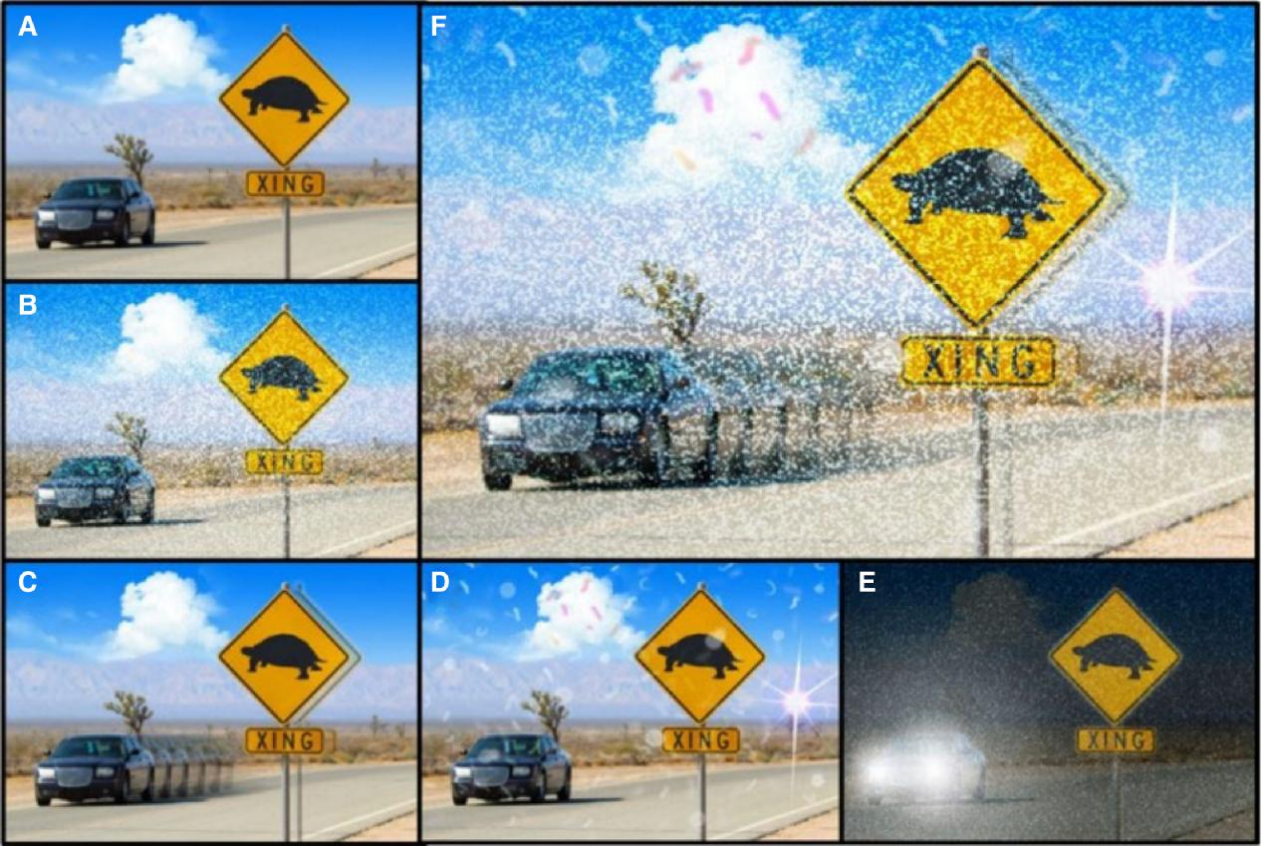
**Symptoms of VSS.** (**A**) Normal vision. (**B**) Visual snow. (**C**) Palinopsia (visual trailing behind the car/afterimage next to the sign). (**D**) Enhanced entoptic phenomena: floaters, blue field entopic phenomena, photopsia and coloured blobs. (**E**) Nyctalopia (impaired night vision). (**F**) Visual snow. Composite illustration with possible combination of symptoms (n.b., photophobia–sensitivity to light–is another VSS symptom not represented here). Panel **A** used under standard Shutterstock license. Panels **B–F** from: Metzler AI & Robertson CE. Metzler *et al.*^73^; used with permission of Mayo Foundation for Medical Education and Research, all rights reserved.

Visual snow (VS) is a visual disturbance with otherwise normal ophthalmic findings.^1^ The disease is characterized by perceiving innumerable flickering dots superimposed on the entire visual field, similar to analogue TV noise.^2^ When associated with additional visual symptoms, we talk about the ‘visual snow syndrome’ (VSS^2,3^). The additional symptoms belong to the following four categories: palinopsia, enhanced entoptic phenomena, photophobia and nyctalopia (see Fig. 1). Most patients are severely affected in their daily life, and VSS is frequently associated with migraine, tinnitus, impaired concentration, lethargy, anxiety, depression, balance disorder and tremor.^4-7^ VSS is estimated to affect ∼2.2% of the population,^8^ and it is innate in 40% of cases; otherwise, it usually starts in young adulthood.^9^ Symptoms are stable over time.^10,11^ The pathophysiology of VSS remains largely elusive. Only 3–7% of VSS patients report a family history of VSS, but 30% to 60% report migraine occurring in their relatives.^12^ To date, there are no established treatments for VSS. Pharmacological and nonpharmacological options have been suggested, but only a small part of the patient population benefit from them.^7,13-16^

VSS is currently diagnosed based entirely on subjective reports since there are no available objective tests.^17^ Recently, Puledda *et al*.^10^ developed a questionnaire, the ‘30-day clinical diary’, where patients report their symptoms on a daily basis for 30 days, allowing to clinically evaluate treatment response and symptom progression. Also, the underlying pathophysiology remains elusive.^13,18,19^ Few studies used visual tests to study visual processing in VSS. McKendrick *et al*.^20^ found altered contrast (i.e. a difference in the subjective contrast appearance, but not poorer performance) and impaired luminance perception in the patients compared to controls, while performances in a global form test and in a global dot motion test did not differ between the two groups. In a follow-up experiment with a larger patient sample size (*n* = 35 versus *n* = 16 in the 2017 study), the contrast perception alteration finding was confirmed, while patients’ performance on luminance perception did not differ from the one of controls.^21^ Eren *et al*.^22^ reported an impaired dynamic contrast sensitivity in VSS patients compared to controls for temporal modulation frequencies of 15 Hz, but not for the other frequencies tested (i.e. 20, 25, 30, 35 and 40 Hz). Yoo *et al*.,^23^ using another variant of the contrast sensitivity test (i.e. the Functional Acuity Contrast Test^24^), reported that contrast sensitivity was decreased only in two out of seven patients. Oculomotor behaviour of patients has also been studied, suggesting that saccadic behaviour could provide an objective measure to evaluate VSS. Indeed, compared to controls, patients showed shortened prosaccade latencies, more erroneous saccades towards non-target locations^12,25^ and delayed onset of inhibition of return.^26^

In the current study, we aimed to investigate the facets of VSS with a battery of seven psychophysical tests: visual acuity, contrast sensitivity, Honeycomb white illusion, visual backward masking, coherent motion, Posner and Stroop. We hypothesized that patients would perform worse than controls in contrast sensitivity and attention tests, as these tests are presumed to evaluate the neuronal processing stages thought to be involved in VSS. Indeed, increased activity in the lingual gyrus has been shown in VSS patients which should reflect in the Stroop test;^27,28^ the attention allocation deficits may be captured by the Posner test;^29,30^ and an altered contrast perception would be in line with the hypothesis of elevated excitability in the primary visual cortex of VSS patients.^20,21^ In the contrast test, we introduced noisy dots with the intention to render the test more difficult for patients. As exploratory tests, we included the visual backward masking test, which we have previously shown to be a highly sensitive test targeting spatio-temporal visual processing;^31,32^ the motion direction sensitivity test to also target the extra-striate cortex,^33^ whose role in VS is not known yet;^34^ and the Honeycomb white illusion, as we hypothesized a possible interaction between the barbs and the snow.^35^ The visual acuity test served as a control test. We included patients with all colours of VS, not only with black or white snow percepts, as it is usually done.

Within the patient group, we then explored whether test performances correlated with the severity of symptoms. To the best of our knowledge, this was the first attempt to investigate whether a relationship between test performances and VSS symptoms exist.

## Materials and methods

### Participants and general procedure

Twenty patients with VSS and 17 controls participated in the study. Diagnosis of VSS was made by a licensed neurologist or ophthalmologist based on the ICHD-3 criteria.^36^ Included controls had no history of migraine or tinnitus, as assessed through the HARDSHIP score.^37^ The following standard tests were performed, and normal outcome was required for inclusion: visual acuity test, slit lamp, visual field test and optical coherence tomography (OCT). Exclusion criteria included the use of psychopharmacological drugs and/or recreational drugs within 6 months prior to the study, as well as the presence of any degenerative disorders of the central nervous system or major psychiatric disorder as assessed through history-taking by the study physician. A description of groups’ demographics is provided in Table 1.

**Table 1:** Description of group demographics for VSS patients (VSS) and controls (ctrl)

	VSS	ctrl
*N*	20	17
Gender (F/M)	7/13	12/5
Age	31.6 ± 6.55	26.0 ± 5.34
Education (*n*)		
High school	3	10
Bachelor	7	5
Master’s	9	2
Ph.D.	1	0
Handedness (L/R)	4/16	0/17
Migraine (%)^a^	45%	0
Migraine with aura (%)^b^	77%	-
Tinnitus (%)^c^	80%	0

^a^Migraine was determined by HARDSHIP score.

^b^Migraine with aura was assessed only in patients with migraine.

^c^Tinnitus within the last 12 months.

The study complied with the Declaration of Helsinki and was pre-registered (clinicaltrials.gov, ID: NCT04902365) and approved by the Ethics Committee of the Canton Zurich in Switzerland (approval number: 2021-01128). All participants gave written informed consent prior to the experiment, were informed that they could withdraw from the experiment at any time and were reimbursed for their participation.

Patients underwent a clinical visit, in which their VSS symptoms were recorded. Patients were also instructed to fill in the 30-day clinical diary.^10^ The experimental session took place at the Psychiatric University Hospital in Zurich (Switzerland) on a single day. It lasted 1 h for controls and 2 h for patients. The experimental session included a battery of psychophysical tests for both controls and patients. It should be noted that these patients were tested longitudinally in a separate functional MRI neurofeedback study, which will be published as a standalone study after completion.

### Tests and extracted variables

#### VSS symptoms

The symptoms experienced by the patients were recorded during a clinical visit and through the 30-day clinical diary developed by Puledda *et al*.^10^ and are reported in Table 2.

**Table 2:** Description of symptoms experienced by the patients. Upper section: symptoms recorded during the clinical visit; lower section: symptoms assessed through the 30-day questionnaire

	M ± SD	VSS symptoms	M ± SD, %
VSS year duration	6.53 ± 8.42	VS	4.64 ± 1.52, 100%
		Palinopsia	2.92 ± 2.62, 75%
		Blue field entoptic phenomena	4.03 ± 2.94, 90%
		Spontaneous photopsia	2.75 ± 2.98, 70%
		Floaters	5.13 ± 2.68, 95%
		Flashes when eyes closed in darkness	3.93 ± 2.56, 90%
		Photophobia	4.40 ± 3.26, 75%
		Nyctalopia^a^	2.66 ± 2.44, 68%

VS characteristics	M ± SD	Colour	VS severity^c^	M ± SD
Density	2.73 ± 0.90	Black and white: *n* = 8	Outdoor: sunny day	5.11 ± 1.78
Speed	3.07 ± 0.78	Transparent: *n* = 6	Outdoor: cloudy day	3.68 ± 1.85
Surface dependence	2.90 ± 0.78	Flashing: *n* = 2	Outdoor: rainy day	4.22 ± 1.51
Distraction	2.66 ± 0.66	Coloured: *n* = 0	Indoor	3.56 ± 1.46
Time course	2.81 ± 1.06	Other^b^: *n* = 4	Fluorescent lighting	2.56 ± 1.41
Size	2.38 ± 1.19		Outdoor: night-time	2.50 ± 1.90

M ± SD: Group mean and standard deviation; %: percentage of patients suffering by the respective symptom.

^a^One datum is missing for nyctalopia.

^b^Other colours were the following: one patient reported either 1, 3 or 4 depending on the day; one patient reported 5 every day; one patient reported 1 or 3 depending on the day, and another one reported 2/3/4 every day.

^c^Two patients did not provide these data, and two more data are missing for fluorescent lighting and outdoor: night time.

During the clinical visit, patients were asked to score each individual symptom of their VSS symptoms on a subjective rating scale from 0 to 10 (0 = no symptoms at all, 10 = most severe imaginable symptom): visual snow, palinopsia, blue field entoptic phenomena, spontaneous photopsia, floaters, flashes when eyes closed in darkness, photophobia and nyctalopia. VSS year duration was also recorded.

The 30-day clinical diary questionnaire developed by Puledda *et al*.^10^ included two parts. First, over the course of 30 days prior to doing the psychophysical test battery, patients scored seven items daily aiming at describing different characteristics of their VS. Higher numbers represent higher clinical severity, except in the case of colour where scores represent different clinical categories. The seven VS characteristics were the following: density (range 0–6), speed (range 0–4), surface dependence (i.e. visibility on different surfaces; range 0–4), distraction (i.e. levels of distractions caused by the snow; range 0–4), time course (i.e. variation of VSS during 24 h; range 0–4), colour (1: black and white, 2: transparent, 3: flashing, 4: coloured, 5: other) and size (range 0–5). Since VS characteristics are highly stable over time,^10^ we calculated the mean value for each. Second, patients were asked to score their VS severity on a scale from 1 to 7, with 1 being the worst condition and 7 the best, to the following six environmental light conditions: outdoor sunny day, outdoor cloudy day, outdoor rainy day, indoor, fluorescent lighting and outdoor night-time. The six scores were recorded and included in the analysis.

#### Battery of psychophysical tests

The following tests were included: Freiburg visual acuity, contrast sensitivity with noisy background, motion direction sensitivity, visual backward masking, Honeycomb white illusion, Stroop and Posner tests. The tests were presented to participants in the same order and were repeated twice.

The stimuli were displayed on an ASUS VG27AQ1A monitor (59.6 × 33.5 cm, 2560 × 1440 pixels, 170 Hz). Participants sat in a dimly illuminated room and, when applicable, were asked to wear their glasses. Participants sat 65 cm away from the screen for the Honeycomb white illusion, Posner and motion direction sensitivity tests, while for all other tests, the distance to the screen was 2 m. An auditory feedback tone was provided after incorrect responses in all tests, except for the Honeycomb white illusion. Feedback was provided to ensure higher response consistency through faster learning, better motivation and less apparent motor bias (i.e. adjust the subjective zero to the objective one). Practice trials (one for Honeycomb white illusion, eight for Freiburg visual acuity and contrast sensitivity, 10 for Stroop and Posner and 28 for motion direction sensitivity and visual backward masking) were provided to ensure proper understanding of the tests.

For tests using psychometric functions (Freiburg visual acuity, motion direction sensitivity, contrast sensitivity and visual backward masking), test levels were determined using the QUEST adaptive procedure^38^ on a logarithmic scale, aiming for a 75% correct response rate, except for Freiburg visual acuity where the threshold corresponds to 62.5% as a four-alternative forced-choice test. The thresholds were estimated using maximum likelihood analysis, taking all trials into account. The guessing rate was set to 50%, but 25% for Freiburg visual acuity, and the rate of motor errors was set to 3%. Blocks were discarded when the fit was invalid (i.e. when the point of subjective equality was outside the search space or the process did not converge), which led to a total of 2.4% of values discarded.

The stimulus programs were implemented in MATLAB (Version R2020b, The Mathworks Inc.) using the Psychophysics Toolbox^39^ and run on GNU Octave (Version 6.2.0).

A description of each test is given below (unless stated otherwise, responses were provided using hand-held push buttons).

##### Freiburg visual acuity (VA)

**Figure 2:**
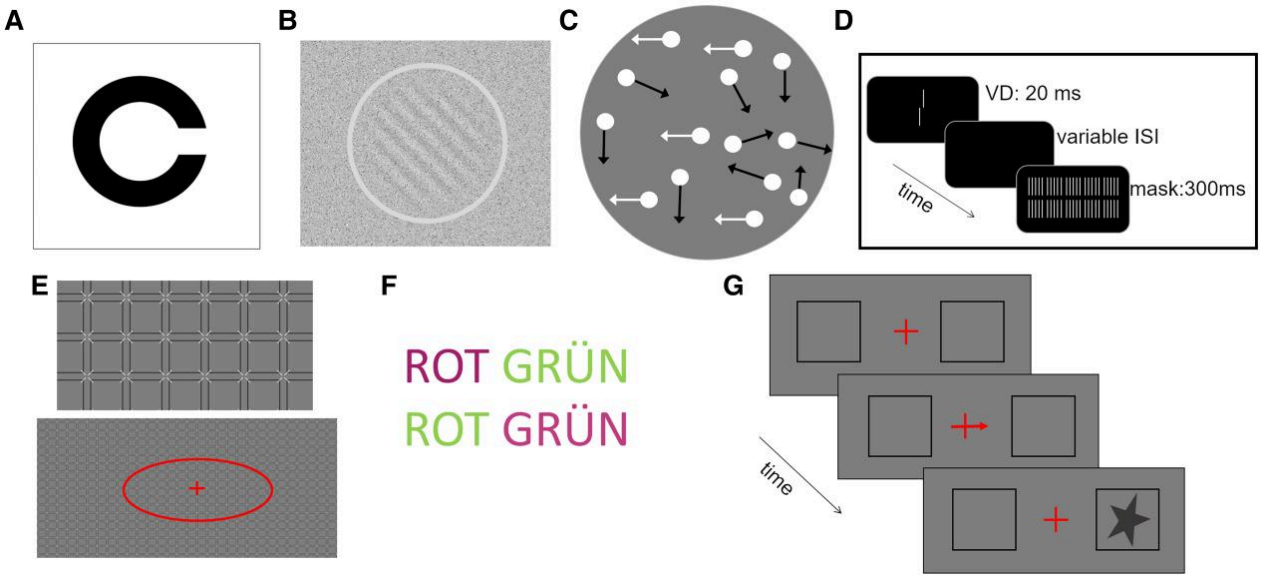
**Illustrations of the tests used.** (**A**) Freiburg visual acuity. (**B**) Contrast sensitivity. (**C**) Motion direction sensitivity (only a few dots are shown, where arrows are used to depict the motion directions but were not presented in the real test). (**D**) Visual backward masking (VD, vernier duration; ISI, inter-stimulus interval). (**E**) Honeycomb white illusion (upper panel shows a zoomed detail of the stimuli). (**F**) Stroop test, congruent (upper line) and incongruent (lower line) conditions (ROT, red; GRÜN, green). Magenta is used to represent red to make the figure colour-blind friendly. (**G**) Posner test (congruent trial).

Binocular visual acuity was measured using the Freiburg visual acuity test.^40^ Landolt-C black optotypes with randomized gap orientations were presented on a white background with 100 cd/m^2^ luminance (Fig. 2A). Participants indicated the direction of the gap (‘up’, ‘down’, ‘left’ or ‘right’) by pressing the corresponding button on a keypad. The optotypes were presented until participant’s reply. The size of each optotype changed adaptively over 40 trials. The test provided the decimal visual acuity value.

##### Contrast sensitivity (con)

Participants indicated, in a series of 60 trials, whether a sine grating was oriented clockwise or counterclockwise in respect to the vertical axis of the screen. The grating had a mean luminance of 50 cd/m^2^, an orientation of 45°, a spatial frequency of 3 cycles per arcdeg, a diameter of 3 arcdeg and a 0.7 arcdeg wide edge with a cosine-shaped transition from full contrast to zero. An annulus around the grating (luminance: 70 cd/m^2^) indicated when the grating was potentially presented. Both the grating and the annulus were displayed for 200 ms. The sine grating and background were overlaid with stationary and opaque noise (relative area covered by noise: 20%, noise contrast: 80%, grain size of noise pixels: 60 arcsec), similar to the one used in Nishina *et al*.^41^ (Fig. 2B). The noise pattern stayed on throughout the experiment, but was changed with the onset and offset of the sine grating patch to mask transient signal components. The measure of interest was the contrast threshold of the grating.

##### Motion direction sensitivity (CMot)

The test was based on the global dot motion stimulus used by McKendrick *et al*.^20^ Participants were required to discriminate between a leftward and a rightward motion in a random dot pattern, where a certain ratio of dots moved coherently in either the left or right direction, while the remaining dots moved in random directions. A total of 100 dots (dot size: 8.6 arcmin) moved at 2.86 arcdeg/s within a circular area (diameter: 10 arcdeg) for 400 ms (Fig. 2C). The stimulus differed from the one used by McKendrick *et al*.^20^ in three ways: feedback was provided upon incorrect responses to be consistent with the other tests; the stimulus was presented at higher contrast to ensure that motion detection and not contrast sensitivity abilities are tested (dot luminance was 100 cd/m^2^; background luminance was 10 cd/m^2^ versus 90 cd/m^2^ for the dots and 50 cd/m^2^ for the background in McKendrick *et al*.); and the randomly moving dots could move in any direction, including those within ±10° of the coherent motion direction, to maintain dot density homogeneity and prevent observers from deducing the correct answer from incoherently moving dots. The measure of interest was the ratio of coherently moving dots, which was determined based on 60 trials.

##### Visual backward masking (VBM)

First, to familiarize participants with the target, a vernier stimulus was presented for 18 ms for 20 trials. The white vernier stimulus consisted of two vertical bars slightly offset in the horizontal direction and was displayed on a dark background (2 cd/m^2^). During the 20 trials, participants indicated the offset direction of the lower bar in respect to the upper bar (left or right). Secondly, the vernier stimulus (duration: 18 ms, offset: 75 arcsec) was followed by the adaptively controlled variable inter-stimulus interval (ISI; i.e. a black screen) and a grating mask for 300 ms.^32,42,43^ The grating mask consisted of 25 aligned elements, each of the same length as the vernier stimulus (Fig. 2D). Participants were instructed to report the offset direction of the lower bar of the vernier stimulus for a total of 60 trials.

##### Honeycomb white illusion (HoneyW)

This paradigm was based on a previous study by Bertamini *et al*.^35,44^ The Honeycomb white illusion is characterized by the inability to perceive white barbs in the periphery of a uniform texture. The texture filled the screen and consisted of dark grey squares (length: 1.46 arcdeg, width: 0.05 arcdeg, distance between squares: 0.27 arcdeg) and white barbs (length: 0.31 arcdeg, width: 0.04 arcdeg; anti-aliasing was applied). Participants fixated a red central cross and adjusted the size of a red ellipse on the x and y axis using the computer mouse so that all barbs within the ellipse became perceptible to them (Fig. 2E). The red ellipse was displayed with a random size within the screen size at the beginning of each trial. No time limit was set for the adjustment. The area of the adjusted ellipse was recorded. Between the two performed trials, random black and white checkerboards (30 random masks presented for 0.5 s each and made of squares of 0.54 arcdeg in side) were presented to eliminate after-image effects.

##### Stroop

Participants were instructed to report the colour of a word as quickly as possible while ignoring the word’s meaning.^45,46^ For example, if the word ‘green’ was written in red, participants had to report ‘red’. The participant’s mother-tongue words for red and green (e.g. German: ‘rot’ and ‘grün’ or French: ‘rouge’ and ‘vert’) were displayed in four conditions: word ‘green’ written in red or green, and word ‘red’ written in red or green (text was presented with maximum luminance, on a 10 cd/m^2^ background; Fig. 2F). In congruent trials, the colour and meaning of the word matched (i.e. ‘red’ written in red or ‘green’ written in green), while in incongruent trials, they did not match (i.e. ‘red’ written in green and ‘green’ written in red). Participants completed 15 trials for each condition in random order, and no condition was presented more than four times consecutively. After removing incorrectly answered trials, outlier trials were removed based on a modified z-score with a 3.5 standard deviation (*SD*) criterion after log-transforming the data (on average, one trial per participant was removed). The Stroop effect was defined as the difference in median reaction time between correctly answered incongruent and congruent trials divided by the average response time.

##### Posner

The test was based on the implementation used by Feldmann-Wüstefeld and Schubö^47^ of the cueing task devised by Posner.^48^ A central red cross (size: 0.6 arcdeg, line width: 4 arcmin) and two black squares (each at a 12° eccentricity, one to the left and the other to the right of the cross, with a side length of 4°) were presented during the entire trial on a background luminance of 10 cd/m^2^. In each trial, after 750 ms of central fixation, the horizontal line of the cross was replaced for 500 ms by an arrow (width: 14 arcmin) that served as an endogenous cue, indicating the potential location of a star stimulus that would appear in either the left or right square. Note that we changed the cue duration from the 1000 ms used by Feldmann-Wüstefeld and Schubö (2013) to 500 ms in order to shorten the test duration. Then, the star appeared within an ISI of 100 to 600 ms within one of the squares (star luminance: 1 cd/m^2^; Fig. 2G). Participants were instructed to maintain fixation on the central red cross and promptly indicate the square where the star appeared (left or right). The next trial started within an inter-trial-interval (ITI) of 500 to 1000 ms. The arrow cue predicted the star’s location correctly in 70% of the trials (congruent trials) and incorrectly in the rest of the trials (incongruent trials). Participants completed 80 trials. After removing incorrectly answered trials, outlier trials were removed based on modified z-score with a 3.5 *SD* criterion after log-transforming the data (on average, 0.8 trial per participant was removed). The Posner effect was defined as the difference in median reaction time between correctly answered incongruent and congruent trials divided by the average response time.

### Test–retest of psychophysical tests

Each test was repeated twice. To remove test results (of both repetitions) of participants who displayed exceptional performance instability or stability across the two repetitions, we calculated the absolute score differences (i.e. the absolute value of the score at test repetition one minus the score at test repetition two) and identified outliers according to a 3.5 *SD* criterion.^49^ Fourteen test results out of 259 (37 participants * seven tests) were excluded from the dataset (Supplementary Table 1).

Before averaging the valid scores of the two repetitions to obtain one score for each participant for each test, we assessed test–retest reliability by computing two-way mixed effects models (i.e. intraclass correlation of type (3,1) or ICC31^50,51^) and by visually inspecting the participant’s score across the two test repetitions using scatter plots. For the Posner and Stroop tests, reliability was assessed both for the difference scores and for the median reaction times. Reaction times of the incongruent and congruent conditions (StroopIncRT, StroopConRT, PosnerIncRT, PosnerConRT) were also included because the difference scores may have misleadingly low test–retest reliability due to reduced between-participants variance as predicted by the reliability paradox.^52,53^

Test–retest reliability assessed with intra-class correlations coefficients (ICCs) and scatter plots suggested that all tests except Stroop showed reliable test–retest (Supplementary Table 2 and Supplementary Fig. 1).

From now on, the average scores from the two test repetitions were used.

### Preprocessing of test scores

The preprocessing steps had three objectives: firstly, to transform the test scores to better approximate normal distributions; secondly, to remove outliers; and lastly, to standardize the data for seamless comparisons between various tests. By making the scores more normally distributed, we avoid asymmetrical outlier removal and reduce bias in the *z*-scores that would otherwise impact the comparability across tests. To obtain more accurate estimates of the standard deviations, the data of the two groups were merged for the preprocessing steps.

Before preprocessing, all score distributions, except for Posner, violated the normality assumption as assessed by the Shapiro–Wilk test, mainly due to skewness (Supplementary Fig. 2 and Supplementary Table 3). The preprocessing was done as follows: first, we computed modified *z*-scores (based on the median and median absolute deviation) and removed outliers according to a 3.5 *SD* criterion;^49^ second, we used the *Yeo–Johnson* power transformation (using the PowerTransformer function from sklearn.preprocessing Python package^54^) and optimized its λ exponent to maximize normality according to the Shapiro–Wilk test; and third, we included the previously removed outliers and transformed the variables using the *Yeo–Johnson* transformation with the optimized λ parameter. We repeated the outlier removal step as in Step 1 on the transformed data. After removing the outliers, the dataset had 5.4% missing scores across all tests and all subjects (Supplementary Table 4). Participants with missing scores were not excluded, and no data imputation was performed. Instead, pairwise deletion was applied for computing correlations and *t*-tests.

Lastly, we reversed the sign of all variables, except VA and HoneyW scores, to make higher scores correspond to better performance (see Supplementary Fig. 3 for the resulting scores distributions and Supplementary Table 3 for the pre-processing parameters).

### Data analysis

#### Performances in psychophysical tests

First, we tested whether performance in the tests differed between patients and controls. We computed two-tailed Student's *t*-tests and corrected for multiple comparisons using the Bonferroni–Holm correction.^55^ Cohen's *d* effect sizes were also used to interpret the results. We used Cohen's guidelines,^56^ which considered effect sizes from *t*-tests of 0.2, 0.5 and 0.8 as small, medium and large in magnitude, respectively.

Second, we tested whether patients’ co-morbidities (i.e. tinnitus and migraine) influenced test performances by comparing the performance of all tests between patients who had a specific symptom and those who did not with two-tailed Welch’s *t*-tests.

Third, we investigated whether VS colour influenced test performances. To do so, we separated the patients into two groups depending on whether they reported black and white snow (*N* = 8) or any other colour categories (*N* = 12) and performed a three-way Welch’s ANOVA (groups: controls, black and white VS, non-black and white VS) for each test. When appropriate, post-doc Welch's *t*-tests were performed to investigate the group effect, and *P*-values were corrected with the Bonferroni–Holm method for multiple comparisons.^55^ Please note that all patients reporting something else than black and white snow were grouped together because of the small sample size.

#### Tests performance versus symptoms (within patients)

We tested for potential associations between performance of the psychophysical tests and clinical symptoms by computing Spearman correlations. The correlations between test performances and VSS year duration were corrected for age by computing partial correlation. Since this was an exploratory analysis where we wanted to explore all potential associations, we did not correct for multiple comparisons and rather relied on effect sizes and summary statistics for interpretation. Spearman correlation was chosen to account for the small sample size of the patient group and for the non-normality of some symptoms variables (Supplementary Fig. 4 and Supplementary Table 7). To interpret the strength of correlations, we used Cohen’s guidelines,^56^ which considered correlation coefficients of 0.1, 0.3 and 0.5 as small, medium and large in magnitude, respectively.

## Results

### Performances in psychophysical tests

#### Controls versus patients

The resulting mean and standard error (*SE*) for each test based on untransformed data after averaging the two test-retest scores and outlier removal can be found in Supplementary Table 4 for controls and patients separately.

**Figure 3:**
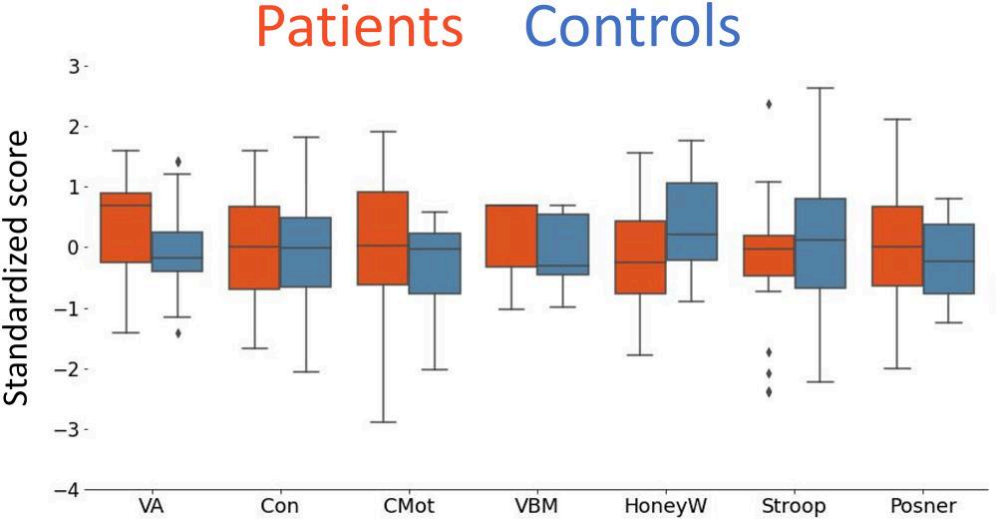
**Test variables barplots for patients (orange, *n* = 20) and controls (blue, *n* = 17).** High scores indicate better performance. Results from Student *t*-tests comparing group performances showed that controls tended to perform better than patients in the HoneyW (*t*(35) = −1.84, *P* > |*t*| = 0.08, *P* corrected for multiple comparison with Bonferroni–Holm = 0.53, *d* = −0.64). No other group differences were found. Detailed statistics are reported in Table 3. Acronyms: VA, Freiburg visual acuity; Con, contrast sensitivity; CMot, motion direction sensitivity; VBM, visual backward masking; HoneyW, Honeycomb white illusion.

When comparing patients versus controls, we found only one performance difference trend in HoneyW, with controls performing better than patients, that is, controls reported seeing barbs within a larger ellipse area compared to patients (*P* = 0.08, *d* = −0.64). The trend did, however, not survive correction for multiple comparisons (Table 3). In addition, the effect sizes for VA and VBM were larger than 0.5, suggesting that patients had a moderately better performance compared to controls in these two tests. Figure 3 displays barplots of the scores for all tests across patients and controls and clearly show the overlap in performance between the two groups. The plots show greater variance in the patients’ group for the CMot and Posner tests compared to the control group. However, for all other tests, the variances were comparable.

**Table 3:** Student's *t*-tests comparing psychophysical test performances between patients and controls

Variable	Statistics				
	*t*	*df*	*P*	*pBH*	*d*
**VA**	**1**.**54**	**34**	**0**.**13**	**0**.**76**	**0**.**52**
**Con**	0.08	33	0.94	1.0	0.03
**CMot**	0.96	34	0.34	1.0	0.32
**VBM**	**1**.**58**	**29**	**0**.**13**	**0**.**76**	**0**.**57**
**HoneyW**	**−1**.**84**	**31**	**0**.**08**	**0**.**53**	**−0**.**64**
**Stroop**	−1.05	35	0.30	1.0	−0.35
**Posner**	0.52	35	0.61	1.0	0.17

*pBH*-values were corrected for multiple comparisons with Bonferroni–Holm. High scores indicate better performance. Thus, a negative effect size indicates better performance for the controls compared to the VSS patients. Bold indicates effect sizes > 0.50.

#### Comorbidities

In general, comorbidities (i.e. tinnitus and migraine) did not influence the test performances (Supplementary Table 5). Patients without tinnitus tended to perform better in Con (*d* = 0.75, *P* = 0.06).

#### VS colour

**Figure 4:**
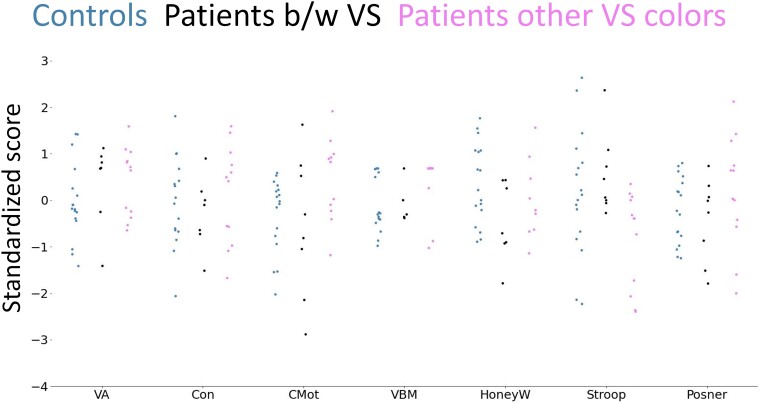
**Test variable strip plots for controls (blue, *n* = 17), patients with black and white (b/w) VS (black, *n* = 8) and patients with other VS colours (pink, *n* = 12).** High scores indicate better performance. Welch’s ANOVAs comparing group performances showed only one significant groups difference in the Stroop test (*F*(2, 20.51) = 5.28, *P* = 0.014, *η*^2^ = 0.19). Post-doc Welch’s *t*-test revealed that patients reporting other VS colours than black and white performed significantly worse in the Stroop test compared to patients with black and white VS (*t*(16.89) = 3.2, *P* > |*t*| = 5e−3, *P* corrected for multiple comparisons with Bonferroni–Holm = 0.02, *d* = 1.41), and, before correction for multiple comparisons, also compared to controls (*t*(26.76) = 2.12, *P* > |*t*| = 0.04, *P* corrected for multiple comparisons with Bonferroni–Holm = 0.07, *d* = 0.80). There was no performance difference between controls and patients with black and white VS (*t*(20.42) = −0.87, *P* > |*t*| = 0.40, *P* corrected for multiple comparisons with Bonferroni–Holm = 0.40, *d* = −0.32). Detailed statistics for Welch’s ANOVAs for the other tests are reported in Supplementary Table 6.

Figure 4 displays strip plots of the scores for all tests across controls, patients with black and white VS and patients with VS colours other than black and white. ANOVAs comparing test performances between these three groups showed a significant group effect only for the Stroop test (*F*(2, 20.51) = 5.28, *P* = 0.014, *η2* = 0.19; see Table S6 for the statistical results in all tests). Post-hoc tests showed that patients reporting other VS colours than black and white performed significantly worse in the Stroop test compared to patients with black and white VS (*t*(16.89) = 3.2, *P* = 5e−3, *P* corrected for multiple comparisons = 0.02, *d* = 1.41), and, before correction for multiple comparisons with Bonferroni–Holm, also compared to controls (*t*(26.76) = 2.12, *P* = 0.04, *P* corrected for multiple comparisons = 0.07, *d* = 0.80). There was no performance difference between controls and patients with black and white VS (*t*(20.42)=−0.87, *P* = 0.40, *P* corrected for multiple comparisons = 0.40, *d* = −0.32). Large effects were also found in the ANOVAs for CMot, VBM and HoneyW.

### Tests performance versus symptoms (in patients)

A total of 147 correlations were computed (i.e. seven psychophysical tests * 21 symptoms). The statistical results are documented in Table 4. Scatterplots of each correlation are presented in Supplementary Fig. 5.

**Table 4:** Spearman correlation (r, p) between visual test scores and symptoms

	VA	Con	CMot	VBM	HoneyW	Stroop	Posner
VSS year duration (age-corrected)	0.14, 0.56	**−0.55, 0.02**	−0.28, 0.26	*−0.35*, *0.2*	−0.18, 0.53	−0.06, 0.81	−0.22, 0.36
VS severity	−0.18, 0.45	**−0.7, 7.4e−4**	**−0.55, 0.01**	−0.19, 0.48	*−0.36*, *0.17*	0.03, 0.91	−0.09, 0.72
**VS characteristics** ^a^							
Density	−0.01, 0.97	**−0.74, 3.3e−4**	**−0.62, 4.9e−3**	−0.18, 0.51	*−0.36*, *0.17*	0.14, 0.55	0.03, 0.89
Speed	0.2, 0.41	−0.16, 0.51	0.05, 0.85	−0.1, 0.72	−0.28, 0.3	0.06, 0.8	*0.42*, *0.06*
Surface dependence	0.06, 0.8	**−0.5, 0.03**	**−0.6, 0.01**	−0.04, 0.88	−0.14, 0.6	*0.36*, *0.12*	0.06, 0.81
Distraction	0.01, 0.97	**−0.66, 2.1e−3**	**−0.7, 8.7e−4**	*−0.38*, *0.15*	*−0.49*, *0.05*	0.21, 0.37	0.08, 0.75
Time Course	*0.33*, *0.17*	*−0.45*, *0.05*	**−0.73, 3.8e−4**	−0.01, 0.97	−0.15, 0.58	0.21, 0.36	0.11, 0.64
Size	−0.22, 0.38	*−0.33*, *0.17*	−0.29, 0.23	−0.08, 0.78	−0.3, 0.26	0.09, 0.7	−0.0, 0.99
**VS severity** ^a^							
Outdoor sunny day	−0.19, 0.46	−0.0, 1.0	−0.09, 0.74	*−0.37*, *0.19*	*0.31, 0.28*	0.21, 0.41	−0.23, 0.36
Outdoor cloudy day	−0.14, 0.59	−0.14, 0.6	−0.1, 0.7	*−0.41*, *0.15*	−0.08, 0.79	0.02, 0.93	*0.35*, *0.15*
Outdoor rainy day	−0.25, 0.34	0.15, 0.56	0.28, 0.28	0.22, 0.45	0.1, 0.73	0.16, 0.52	0.14, 0.59
Indoor	*0.49, 0.04*	−0.12, 0.64	−0.01, 0.98	**0.55, 0.04**	−0.16, 0.59	0.21, 0.41	0.06, 0.8
Fluorescent lightning	0.27, 0.34	−0.25, 0.38	−0.12, 0.68	0.14, 0.65	−0.12, 0.7	0.14, 0.6	*−0.34*, *0.2*
Outdoor night time	0.26, 0.35	**0.73, 1.9e−3**	0.16, 0.57	*0.48*, *0.1*	*0.34, 0.28*	0.08, 0.78	0.21, 0.44
**VSS symptoms**							
Palinopsia	−0.22, 0.36	−0.15, 0.54	**−0.56, 0.01**	*−0.43, 0.09*	0.2, 0.46	*0.32, 0.17*	−0.25, 0.29
Blue field	−0.02, 0.93	−0.24, 0.33	−0.06, 0.82	0.02, 0.96	*0.34, 0.19*	*−0.32, 0.16*	−0.14, 0.56
Spontaneous photopsia	*−0.46*, *0.05*	**−0.56, 0.01**	*−0.45, 0.05*	*−0.32*, *0.23*	−0.1, 0.71	−0.05, 0.83	−0.18, 0.45
Floaters	*0.38*, *0.1*	*−0.33, 0.17*	−0.15, 0.53	0.17, 0.53	0.23, 0.4	−0.29, 0.21	0.08, 0.74
Flashes in darkness	−0.07, 0.77	*−0.48*, *0.04*	−0.25, 0.3	−0.08, 0.76	−0.14, 0.6	−0.07, 0.78	−0.06, 0.79
Photophobia	−0.06, 0.8	*−0.39*, *0.1*	−0.13, 0.6	−0.25, 0.36	0.08, 0.78	−0.04, 0.88	0.05, 0.85
Nyctalopia	−0.2, 0.43	−0.23, 0.36	0.19, 0.44	0.18, 0.52	−0.01, 0.97	−0.16, 0.52	−0.0, 0.98

Pairwise deletion was used. Effect sizes (*r*) and associated *P*-values (*P*) of Spearman correlations are reported. Italics and bold indicate moderate (i.e. 0.3 < *r* < 0.5) and large (i.e. *r* > 0.5) correlation strengths, respectively.

^a^Based on 30-day clinical diary questionnaire developed by Puledda and colleagues.^10^

In VSS symptoms and VS characteristics, a lower score indicated a lower impairment; whereas for VS severity and test performances, a lower score was worst (i.e. higher impairment/poorer performance).

Overall, higher clinical symptom severity was associated with poorer performance in the psychophysical tests, as indicated by mainly negative correlations between tests performance and (a) VSS year durations (6 out of 7); (b) VS symptom severity (6 out of 7), which was separated by the remaining VSS symptoms to facilitate comparison with the 30-day clinical diary scores which are related to VS and not VSS; (c) VS characteristics (27 out of 42); (d) VSS symptoms (37 out of 49); and by mainly positive correlations between tests performances and VS severity (24 out of 42). In particular, more severe spontaneous photopsia and distraction were strongly associated with lower performance in four out of the seven tests. Higher VS severity, more severe density and more severe VSS outdoor at night time were strongly associated with lower performance in three out of the seven tests.

## Discussion

### Study overview

In this study, we aimed to identify the specific visual characteristics VSS patients experience, with the goal of providing objective tests for diagnosis and for evaluating symptoms’ severity. Despite the large battery of psychophysical tests conducted, spanning many visual functions, we surprisingly only observed one performance difference trend between patients and controls, even before correction for multiple comparisons. Hence, there seem to be no objective deficits in the patients, except for the Honeycomb illusion. On the first glance, it is surprising that there are no deficits since VS is similar to external noise, which is known to affect performance in many tests.^57,58^ There are at least two possible explanations. Either the magnitude of the snow is too low to affect processing in the tests or the chosen tests were not sensitive to detect these deficits. The situation is reminiscent of tinnitus, where patients hear an internally generated tone that, interestingly, does not necessarily affect performance in all auditory tests.^59,60^ Another interesting observation is that within the patient population, higher symptom severity was associated with poorer test performances.

### Visual perception in VSS patients

First, not unexpectedly as used as inclusion criteria, we did not find a deficit in visual acuity in the patients in accordance with previous findings.^23^ In the Stroop and Posner tests, we surprisingly did not find performance differences between patients and controls despite fMRI studies indicated abnormality for VSS patients in regions though to be involved in these tests, such as parietal perfusion^34^ and static functional connectivity.^29^ Especially, the connections between higher visual brain regions and parietal regions, such as the superior parietal lobe and intraparietal sulcus, were higher in VSS patients compared to controls. For the Posner test, it has been shown that the parietal cortex, amongst other regions, differently responds to valid versus invalid trials.^61^ A gender bias is often reported in the Stroop test, with females outperforming males.^62^ Our study has more females in the controls compared to the patients. Therefore, the absence of a significant group difference strengthens our conclusion that Stroop performance is not deteriorated by VSS. Why there are discrepancies between tests performance and the fMRI studies remains unknown.

We hypothesized that the noisy dots in the contrast test adds up to the snow and, thus, disturbs performance. We could not confirm this hypothesis. One reason may be that the external noise masks the snow. Another explanation for this unexpected outcome could be that noisy dots had a short-term beneficial effect on VSS patients, analogous to how sound provides relief to individuals suffering from tinnitus.^63^

Patients showed a trend of lower performance compared to controls in the Honeycomb white illusion, although this trend did not survive correction for multiple comparisons. Hence, the finding may be a false positive; otherwise, the Honeycomb would potentially be an objective test, which in addition is easy to apply. If true, it may be that the small, peripherally presented barbs are a much weaker stimulus than the stimuli in the other test, which were all presented in the fovea, and hence are more vulnerable. Another explanation could be that patients with VSS show receptive field size alterations, which are known to grow with eccentricity along the visual stream.^64^ In particular, the low performance was driven by patients with black/white VS, suggesting that the processing along the retino-cortical pathway, including the lateral geniculate nucleus and higher visual brain regions, such as the lingual gyrus, is partially impaired. In this context, for nyctalopia, in which patients complain about poor vision in darker environments, has often been described in patients with retinal diseases (especially affecting the rod cells). Yet, the inability to adapt to light conditions might involve almost all components of the visual pathway.^65^ Up to 78% of VSS patients are affected by nyctalopia.^9^ So far, using intuitive colourimetry testing, it could be shown that patients with classic VS demonstrated that visual snow symptoms are exacerbated by colour modulation that selectively increased levels of S-cone excitation.^66^ Recently, Zaroban *et al.*^67^ in a small exploratory study demonstrated that patients with VS and VSS have normal retinal structure, but abnormal electrophysiology compared with control subjects. The increased b-wave and flicker amplitudes (assessed by full-field electroretinography) suggest increased responsiveness of the rod and cone photoreceptors and may contribute to VS pathophysiology. Taken together, our results give further support for a disturbance in early visual processing stations in patients with VSS. Future research may hence study receptive field, peripheral information processing and crowding in VSS patients.

Intriguingly, patients with black and white VS performed significantly better than those with other snow colours in the Stroop test, which was the sole test with colours. On the other hand, patients experiencing black and white VS tended to perform more poorly in the CMot, VBM and HoneyW tests compared to those reporting other colours of VS. Possibly, black and white VS involve a different pathway in the brain which should be elucidated in future studies assessing, in more detail, the electrophysiological abnormalities as well as the role of the konio-and parvo/magnocellular pathway and thalamocortical dysrhythmia.^6,68^ Clinically, it is known that patients with migraine show alterations in the retinogeniculostriate pathway,^69^ and thus the interaction between VSS, migraine and altered perception (using the HoneyW) should be studied in more detail. Importantly, these preliminary results, which need validation with a larger sample size, highlight the heterogeneity of VSS. They also imply that it may be better for future studies to separate patients according to VS colour report.

We would like to mention that since we did not find strong group differences, it is not surprising that correlations between the tests performances for patients were weak and of similar magnitudes as in controls (Supplementary Fig. 6). This result mirror previous findings showing that there is no evidence for one common factor underlying visual abilities, that is, that performance in one visual test is not predictive of performance in a different visual test.^70-72^

It is worth noting that the current study assessed visual test performance over a relatively short duration, during which patients were typically motivated to complete the tests. However, the discomfort and non-visual symptoms experienced by patients can have a detrimental impact on their well-being.^8^ Therefore, even if their visual abilities remain intact, these symptoms may lead to increased energy expenditure and long-term fatigue and discomfort.

### Relationship between visual perception and severity of symptoms

Overall, our results show that a better visual performance in the test battery inversely correlates with symptom severity (Table 4). Although the main focus was on the overall pattern of relationships, we believe that the following correlations are worth discussing. Better performance in Con and CMot correlates with a reduced VS severity reflected mainly in reduced VS characteristics (except for speed). Our data support the hypothesis of McKendrick *et al*.^20^ that extraction of a signal from noise is impaired by the presence of visual snow, although their results did not show a difference in the global form and motion tasks. Overall, patients with increased VS severity under low-light conditions appeared to perform better in the various tests, especially the contrast test. A better performance in VBM and CMot tests was associated with a reduction in palinopsia. Better performance in the Posner test correlated with faster VSS speed, suggesting that visual attention may be less impacted by higher frequency of flickering, which is in line with the finding of impaired dynamic contrast at low but not at higher frequencies.^22^ A better performance in Honeycomb was associated with a reduction in VSS distraction. In summary, the test batteries reflect, at least to a certain extent, the perceived severity of VS. However, the pattern needs to be replicated.

Notably, the correlations observed between visual test performances and symptoms align with those found between the symptoms themselves (Supplementary Fig. 7). In particular, VS severity strongly correlates with VSS symptoms and VS characteristics (except for speed), but does not correlate with the scores of VS severity under different light conditions (except for outdoor night time). Additionally, VSS symptoms show moderate correlations with VSS duration, VS characteristics (except for speed), but seldom with the scores of VS severity under different light conditions. Therefore, a strong VS, as described by high VS characteristics and rated by VS severity, does not necessarily result in more pronounced discomfort, as reflected in the generally low correlations with the scores of VS severity under different light conditions.

Understanding the relationship between visual abilities and symptoms severity may help to develop coping strategies for VSS (e.g. glasses that improve contrast sensitivity are likely to provide relief at night).

### Limitations

First, the sample size was relatively small, particularly when considering the numerous comparisons conducted. It is important to emphasize the exploratory nature of these correlations; the intention was to initiate an understanding of the overall pattern of relationships between patients’ visual abilities and symptoms. Second, patients’ visual abilities prior the onset of VSS are uncertain. Establishing a baseline for visual abilities is challenging since the onset of VSS is rapid.^2^ Therefore, our study cannot determine whether and to which extent baseline visual abilities influence the severity of symptoms or if symptom severity affects visual abilities upon the onset of VSS. Longitudinal studies involving treatment for VSS patients could help distinguish between these two scenarios, investigating whether successful treatments (resulting in reduced symptoms) lead to improvements in visual abilities. Third, we relied on self-reported symptoms. To facilitate a more objective evaluation of VSS severity, we are developing a visual snow simulator where the calibrated parameters will be comparable across subjects (https://visualsnowsim.epfl.ch/).

## Conclusions

Overall performance in various visual psychophysical tests is largely preserved in patients affected by visual snow syndrome. However, differences may arise in specific tests (such as the Honeycomb test), in the different subgroups of visual snow (e.g. black and white, flashing, transparent, etc.) or in the different additional visual and non-visual symptoms (e.g. palinopsia, enhanced entoptic phenomena, etc.). For the future, further investigations are necessary to confirm our preliminary and exploratory finding, and we would suggest to thoroughly phenotype patients with VSS to obtain homogenous groups. In tests reflecting cortical hyperexcitability, symptom severity is inversely correlated with test performance. Measures of visual perception may in the future help guide diagnosis and treatment effects in patients with visual snow syndrome.

## Supplementary Material

fcae341_Supplementary_Data

## Data Availability

Data will be made available upon reasonable request. Analyses were conducted in a Python 3.9.12 environment. The full code is accessible in the Supplementary material.
