# Supplementary material for: Understanding visual perception in visual snow syndrome: a battery of psychophysical tests plus the 30-day clinical diary
Source: Brain Commun. 2024 Sep 30;6(5):fcae341. doi: 10.1093/braincomms/fcae341 (PMC11474241; doi:10.1093/braincomms/fcae341)
Supplement: fcae341_Supplementary_Data [file fcae341_supplementary_data.zip › Supplementary_figures_and_tables.pdf]

Supplementary Table 1 – Number of outliers excluded based on test-retest

	VA	Con	CMot	VBM	HoneyW	Stroop	Posner
**Patients**	1	1	1	4	4	0	0
**Controls**	0	1	0	2	0	0	0

Please note that also when considering the median reaction time for the congruent and incongruent conditions of the Stroop and Posner tests, there were no outliers (not shown, but we mention it as we computed ICC31 for these variables). Acronyms: VA = visual acuity; Con = contrast sensitivity; CMot = coherent motion; VBM = visual backward masking, HoneyW = Honeycomb white illusion.

Supplementary Table 2 – Test-retest reliability of visual tests

Variable	Statistics				
	ICC31	*F*	*df1*	*df2*	*p*
**VA**	0.878	15.379	34	34	1.03e-12
**Con**	0.375	2.2	31	31	0.016
**CMot**	0.891	17.368	28	28	2.05e-11
**VBM**	0.954	42.83	29	29	4.61e-17
**HoneyW**	0.945	35.382	32	32	2.15e-17
**Stroop**	0.260	1.703	36	36	0.057
**Posner**	0.282	1.786	36	36	0.043
**StroopIncRT**	0.733	6.485	36	36	8.43e-8
**StroopConRT**	0.778	8.002	36	36	4.57e-9
**PosnerIncRT**	0.856	12.875	36	36	3.76e-12
**PosnerConRT**	0.879	15.508	36	36	2.01e-13

Intraclass correlation of type (3,1) were computed for each visual test. ICCs of VBM and HoneyW showed excellent reliability (i.e., ICCs > 0.90; Koo & Li, 2016), ICCs of VA, CMot, StroopIncRT, StroopConRT, PosnerIncRT, and PosnerConRT showed good reliability (i.e., ICCs between 0.75 and 0.90), while ICCs of Con, Stroop, and Posner showed poor reliability (i.e., ICCs < 0.50). New acronyms: StroopIncRT = median reaction time for the incongruent condition of the Stroop test, StroopConRT = median reaction time for the congruent condition of the Stroop test, PosnerIncRT = median reaction time for the incongruent condition of the Posner test, PosnerConRT = median reaction time for the congruent condition of the Posner test.

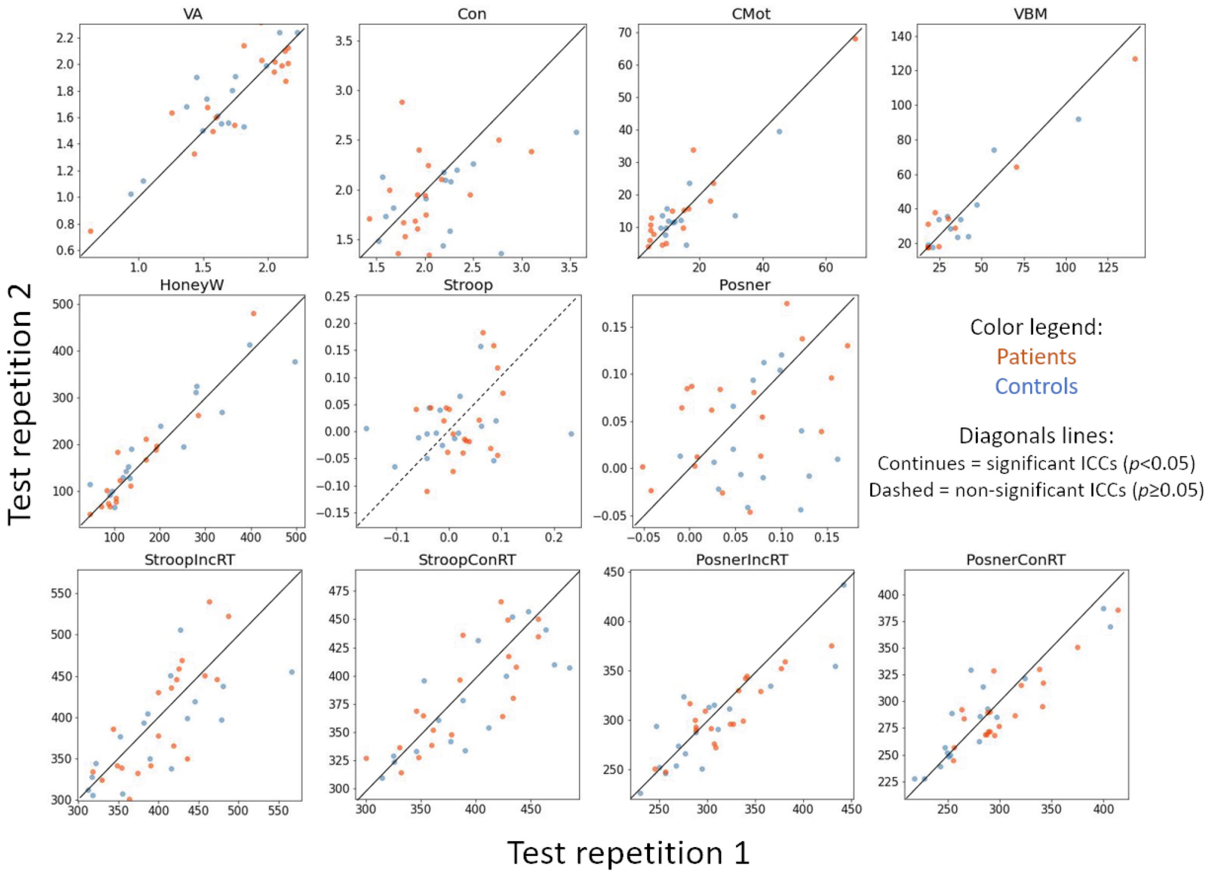

Supplementary Figure 1: Scatter plots were generated to visualize test-retest reliability. Points along the diagonal indicate that participants achieved the same score in the two test repetitions. In all scatterplots except Posner and Stroop, points clustered around the diagonal suggesting stable test-retest performance. Results from intraclass correlation of type (3,1) are reported in Supplementary Table 2. Dashed diagonals lines were used to indicate non-significant ICCs ($p \geq 0.05$). Blue dots represent controls' scores, orange dots represent patients' scores.

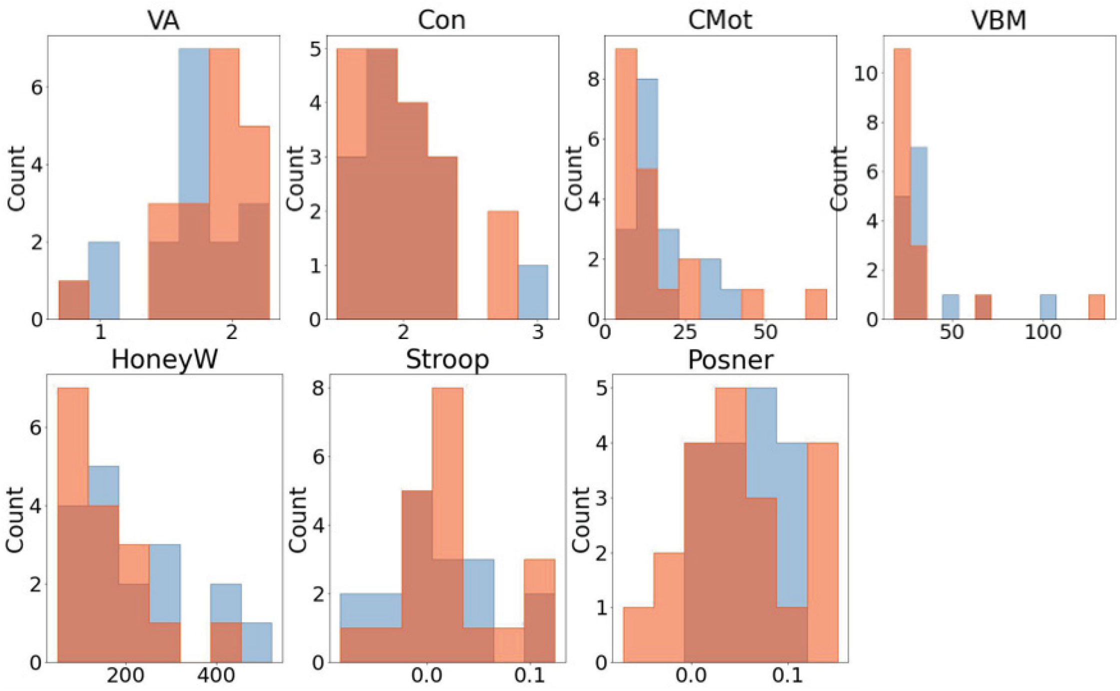

Supplementary Figure 2: Frequency histogram of scores distributions in each visual test for controls (blue) and patients (orange) after test-retest averages but before approximation to normal distribution and outlier removal.

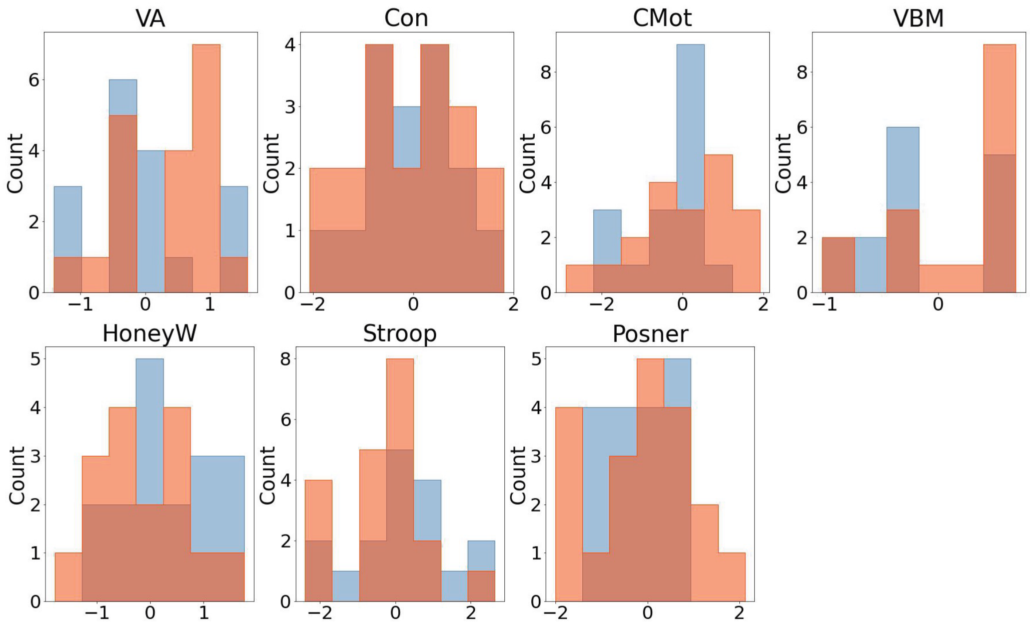

Supplementary Figure 3: Frequency histogram of scores distributions in each visual test for controls (blue) and patients (orange) after preprocessing. Scores were power transformed and

standardized, outliers were removed according to modified *z*-scores, and signs were flipped when needed so that low score indicates better performance for all tests.

Supplementary Table 3 - Statistics from Shapiro-Wilk tests, optimized lambda (λ) exponent for the power transformation, and number of outliers for each test

	VA	Con	CMot	VBM	HoneyW	Stroop	Posner
**SW statistic**	0.901*	0.931*	0.771*	0.619*	0.849*	0.935*	0.986
**SW statistic Z**	0.953	0.985	0.977	0.830*	0.971	0.951	0.986
**Optimized λ**	3.436	-2.451	0.055	-1.628	-0.176	-2.291	1.149
**Nb outliers**	0	0	0	0	0	0	0

The Shapiro-Wilk test on scores distributions for was run both before (SW statistic) and after (SW statistic Z) scores were transformed using a *Yeo-Johnson* power transformation with the optimized λ exponent, signs flipped so that high scores indicate better performance for all tests and outliers removed. Significant statistics (* $p < 0.05$) indicate a violation of the normality assumption.

Supplementary Table 4 – Mean and standard error of untransformed test scores after removal of outliers

	Control			Patients		
	M	*SE*	n	*M*	*SE*	n
**VA**	1.635	0.422	17	1.829	0.385	19
**Con**	2.016	0.373	16	2.005	0.345	19
**CMot**	16.951	9.850	17	16.365	16.227	19
**VBM**	34.961	21.90	15	31.471	30.079	16
**HoneyW**	220.918	135.9	17	149.949	98.417	16
**Stroop**	0.006	0.055	17	0.023	0.051	20
**Posner**	0.056	0.038	17	0.047	0.06	20

Low values indicate better performance in all but VA and HoneyW tests. Untransformed scores without outliers are reported (i.e., we delete the outliers removed through the pre-processing steps from the raw scores).

Supplementary Table 5 – Welch's *t*-tests comparing visual test performances between patients who had a specific comorbidity and patients who did not have it

Visual test	Variable	Statistics			
		t	*df*	*p*	*d*
**VA**	Tinnitus	-0.5	3.50	0.65	-0.39
	Migraine	0.15	14.91	0.88	0.07
**Con**	**Tinnitus**	**2.07**	**12.20**	**0.06**	**0.75**
	Migraine	0.33	16.99	0.75	0.14
**CMot**	**Tinnitus**	**1.76**	**7.97**	**0.12**	**0.75**
	Migraine	-0.60	15.73	0.55	-0.28
**VBM**	Tinnitus	0.38	3.15	0.73	0.23
	Migraine	0.49	9.11	0.64	0.27
**HoneyW**	Tinnitus	0.43	7.48	0.68	0.21
	Migraine	0.17	9.69	0.87	0.08
**Stroop**	Tinnitus	1.18	17.97	0.26	0.35
	Migraine	0.64	17.66	0.53	0.28
**Posner**	Tinnitus	-0.12	5.55	0.91	-0.06
	Migraine	-0.24	17.92	0.81	-0.10

9/20 patients had migraine, and 16/20 had tinnitus. Cohen (1988) interpreted effect sizes of 0.2, 0.5 and 0.8 as small, moderate and large. Bold indicates moderate to large effect sizes. Higher scores indicate better performance in the visual tests. Thus, a positive effect size indicates better performance for the patients without the comorbidity compared to patients with the comorbidity. *p*-values were not corrected for multiple comparisons to be more conservative and spot any influence of comorbidities on test performances.

Supplementary Table 6 – Welch's ANOVAs results comparing test performance between controls, patients with black/white VS, and patients with other VS colors than black/white

	dof1	*dof2*	F	*p-value*	η2
**VA**	2	15.62	1.14	0.35	0.07
**Con**	2	17.44	0.42	0.66	0.02
**CMot**	2	14.97	2.85	0.09	0.14
**VBM**	2	12.77	1.85	0.20	0.13
**HoneyW**	2	14.61	2.19	0.15	0.13
**Stroop**	2	20.51	5.28	0.01	0.19
**Posner**	2	16.13	0.83	0.45	0.06

η2 effect sizes of 0.01, 0.06, and 0.14 are interpreted as small, moderate, and large, respectively. Post-doc Welch's t-test for Stroop test are reported in the main text. Strip plots for each test variable are shown in Figure 4.

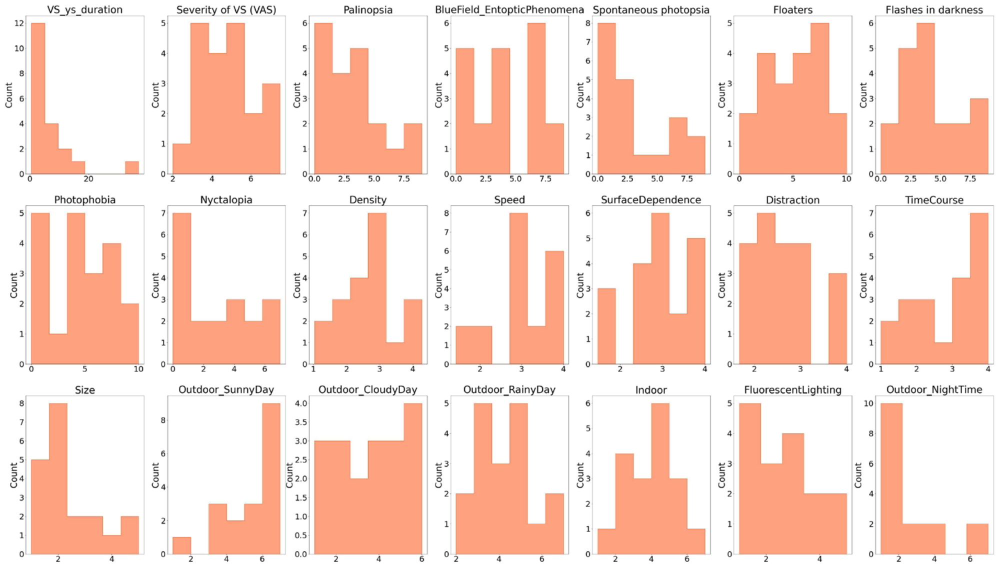

Supplementary Figure 4: Frequency histogram of patients' symptoms.

Supplementary Table 7 - Statistics from Shapiro-Wilk tests for patients' symptoms variables

	VSS ys duration	VS severity	Palinopsia	Blue field entoptic phenomena	Spontaneous photopsia	Floaters	Flashes in darkness
**SW statistic**	0.661*	0.956	0.907	0.918	0.836*	0.983	0.942
	**Photophobia**	**Nyctalopia**	**Density**	**Speed**	**Surface dependence**	**Distraction**	**Time course**
**SW statistic**	0.917	0.878*	0.961	0.901*	0.936	0.931	0.879*
	**Size**	**Outdoor sunny day**	**Outdoor cloudy day**	**Outdoor rainy day**	**Indoor**	**Fluorescent lightning**	**Outdoor night time**
**SW statistic**	0.867*	0.893*	0.896*	0.925	0.939	0.884*	0.799*

The Shapiro-Wilk test was run on all variables (SW statistic). No transformations were applied to these variables. Significant statistics (* $p < 0.05$) indicate a violation of the normality assumption.

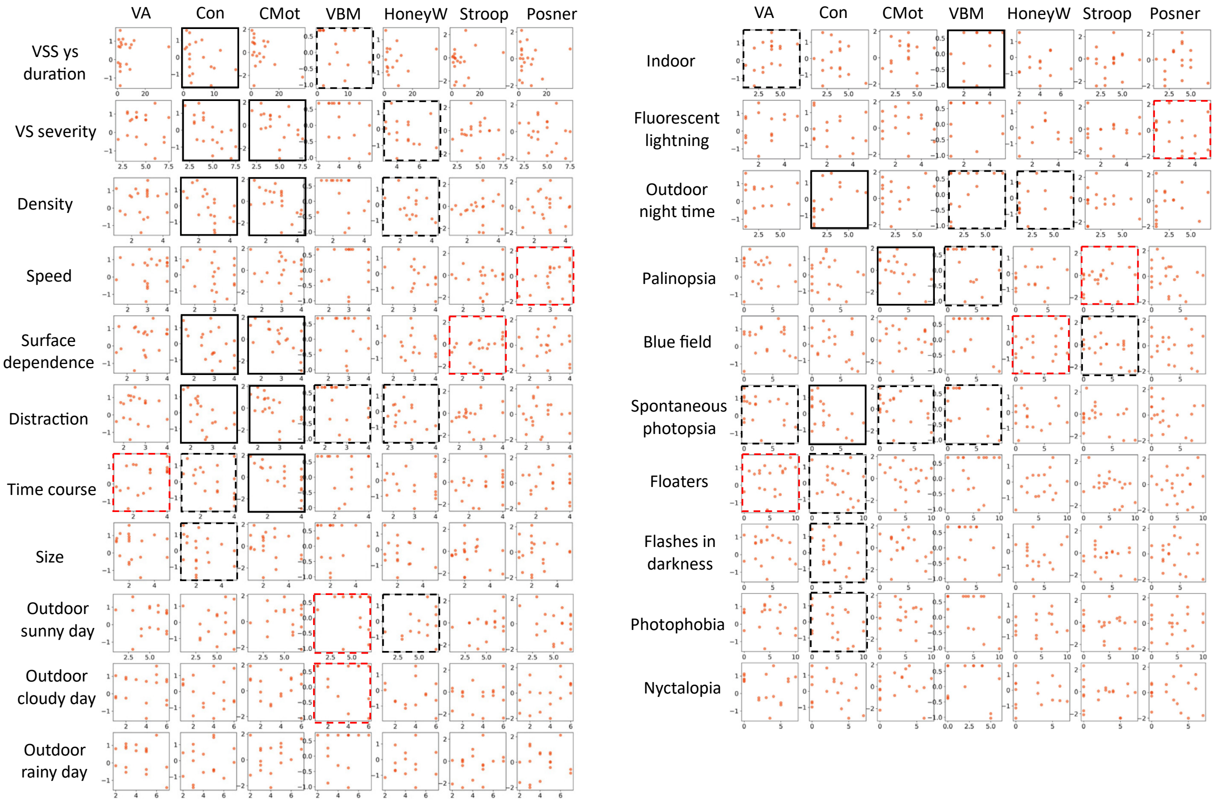

Supplementary Figure 5: Scatterplots between performances in the psychophysical tests and symptoms severity. Highlighted boxes with a continuous line show a significant Spearman correlation ($p < 0.05$). Highlighted boxes with a dashed line represent (non-significant) correlations larger than 0.3. Black is used to indicate a correlation where better performance in the test is associated with low symptom severity, red is used to indicate a correlation where better performance in the test is associated with higher symptom severity. Detailed statistics are reported in in the main manuscript (Table 4).

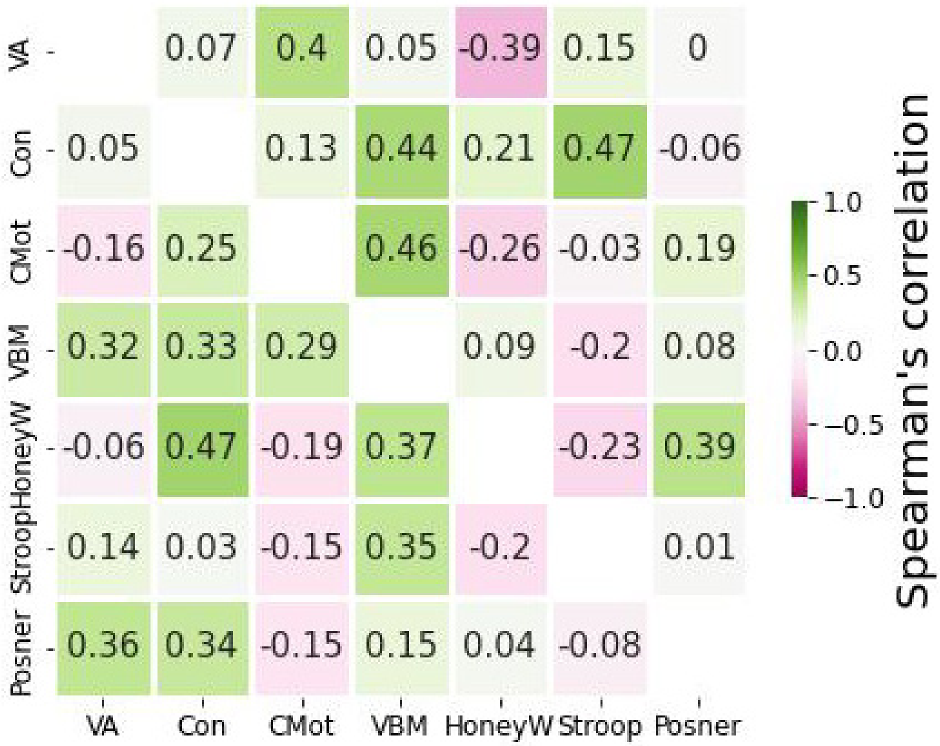

Supplementary Figure 6: Between psychophysical tests Spearman correlation coefficients for patients (lower part) and controls (upper part). The color scale ranging from pink to green represents effect sizes from r = -1 to r = 1 (white corresponds to r = 0). Pairwise deletion was used to compute correlations. Higher scores indicate better performance in all tests, so a positive correlation indicates similar performance across the two tests. The 25th, 50th and 75th percentiles of the correlation coefficients were -0.08, 0.14 and 0.33 for the VSS patients, and -0.03, 0.08 and 0.21 for the controls group. Thus, overall, the correlations were weak in both groups, providing evidence that there is no common factor underlying visual abilities, mirroring earlier findings in both young individuals (Cappe et al., 2014) and older adults (Garobbio, Pilz, Kunchulia, & Herzog, 2022; Shaqiri et al., 2019).

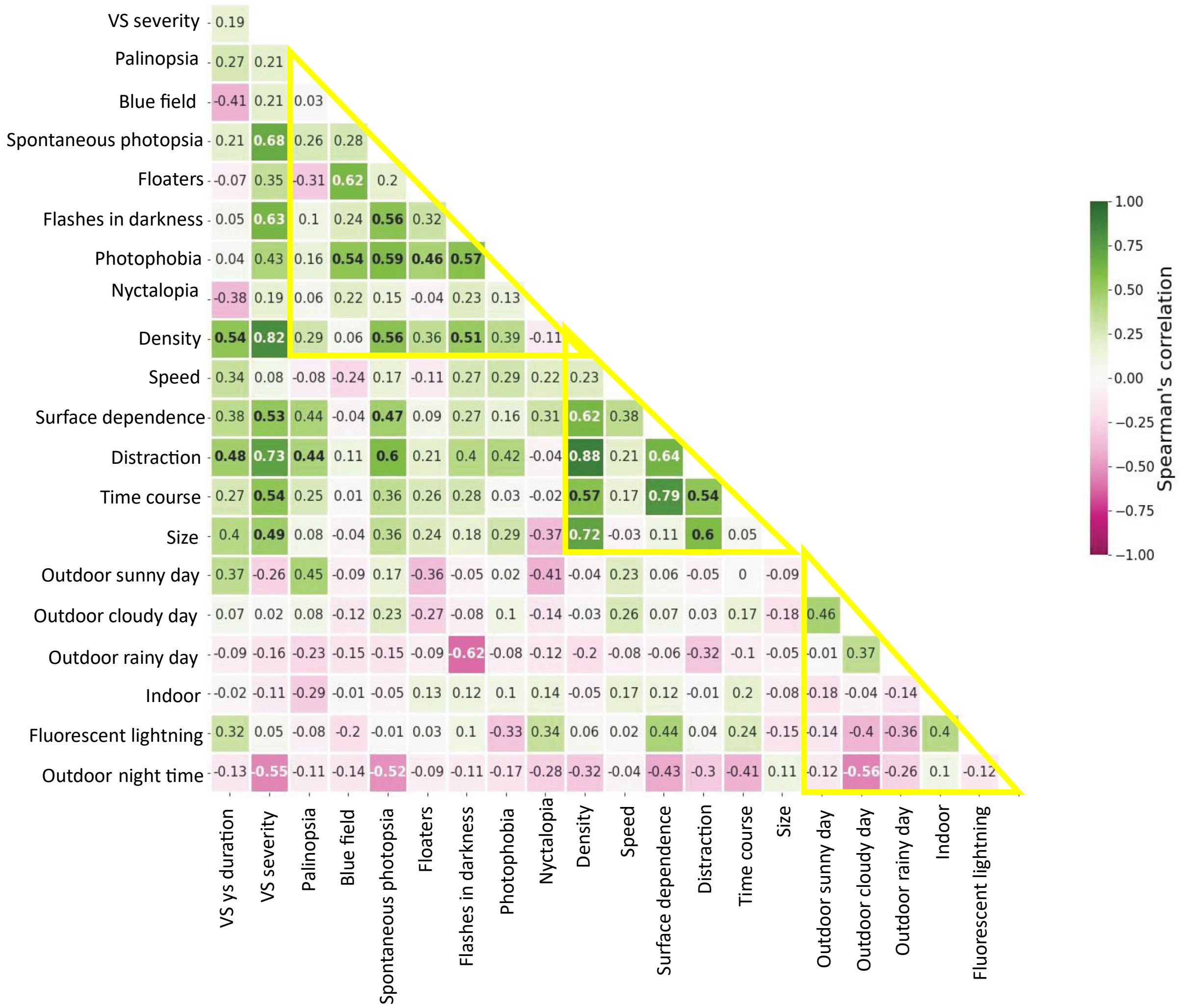

Supplementary Figure 7: Between-symptoms Spearman correlation coefficients. Bold numbers indicate significant results ($p < 0.05$, we did not correct for multiple comparisons). The color scale ranging from pink to green represents effect sizes from $r = -1$ to $r = 1$ (white corresponds to $r = 0$). Pairwise deletion was used to compute correlations. Yellow triangles indicate correlations between VSS symptoms (top triangle), VS characteristics (middle triangle), and VS severity under different light conditioning (bottom triangle). Lower scores indicate lower clinical severity for all but VS severity. The 25th, 50th and 75th percentiles of the absolute correlation coefficients were 0.09, 0.20 and 0.37. Thus, symptoms were generally moderately correlated with each other. Interestingly, symptoms correlated stronger with VS severity than with VSS duration. Some strong correlations were found between VSS symptoms as well as between VS characteristics, whereas correlations between VS severity under different light conditioning were weak. Stronger VSS symptoms showed in general stronger VS characteristics (as indicated by positive correlations) and stronger VS severity under different light conditioning (as indicated by negative correlations).
