# Supplementary material for: Understanding visual perception in visual snow syndrome: a battery of psychophysical tests plus the 30-day clinical diary
Source: Brain Commun. 2024 Sep 30;6(5):fcae341. doi: 10.1093/braincomms/fcae341 (PMC11474241; doi:10.1093/braincomms/fcae341)
Supplement: fcae341_Supplementary_Data [file fcae341_supplementary_data.zip › Supplementary_material_Code.htm]

1.GarobbioetAl\_code


# Code for manuscript "Understanding visual perception in visual snow syndrome: a battery of psychophysical tests plus the 30-day clinical diary"¶

### Garobbio S., Mazloum R., Rosio M., Popovova J., Schöpfer R., Fierz F.C., Disse L.R., Weber K.P., Schankin C.J., Michels L., Herzog M.H.¶

*****\*\****** Psychophysical tests

- Test-retest reliability
- Quick view of raw data
- Pre-processing
- T-tests controls vs patients
- Correlations between tests

*****\*\****** Symptoms data

*****\*\****** Demographical data

*****\*\****** Psychophysics vs. Symptoms

*****\*\****** Psychophysics vs. Demographical data

In [1]:

```
import pandas as pd
import numpy as np
import pingouin as pg
from sklearn.preprocessing import PowerTransformer
import scipy.stats as stats
import matplotlib.pyplot as plt
import seaborn as sb
import math as math

pd.set_option("display.max.columns", None)
```

```
C:\Users\garobbio\Anaconda3\lib\site-packages\outdated\utils.py:14: OutdatedPackageWarning: The package pingouin is out of date. Your version is 0.5.3, the latest is 0.5.4.
Set the environment variable OUTDATED_IGNORE=1 to disable these warnings.
  return warn(
```

# Psychophysical tests¶

In [2]:

```
df_Visit1_rep1 = pd.read_excel('./0.ZurichDataset_Raw.xlsx', sheet_name='Visit1_Repetition1', header = 0) 
df_Visit1_rep2 = pd.read_excel('./0.ZurichDataset_Raw.xlsx', sheet_name='Visit1_Repetition2', header = 0)

variables = ['VA', 'Con', 'CMot', 'VBM', 'HoneyW', 'Stroop', 'Posner']
StroopPosner = ['StroopIncRT', 'StroopConRT', 'PosnerIncRT', 'PosnerConRT']
allvariables = ['VA', 'Con', 'CMot', 'VBM', 'HoneyW', 'Stroop', 'Posner', 'StroopIncRT', 'StroopConRT', 'PosnerIncRT', 'PosnerConRT']
Group_labels = ['Controls','Patients']
Group_colors = ['steelblue', 'orangered']
Group_IDs = [90, 10] #Just be careful that in dataframe GroupID has first 10(patients) then 90(controls)
```

In [3]:

```
df_Visit1_rep1.head()
```

Out[3]:

|  | HoneyW | Posner | PosnerOut | PosnerIncRT | PosnerConRT | CMot | VA | Stroop | StroopOut | StroopIncRT | StroopConRT | Con | VBM | GroupID | SubjectID |
| --- | --- | --- | --- | --- | --- | --- | --- | --- | --- | --- | --- | --- | --- | --- | --- |
| 0 | 71.13390 | 0.001738 | 0 | 288.0 | 287.5 | 17.928534 | NaN | 0.092764 | 1 | 423.0 | 385.5 | 1.758024 | 52.707285 | 10 | 10-101 |
| 1 | 135.02715 | -0.051128 | 1 | 324.0 | 341.0 | 4.586555 | 1.946265 | 0.007580 | 1 | 463.5 | 460.0 | 1.918331 | 18.000002 | 10 | 10-102 |
| 2 | 419.07310 | -0.033083 | 0 | 327.0 | 338.0 | 14.802832 | 2.114367 | 0.032573 | 2 | 390.0 | 377.5 | 2.380323 | 18.000002 | 10 | 10-103 |
| 3 | 193.34500 | 0.069725 | 3 | 282.0 | 263.0 | 9.474165 | 1.531561 | 0.026729 | 0 | 436.0 | 424.5 | 1.775233 | 18.000002 | 10 | 10-104 |
| 4 | 89.78595 | 0.143646 | 1 | 339.5 | 294.0 | 3.830417 | 0.624752 | 0.029169 | 4 | 400.0 | 388.5 | 1.998052 | 18.000030 | 10 | 10-105 |

In [4]:

```
df_Visit1_rep2.head()
```

Out[4]:

|  | HoneyW | Posner | PosnerOut | PosnerIncRT | PosnerConRT | CMot | VA | Stroop | StroopOut | StroopIncRT | StroopConRT | Con | VBM | GroupID | SubjectID |
| --- | --- | --- | --- | --- | --- | --- | --- | --- | --- | --- | --- | --- | --- | --- | --- |
| 0 | 67.89620 | 0.087111 | 1 | 293.5 | 269.0 | 33.702928 | 2.039291 | 0.117507 | 1 | 446.0 | 396.5 | 2.887185 | 18.000070 | 10 | 10-101 |
| 1 | 111.44720 | 0.001691 | 0 | 296.0 | 295.5 | 9.028336 | 2.319045 | -0.003697 | 1 | 540.0 | 542.0 | 1.612247 | 18.000005 | 10 | 10-102 |
| 2 | 582.68155 | -0.108453 | 2 | 296.5 | 330.5 | 9.642635 | 2.448534 | -0.017391 | 3 | 342.0 | 348.0 | NaN | 18.000000 | 10 | 10-103 |
| 3 | 196.14430 | 0.080394 | 1 | 317.0 | 292.5 | 4.823594 | 1.678023 | -0.039216 | 1 | 350.0 | 364.0 | 1.673792 | 18.000000 | 10 | 10-104 |
| 4 | 67.93605 | 0.038748 | 1 | 342.0 | 329.0 | 4.051669 | 0.747690 | -0.013857 | 1 | 430.0 | 436.0 | 1.943180 | 51.374157 | 10 | 10-105 |

## Look at outliers Posner and Stroop, then delete the columns from dataset¶

In [5]:

```
print(df_Visit1_rep1['PosnerOut'].mean())
print(df_Visit1_rep2['PosnerOut'].mean())
print(df_Visit1_rep1['StroopOut'].mean())
print(df_Visit1_rep2['StroopOut'].mean())
```

```
0.6486486486486487
0.8378378378378378
0.9459459459459459
1.3243243243243243
```

In [6]:

```
df_Visit1_rep1.drop(df_Visit1_rep1.columns[[2,8]], axis=1, inplace=True)
df_Visit1_rep2.drop(df_Visit1_rep2.columns[[2,8]], axis=1, inplace=True)
```

## Test-retest reliability¶

In [7]:

```
# Get long dataframe format for ICCs
df_Visit1_rep1.insert(loc = 0, column = 'Repetition', value = 1)
df_Visit1_rep2.insert(loc = 0, column = 'Repetition', value = 2)
df_long = pd.concat([df_Visit1_rep1, df_Visit1_rep2]) 
df_long = df_long.reset_index(drop = True)
```

In [8]:

```
df_long.head()
```

Out[8]:

|  | Repetition | HoneyW | Posner | PosnerIncRT | PosnerConRT | CMot | VA | Stroop | StroopIncRT | StroopConRT | Con | VBM | GroupID | SubjectID |
| --- | --- | --- | --- | --- | --- | --- | --- | --- | --- | --- | --- | --- | --- | --- |
| 0 | 1 | 71.13390 | 0.001738 | 288.0 | 287.5 | 17.928534 | NaN | 0.092764 | 423.0 | 385.5 | 1.758024 | 52.707285 | 10 | 10-101 |
| 1 | 1 | 135.02715 | -0.051128 | 324.0 | 341.0 | 4.586555 | 1.946265 | 0.007580 | 463.5 | 460.0 | 1.918331 | 18.000002 | 10 | 10-102 |
| 2 | 1 | 419.07310 | -0.033083 | 327.0 | 338.0 | 14.802832 | 2.114367 | 0.032573 | 390.0 | 377.5 | 2.380323 | 18.000002 | 10 | 10-103 |
| 3 | 1 | 193.34500 | 0.069725 | 282.0 | 263.0 | 9.474165 | 1.531561 | 0.026729 | 436.0 | 424.5 | 1.775233 | 18.000002 | 10 | 10-104 |
| 4 | 1 | 89.78595 | 0.143646 | 339.5 | 294.0 | 3.830417 | 0.624752 | 0.029169 | 400.0 | 388.5 | 1.998052 | 18.000030 | 10 | 10-105 |

In [9]:

```
fig, axs = plt.subplots(2, 4, sharey=False, sharex=False) 
   
for var, ax in zip(variables, axs.reshape(-1)):
    xC_ = df_Visit1_rep1.loc[(df_Visit1_rep1['GroupID']==90), [var]].values
    yC_ = df_Visit1_rep2.loc[(df_Visit1_rep2['GroupID']==90), [var]].values
    xP_ = df_Visit1_rep1.loc[(df_Visit1_rep1['GroupID']==10), [var]].values
    yP_ = df_Visit1_rep2.loc[(df_Visit1_rep2['GroupID']==10), [var]].values
    fig.set_figheight(10)
    fig.set_figwidth(20)
    ax.scatter(xC_,yC_, color = 'steelblue', alpha = 0.6)
    ax.scatter(xP_,yP_, color = 'orangered', alpha = 0.6)
    ax.set_title(var, fontsize = 20)
   
    #ax.set_xlabel("first testing", fontsize=18)
    #ax.set_ylabel("second testing", fontsize=18)
    ax.tick_params(axis='both', which='both', labelsize=15, reset=True, bottom=True, top=False, left=True, right=False, labelbottom=True, labeltop=False, labelleft=True, labelright=False)
    xmin, xmax, ymin, ymax = ax.axis()
    ax.set_ylim([xmin,xmax])
    
    fig.tight_layout()
               
#plt.savefig('./trtRaw.jpg')
```

In [10]:

```
def icc(data, targets, raters, ratings): #Need long dataframe format
    icc_results = pg.intraclass_corr(data=data, targets=targets, raters=raters, ratings=ratings, nan_policy='omit').round(3)
    icc31 = icc_results.loc[2,['ICC', 'F', 'df1', 'df2', 'pval']].copy()
    icc31 = icc31.to_frame().T
    icc31 = icc31.rename(columns={'ICC':'ICC31'})
    icc31.insert(loc=0, column='Variable:', value=ratings)
    return icc31

## Use matlab for absolute ICC31
```

In [11]:

```
df_results = pd.DataFrame()

# Both groups merged
for var in variables:
    result = icc(df_long, 'SubjectID', 'Repetition', var)
    
    #gather all results together 
    df_results = pd.concat([df_results, result], axis = 0)

df_results = df_results.set_index('Variable:')  
df_results
```

Out[11]:

|  | ICC31 | F | df1 | df2 | pval |
| --- | --- | --- | --- | --- | --- |
| Variable: |  |  |  |  |  |
| VA | 0.824 | 10.388 | 35 | 35 | 0.0 |
| Con | 0.3 | 1.858 | 33 | 33 | 0.04 |
| CMot | 0.786 | 8.327 | 29 | 29 | 0.0 |
| VBM | 0.84 | 11.471 | 35 | 35 | 0.0 |
| HoneyW | 0.855 | 12.788 | 36 | 36 | 0.0 |
| Stroop | 0.26 | 1.703 | 36 | 36 | 0.057 |
| Posner | 0.282 | 1.786 | 36 | 36 | 0.043 |

In [12]:

```
## ICCs for Stroop, Posner RTs

df_results = pd.DataFrame()

# Both groups merged
for var in StroopPosner:
    result = icc(df_long, 'SubjectID', 'Repetition', var)
    
    #gather all results together 
    df_results = pd.concat([df_results, result], axis = 0)

df_results = df_results.set_index('Variable:')  
df_results
```

Out[12]:

|  | ICC31 | F | df1 | df2 | pval |
| --- | --- | --- | --- | --- | --- |
| Variable: |  |  |  |  |  |
| StroopIncRT | 0.733 | 6.485 | 36 | 36 | 0.0 |
| StroopConRT | 0.778 | 8.002 | 36 | 36 | 0.0 |
| PosnerIncRT | 0.856 | 12.875 | 36 | 36 | 0.0 |
| PosnerConRT | 0.879 | 15.508 | 36 | 36 | 0.0 |

In [13]:

```
df_results = pd.DataFrame()  

# for groups separated
for group in Group_IDs:
    df_icc = pd.DataFrame()
    for var in allvariables:
        result = icc(df_long.loc[(df_long['GroupID']==group)], 'SubjectID', 'Repetition', var)
        
        # gather results together
        df_icc = pd.concat([df_icc, result], axis = 0)
    
    # gather groups together
    df_results = pd.concat([df_results, df_icc], axis=0)
        
df_results.index = [['Controls','','','','','','','','','','','Patients','','','','','','','','','','']]
df_results
```

Out[13]:

|  | Variable: | ICC31 | F | df1 | df2 | pval |
| --- | --- | --- | --- | --- | --- | --- |
| Controls | VA | 0.868 | 14.145 | 16 | 16 | 0.0 |
|  | Con | 0.119 | 1.27 | 14 | 14 | 0.33 |
|  | CMot | 0.753 | 7.096 | 12 | 12 | 0.001 |
|  | VBM | 0.865 | 13.8 | 15 | 15 | 0.0 |
|  | HoneyW | 0.937 | 30.594 | 16 | 16 | 0.0 |
|  | Stroop | 0.167 | 1.402 | 16 | 16 | 0.253 |
|  | Posner | -0.134 | 0.763 | 16 | 16 | 0.703 |
|  | StroopIncRT | 0.715 | 6.014 | 16 | 16 | 0.0 |
|  | StroopConRT | 0.782 | 8.157 | 16 | 16 | 0.0 |
|  | PosnerIncRT | 0.858 | 13.104 | 16 | 16 | 0.0 |
|  | PosnerConRT | 0.908 | 20.857 | 16 | 16 | 0.0 |
| Patients | VA | 0.767 | 7.575 | 18 | 18 | 0.0 |
|  | Con | 0.324 | 1.958 | 18 | 18 | 0.082 |
|  | CMot | 0.81 | 9.527 | 16 | 16 | 0.0 |
|  | VBM | 0.676 | 5.176 | 19 | 19 | 0.0 |
|  | HoneyW | 0.816 | 9.844 | 19 | 19 | 0.0 |
|  | Stroop | 0.343 | 2.043 | 19 | 19 | 0.064 |
|  | Posner | 0.568 | 3.629 | 19 | 19 | 0.004 |
|  | StroopIncRT | 0.757 | 7.229 | 19 | 19 | 0.0 |
|  | StroopConRT | 0.792 | 8.611 | 19 | 19 | 0.0 |
|  | PosnerIncRT | 0.846 | 11.959 | 19 | 19 | 0.0 |
|  | PosnerConRT | 0.854 | 12.717 | 19 | 19 | 0.0 |

### Look at outlier removal on the difference rep 1-rep2 (i.e., bad test-retest)¶

In [14]:

```
#we generate a new dataframe that contains the difference between the 1st and 2nd testing
df_diff = df_Visit1_rep2[allvariables].subtract(df_Visit1_rep1[allvariables])
#We add Subject ID column at the beginning
df_diff.insert(0,'SubjectID', df_Visit1_rep1['SubjectID'].copy()) 
df_diff.head()
```

Out[14]:

|  | SubjectID | VA | Con | CMot | VBM | HoneyW | Stroop | Posner | StroopIncRT | StroopConRT | PosnerIncRT | PosnerConRT |
| --- | --- | --- | --- | --- | --- | --- | --- | --- | --- | --- | --- | --- |
| 0 | 10-101 | NaN | 1.129161 | 15.774394 | -34.707215 | -3.23770 | 0.024743 | 0.085373 | 23.0 | 11.0 | 5.5 | -18.5 |
| 1 | 10-102 | 0.372780 | -0.306084 | 4.441782 | 0.000003 | -23.57995 | -0.011277 | 0.052818 | 76.5 | 82.0 | -28.0 | -45.5 |
| 2 | 10-103 | 0.334166 | NaN | -5.160197 | -0.000002 | 163.60845 | -0.049965 | -0.075370 | -48.0 | -29.5 | -30.5 | -7.5 |
| 3 | 10-104 | 0.146462 | -0.101441 | -4.650571 | -0.000001 | 2.79930 | -0.065944 | 0.010669 | -86.0 | -60.5 | 35.0 | 29.5 |
| 4 | 10-105 | 0.122938 | -0.054871 | 0.221252 | 33.374127 | -21.84990 | -0.043026 | -0.104898 | 30.0 | 47.5 | 2.5 | 35.0 |

In [15]:

```
def modified_z_score(df, variables):
    
    df_temp = df.copy()
    
    for col in variables:
        med = df[col].median()
        dev_from_median = df[col] - med
        MAD = np.nanmedian(np.abs(dev_from_median))
        MeanAD = np.nanmean(np.abs(dev_from_median))
        
        if MAD != 0:
            Zm = 0.6745 * dev_from_median / MAD
        elif MAD == 0:
            Zm = dev_from_median / 1.253314*MeanAD
        
        df_temp[col] = Zm
        
    return df_temp
```

In [16]:

```
df_diff_Zm = modified_z_score(df_diff, allvariables)
```

In [17]:

```
# see outliers for each variable
for var in allvariables:
    if len(df_diff_Zm.loc[np.abs(df_diff_Zm[var]) > 3.5]) > 0:
        print("Subjects with outliers for "+var)
        display(df_diff_Zm.loc[np.abs(df_diff_Zm[var]) > 3.5])
```

```
Subjects with outliers for VA
```

|  | SubjectID | VA | Con | CMot | VBM | HoneyW | Stroop | Posner | StroopIncRT | StroopConRT | PosnerIncRT | PosnerConRT |
| --- | --- | --- | --- | --- | --- | --- | --- | --- | --- | --- | --- | --- |
| 5 | 10-106 | 4.579405 | -0.978067 | -0.177469 | 1.986304 | -0.392621 | -0.859529 | -0.682489 | -0.194174 | 0.74195 | -1.372259 | -0.6745 |

```
Subjects with outliers for Con
```

|  | SubjectID | VA | Con | CMot | VBM | HoneyW | Stroop | Posner | StroopIncRT | StroopConRT | PosnerIncRT | PosnerConRT |
| --- | --- | --- | --- | --- | --- | --- | --- | --- | --- | --- | --- | --- |
| 7 | 10-108 | -0.597761 | -14.439824 | NaN | -9.302789 | -0.164436 | 1.415658 | -0.724534 | 0.940212 | 0.084312 | -2.070017 | -1.224093 |
| 29 | 90-109 | 0.726557 | -5.359077 | 0.316926 | -0.370264 | 0.346659 | 0.843344 | -1.813804 | 0.367909 | -0.033725 | -1.628103 | 0.074944 |

```
Subjects with outliers for CMot
```

|  | SubjectID | VA | Con | CMot | VBM | HoneyW | Stroop | Posner | StroopIncRT | StroopConRT | PosnerIncRT | PosnerConRT |
| --- | --- | --- | --- | --- | --- | --- | --- | --- | --- | --- | --- | --- |
| 9 | 10-110 | -0.605203 | 0.98656 | 6.802202 | -1.81844 | -0.546901 | 1.049072 | 0.0 | -0.429227 | -1.517625 | -0.744276 | -0.999259 |

```
Subjects with outliers for VBM
```

|  | SubjectID | VA | Con | CMot | VBM | HoneyW | Stroop | Posner | StroopIncRT | StroopConRT | PosnerIncRT | PosnerConRT |
| --- | --- | --- | --- | --- | --- | --- | --- | --- | --- | --- | --- | --- |
| 0 | 10-101 | NaN | 3.458248 | 2.886417 | -4.443043 | -0.154362 | 0.444419 | 1.071630 | 0.592742 | 0.674500 | 0.674500 | -0.724463 |
| 4 | 10-105 | 0.563643 | 0.269129 | 0.004838 | 4.272414 | -0.630264 | -0.391731 | -1.249444 | 0.735818 | 1.905462 | 0.534948 | 1.948556 |
| 7 | 10-108 | -0.597761 | -14.439824 | NaN | -9.302789 | -0.164436 | 1.415658 | -0.724534 | 0.940212 | 0.084312 | -2.070017 | -1.224093 |
| 19 | 10-120 | -0.806145 | -0.274068 | 1.097277 | -12.067923 | -0.766273 | -0.552186 | 0.866194 | -0.725598 | -0.421563 | 0.302362 | -0.899333 |
| 31 | 90-111 | -0.078094 | 0.821794 | -0.109967 | -8.129894 | 0.883863 | 2.152711 | -0.624728 | 0.572303 | -0.876850 | -0.069776 | 0.349741 |
| 32 | 90-112 | -2.504589 | -2.223165 | NaN | -25.069649 | 1.327788 | 1.343803 | -1.244733 | 1.727129 | 1.298412 | -3.209690 | -1.623796 |

```
Subjects with outliers for HoneyW
```

|  | SubjectID | VA | Con | CMot | VBM | HoneyW | Stroop | Posner | StroopIncRT | StroopConRT | PosnerIncRT | PosnerConRT |
| --- | --- | --- | --- | --- | --- | --- | --- | --- | --- | --- | --- | --- |
| 2 | 10-103 | 1.640451 | NaN | -0.992200 | 0.000013 | 4.111784 | -0.477339 | -0.889239 | -0.858455 | -0.691362 | -1.000121 | -0.174870 |
| 8 | 10-109 | -0.068926 | 0.488228 | -1.056150 | 1.680870 | -4.370072 | 0.499305 | 1.097820 | 0.807356 | 0.994887 | 0.604724 | -1.049222 |
| 14 | 10-115 | -1.069413 | -0.157460 | NaN | 0.000013 | -3.868512 | 1.097825 | 0.219104 | 0.531424 | -0.134900 | -0.790793 | -1.174130 |
| 18 | 10-119 | -0.443258 | 1.192507 | 0.262367 | -0.000004 | -9.100624 | -0.298873 | 0.083343 | 0.459886 | 1.214100 | 0.000000 | -0.299778 |

### Delete these subjects' data completely¶

- If I look at psychopmetric fct, indeed not stable subjects

In [18]:

```
# remove the outliers
df_Visit1_rep1.loc[5,'VA'] = np.nan
df_Visit1_rep2.loc[5,'VA'] = np.nan

df_Visit1_rep1.loc[7,'Con'] = np.nan
df_Visit1_rep2.loc[7,'Con'] = np.nan
df_Visit1_rep1.loc[29,'Con'] = np.nan
df_Visit1_rep2.loc[29,'Con'] = np.nan

df_Visit1_rep1.loc[9,'CMot'] = np.nan
df_Visit1_rep2.loc[9,'CMot'] = np.nan

df_Visit1_rep1.loc[0,'VBM'] = np.nan
df_Visit1_rep2.loc[0,'VBM'] = np.nan
df_Visit1_rep1.loc[4,'VBM'] = np.nan
df_Visit1_rep2.loc[4,'VBM'] = np.nan
df_Visit1_rep1.loc[7,'VBM'] = np.nan
df_Visit1_rep2.loc[7,'VBM'] = np.nan
df_Visit1_rep1.loc[19,'VBM'] = np.nan
df_Visit1_rep2.loc[19,'VBM'] = np.nan
df_Visit1_rep1.loc[31,'VBM'] = np.nan
df_Visit1_rep2.loc[31,'VBM'] = np.nan
df_Visit1_rep1.loc[32,'VBM'] = np.nan
df_Visit1_rep2.loc[32,'VBM'] = np.nan

df_Visit1_rep1.loc[2,'HoneyW'] = np.nan
df_Visit1_rep2.loc[2,'HoneyW'] = np.nan
df_Visit1_rep1.loc[8,'HoneyW'] = np.nan
df_Visit1_rep2.loc[8,'HoneyW'] = np.nan
df_Visit1_rep1.loc[14,'HoneyW'] = np.nan
df_Visit1_rep2.loc[14,'HoneyW'] = np.nan
df_Visit1_rep1.loc[18,'HoneyW'] = np.nan
df_Visit1_rep2.loc[18,'HoneyW'] = np.nan
```

In [19]:

```
fig, axs = plt.subplots(3, 4, sharey=False, sharex=False) 
   
for var, ax in zip(allvariables, axs.reshape(-1)):
    xC_ = df_Visit1_rep1.loc[(df_Visit1_rep1['GroupID']==90), [var]].values
    yC_ = df_Visit1_rep2.loc[(df_Visit1_rep2['GroupID']==90), [var]].values
    xP_ = df_Visit1_rep1.loc[(df_Visit1_rep1['GroupID']==10), [var]].values
    yP_ = df_Visit1_rep2.loc[(df_Visit1_rep2['GroupID']==10), [var]].values
    fig.set_figheight(15)
    fig.set_figwidth(20)
    ax.scatter(xC_,yC_, color = 'steelblue', alpha = 0.6)
    ax.scatter(xP_,yP_, color = 'orangered', alpha = 0.6)
    ax.set_title(var, fontsize = 20)
   
    #ax.set_xlabel("first testing", fontsize=18)
    #ax.set_ylabel("second testing", fontsize=18)
    ax.tick_params(axis='both', which='both', labelsize=15, reset=True, bottom=True, top=False, left=True, right=False, labelbottom=True, labeltop=False, labelleft=True, labelright=False)
    xmin, xmax, ymin, ymax = ax.axis()
    ax.set_ylim([xmin,xmax])
    
    fig.tight_layout()
    
plt.savefig('./Figures/1.trt.jpg')
```

In [20]:

```
# Get long dataframe format for ICCs
df_long = pd.concat([df_Visit1_rep1, df_Visit1_rep2]) 
df_long = df_long.reset_index(drop = True)

df_results = pd.DataFrame()  

# for groups separated
for group in Group_IDs:
    df_icc = pd.DataFrame()
    for var in allvariables:
        result = icc(df_long.loc[(df_long['GroupID']==group)], 'SubjectID', 'Repetition', var)
        
        # gather results together
        df_icc = pd.concat([df_icc, result], axis = 0)
    
    # gather groups together
    df_results = pd.concat([df_results, df_icc], axis=0)
        
df_results.index = [['Controls','','','','','','','','','','','Patients','','','','','','','','','','']]
df_results
```

Out[20]:

|  | Variable: | ICC31 | F | df1 | df2 | pval |
| --- | --- | --- | --- | --- | --- | --- |
| Controls | VA | 0.868 | 14.145 | 16 | 16 | 0.0 |
|  | Con | 0.376 | 2.206 | 13 | 13 | 0.084 |
|  | CMot | 0.753 | 7.096 | 12 | 12 | 0.001 |
|  | VBM | 0.919 | 23.682 | 13 | 13 | 0.0 |
|  | HoneyW | 0.937 | 30.594 | 16 | 16 | 0.0 |
|  | Stroop | 0.167 | 1.402 | 16 | 16 | 0.253 |
|  | Posner | -0.134 | 0.763 | 16 | 16 | 0.703 |
|  | StroopIncRT | 0.715 | 6.014 | 16 | 16 | 0.0 |
|  | StroopConRT | 0.782 | 8.157 | 16 | 16 | 0.0 |
|  | PosnerIncRT | 0.858 | 13.104 | 16 | 16 | 0.0 |
|  | PosnerConRT | 0.908 | 20.857 | 16 | 16 | 0.0 |
| Patients | VA | 0.877 | 15.205 | 17 | 17 | 0.0 |
|  | Con | 0.401 | 2.339 | 17 | 17 | 0.044 |
|  | CMot | 0.939 | 32.037 | 15 | 15 | 0.0 |
|  | VBM | 0.973 | 72.761 | 15 | 15 | 0.0 |
|  | HoneyW | 0.947 | 36.624 | 15 | 15 | 0.0 |
|  | Stroop | 0.343 | 2.043 | 19 | 19 | 0.064 |
|  | Posner | 0.568 | 3.629 | 19 | 19 | 0.004 |
|  | StroopIncRT | 0.757 | 7.229 | 19 | 19 | 0.0 |
|  | StroopConRT | 0.792 | 8.611 | 19 | 19 | 0.0 |
|  | PosnerIncRT | 0.846 | 11.959 | 19 | 19 | 0.0 |
|  | PosnerConRT | 0.854 | 12.717 | 19 | 19 | 0.0 |

In [21]:

```
df_results = pd.DataFrame()

# Both groups merged
for var in allvariables:
    result = icc(df_long, 'SubjectID', 'Repetition', var)
    
    #gather all results together 
    df_results = pd.concat([df_results, result], axis = 0)

df_results = df_results.set_index('Variable:')  
df_results
```

Out[21]:

|  | ICC31 | F | df1 | df2 | pval |
| --- | --- | --- | --- | --- | --- |
| Variable: |  |  |  |  |  |
| VA | 0.878 | 15.379 | 34 | 34 | 0.0 |
| Con | 0.375 | 2.2 | 31 | 31 | 0.016 |
| CMot | 0.891 | 17.368 | 28 | 28 | 0.0 |
| VBM | 0.954 | 42.83 | 29 | 29 | 0.0 |
| HoneyW | 0.945 | 35.382 | 32 | 32 | 0.0 |
| Stroop | 0.26 | 1.703 | 36 | 36 | 0.057 |
| Posner | 0.282 | 1.786 | 36 | 36 | 0.043 |
| StroopIncRT | 0.733 | 6.485 | 36 | 36 | 0.0 |
| StroopConRT | 0.778 | 8.002 | 36 | 36 | 0.0 |
| PosnerIncRT | 0.856 | 12.875 | 36 | 36 | 0.0 |
| PosnerConRT | 0.879 | 15.508 | 36 | 36 | 0.0 |

In [22]:

```
pg.intraclass_corr(data=df_long, targets='SubjectID', raters='Repetition', ratings='PosnerConRT', nan_policy='omit')
```

Out[22]:

|  | Type | Description | ICC | F | df1 | df2 | pval | CI95% |
| --- | --- | --- | --- | --- | --- | --- | --- | --- |
| 0 | ICC1 | Single raters absolute | 0.878329 | 15.437819 | 36 | 37 | 1.149055e-13 | [0.78, 0.94] |
| 1 | ICC2 | Single random raters | 0.878363 | 15.508193 | 36 | 36 | 2.006868e-13 | [0.78, 0.94] |
| 2 | ICC3 | Single fixed raters | 0.878848 | 15.508193 | 36 | 36 | 2.006868e-13 | [0.78, 0.94] |
| 3 | ICC1k | Average raters absolute | 0.935224 | 15.437819 | 36 | 37 | 1.149055e-13 | [0.87, 0.97] |
| 4 | ICC2k | Average random raters | 0.935243 | 15.508193 | 36 | 36 | 2.006868e-13 | [0.88, 0.97] |
| 5 | ICC3k | Average fixed raters | 0.935518 | 15.508193 | 36 | 36 | 2.006868e-13 | [0.87, 0.97] |

### Compute average repetition 1 - repetition 2 to get df\_Visit1 final data to preprocess + analyze¶

In [23]:

```
df_Visit1 = df_Visit1_rep1[['SubjectID', 'GroupID']].copy()
df_Visit1['HoneyW'] = np.nanmean(pd.concat([df_Visit1_rep1['HoneyW'], df_Visit1_rep2['HoneyW']], axis=1), axis=1)
df_Visit1['Posner'] = np.nanmean(pd.concat([df_Visit1_rep1['Posner'], df_Visit1_rep2['Posner']], axis=1), axis=1)
df_Visit1['CMot'] = np.nanmean(pd.concat([df_Visit1_rep1['CMot'], df_Visit1_rep2['CMot']], axis=1), axis=1)
df_Visit1['VA'] = np.nanmean(pd.concat([df_Visit1_rep1['VA'], df_Visit1_rep2['VA']], axis=1), axis=1)
df_Visit1['Stroop'] = np.nanmean(pd.concat([df_Visit1_rep1['Stroop'], df_Visit1_rep2['Stroop']], axis=1), axis=1)
df_Visit1['Con'] = np.nanmean(pd.concat([df_Visit1_rep1['Con'], df_Visit1_rep2['Con']], axis=1), axis=1)
df_Visit1['VBM'] = np.nanmean(pd.concat([df_Visit1_rep1['VBM'], df_Visit1_rep2['VBM']], axis=1), axis=1)
df_Visit1.head()
```

```
C:\Users\garobbio\AppData\Local\Temp\ipykernel_2596\770145647.py:2: RuntimeWarning: Mean of empty slice
  df_Visit1['HoneyW'] = np.nanmean(pd.concat([df_Visit1_rep1['HoneyW'], df_Visit1_rep2['HoneyW']], axis=1), axis=1)
C:\Users\garobbio\AppData\Local\Temp\ipykernel_2596\770145647.py:4: RuntimeWarning: Mean of empty slice
  df_Visit1['CMot'] = np.nanmean(pd.concat([df_Visit1_rep1['CMot'], df_Visit1_rep2['CMot']], axis=1), axis=1)
C:\Users\garobbio\AppData\Local\Temp\ipykernel_2596\770145647.py:5: RuntimeWarning: Mean of empty slice
  df_Visit1['VA'] = np.nanmean(pd.concat([df_Visit1_rep1['VA'], df_Visit1_rep2['VA']], axis=1), axis=1)
C:\Users\garobbio\AppData\Local\Temp\ipykernel_2596\770145647.py:7: RuntimeWarning: Mean of empty slice
  df_Visit1['Con'] = np.nanmean(pd.concat([df_Visit1_rep1['Con'], df_Visit1_rep2['Con']], axis=1), axis=1)
C:\Users\garobbio\AppData\Local\Temp\ipykernel_2596\770145647.py:8: RuntimeWarning: Mean of empty slice
  df_Visit1['VBM'] = np.nanmean(pd.concat([df_Visit1_rep1['VBM'], df_Visit1_rep2['VBM']], axis=1), axis=1)
```

Out[23]:

|  | SubjectID | GroupID | HoneyW | Posner | CMot | VA | Stroop | Con | VBM |
| --- | --- | --- | --- | --- | --- | --- | --- | --- | --- |
| 0 | 10-101 | 10 | 69.515050 | 0.044424 | 25.815731 | 2.039291 | 0.105136 | 2.322605 | NaN |
| 1 | 10-102 | 10 | 123.237175 | -0.024719 | 6.807445 | 2.132655 | 0.001942 | 1.765289 | 18.000003 |
| 2 | 10-103 | 10 | NaN | -0.070768 | 12.222734 | 2.281451 | 0.007591 | 2.380323 | 18.000001 |
| 3 | 10-104 | 10 | 194.744650 | 0.075059 | 7.148880 | 1.604792 | -0.006244 | 1.724513 | 18.000001 |
| 4 | 10-105 | 10 | 78.861000 | 0.091197 | 3.941043 | 0.686221 | 0.007656 | 1.970616 | NaN |

## Quick view of raw data¶

### Normality assumption raw data¶

In [24]:

```
fig, axs = plt.subplots(2, 4, figsize=(30, 18), sharey=False, sharex=False)

for var, ax in zip(variables, axs.reshape(-1)):

    sb.histplot(data=df_Visit1.dropna(how = 'any', subset = [var]), x = var, hue= 'GroupID', palette=['orangered', 'steelblue'], alpha = 0.5, element="step", ax = ax)
    
    ax.set_title(var, fontsize = 40)
    ax.set_xlabel(None)
    ax.set_ylabel('Count', fontsize=35)
    ax.get_legend().remove()
    ax.tick_params(axis='both', which='major', labelsize=32)

plt.savefig('./Figures/2.Histograms_RawVar.jpg')
```

In [25]:

```
norm_Visit1 = pg.normality(df_Visit1[variables])
norm_Visit1.rename(columns = {'W':'W_test1', 'pval':'pval_test1'}, inplace=True)
norm_Visit1.round(3)
```

Out[25]:

|  | W\_test1 | pval\_test1 | normal |
| --- | --- | --- | --- |
| VA | 0.901 | 0.004 | False |
| Con | 0.931 | 0.031 | False |
| CMot | 0.771 | 0.000 | False |
| VBM | 0.619 | 0.000 | False |
| HoneyW | 0.849 | 0.000 | False |
| Stroop | 0.935 | 0.032 | False |
| Posner | 0.986 | 0.912 | True |

### Check missing values¶

In [26]:

```
AvailableData = pd.concat([df_Visit1.loc[(df_Visit1['GroupID']==10)].count(), df_Visit1.loc[(df_Visit1['GroupID']==90)].count()], axis=1).T
AvailableData.rename(index={0:"Visit1 patients", 1:"Visit1 controls"})
```

Out[26]:

|  | SubjectID | GroupID | HoneyW | Posner | CMot | VA | Stroop | Con | VBM |
| --- | --- | --- | --- | --- | --- | --- | --- | --- | --- |
| Visit1 patients | 20 | 20 | 16 | 20 | 19 | 19 | 20 | 19 | 16 |
| Visit1 controls | 17 | 17 | 17 | 17 | 17 | 17 | 17 | 16 | 15 |

In [27]:

```
for i in range(len(df_Visit1)):
    if df_Visit1.iloc[i, 2: ].isnull().sum() > 0:
        print("NaN for ", i, df_Visit1.iloc[i, 0] , " : " ,  df_Visit1.iloc[i, 2: ].isnull().sum())
```

```
NaN for  0 10-101  :  1
NaN for  2 10-103  :  1
NaN for  4 10-105  :  1
NaN for  5 10-106  :  1
NaN for  7 10-108  :  2
NaN for  8 10-109  :  1
NaN for  9 10-110  :  1
NaN for  14 10-115  :  1
NaN for  18 10-119  :  1
NaN for  19 10-120  :  1
NaN for  29 90-109  :  1
NaN for  31 90-111  :  1
NaN for  32 90-112  :  1
```

### Comparison controls-patients¶

In [28]:

```
def tTest_MWU(feature, df_1, df_2): # non-parametric test
    # Save: T, dof, p-val, cohen-d
    tt_results = pg.mwu(df_1[feature], df_2[feature], alternative='two-sided').round(3) 
    final_results = tt_results.loc[:,['U-val','p-val','CLES']].copy()
    final_results = final_results.rename(columns={'T':'T-test t'})
    final_results.insert(loc = 0, column = 'Variable:', value = feature)
    return final_results
```

In [29]:

```
#compute MWU: controls vs. patients

df_results = pd.DataFrame()

for var in variables:
    result = tTest_MWU(var, df_Visit1.loc[(df_Visit1['GroupID']==10)], df_Visit1.loc[(df_Visit1['GroupID']==90)])    
    df_results = pd.concat([df_results, result], axis = 0)
    
#correct p-values for multiple comparisons with BH (for study eye and other eye separately)
reject, pvals_corr = pg.multicomp(df_results.loc[:,'p-val'], alpha=0.05, method='holm')

# add pvals_corr to df_results
df_results['p-BH'] = pvals_corr.tolist()
df_results
```

Out[29]:

|  | Variable: | U-val | p-val | CLES | p-BH |
| --- | --- | --- | --- | --- | --- |
| MWU | VA | 203.0 | 0.194 | 0.628 | 0.970 |
| MWU | Con | 148.0 | 0.908 | 0.487 | 1.000 |
| MWU | CMot | 128.0 | 0.296 | 0.396 | 1.000 |
| MWU | VBM | 72.0 | 0.060 | 0.300 | 0.420 |
| MWU | HoneyW | 87.0 | 0.081 | 0.320 | 0.486 |
| MWU | Stroop | 205.0 | 0.293 | 0.603 | 1.000 |
| MWU | Posner | 153.0 | 0.615 | 0.450 | 1.000 |

In [30]:

```
def descriptive_stats_table(df, variables):
    
    demographics_table = pd.DataFrame()
    
    for j, group in enumerate(Group_IDs):    
        df_temp = df.loc[(df['GroupID'] == group), variables].copy()
        continuous_vars = df_temp.mean().round(3).astype(str) + ' ± ' + df_temp.std().round(3).astype(str)
        demographics_table[Group_labels[j]] = continuous_vars
            
    #We create multilevel column headers
    group_headers = [group for group in Group_labels]
    demographics_table.columns = [group_headers]
    
    #Replace all missing values by blank spaces:
    demographics_table = demographics_table.replace('nan ± nan','').replace('nan / nan','')
    
    return demographics_table
```

In [31]:

```
descriptive_stats_table(df_Visit1, variables)
```

Out[31]:

|  | Controls | Patients |
| --- | --- | --- |
| VA | 1.635 ± 0.422 | 1.829 ± 0.385 |
| Con | 2.016 ± 0.373 | 2.005 ± 0.345 |
| CMot | 16.951 ± 9.85 | 16.365 ± 16.227 |
| VBM | 34.961 ± 21.903 | 31.471 ± 30.079 |
| HoneyW | 220.918 ± 135.94 | 149.949 ± 98.417 |
| Stroop | 0.006 ± 0.055 | 0.023 ± 0.051 |
| Posner | 0.056 ± 0.038 | 0.047 ± 0.06 |

## Pre-process the data¶

### 1. Outlier removal¶

- Compute modified z-score and remove outliers (3.5 criterium)
- 3 dataframes for Visit1 after this step: df\_Visit1 = original one, df\_Visit1\_Zm = Zm-scores, df\_Visit1\_noOut = df\_Visit1 without outliers

In [32]:

```
# 1. Compute modified Zm 
df_Visit1_Zm = modified_z_score(df_Visit1, variables)
```

In [33]:

```
# See how many outliers
nbSubj_outliers_Visit1 = df_Visit1_Zm.loc[:,variables][np.abs(df_Visit1_Zm.loc[:,variables])>3.5].count(axis=0)
print(nbSubj_outliers_Visit1)
```

```
VA        0
Con       0
CMot      3
VBM       4
HoneyW    3
Stroop    0
Posner    0
dtype: int64
```

In [34]:

```
## See who is the outlier
if len(df_Visit1_Zm.loc[np.abs(df_Visit1_Zm['CMot']) > 3.5]) > 0:
    print("Subjects with outliers for "+'CMot_Visit1')
    display(df_Visit1.loc[np.abs(df_Visit1_Zm['CMot']) > 3.5])
```

```
Subjects with outliers for CMot_Visit1
```

|  | SubjectID | GroupID | HoneyW | Posner | CMot | VA | Stroop | Con | VBM |
| --- | --- | --- | --- | --- | --- | --- | --- | --- | --- |
| 7 | 10-108 | 10 | 189.82625 | 0.004653 | 45.332692 | 1.997259 | -0.010381 | NaN | NaN |
| 15 | 10-116 | 10 | 168.50055 | 0.027707 | 68.721965 | 1.992118 | -0.020440 | 1.879367 | 18.000001 |
| 36 | 90-116 | 90 | 302.13435 | 0.101298 | 42.348364 | 1.499119 | -0.019184 | 1.501018 | 18.099461 |

In [35]:

```
## See who is the outlier
if len(df_Visit1_Zm.loc[np.abs(df_Visit1_Zm['VBM']) > 3.5]) > 0:
    display(df_Visit1.loc[np.abs(df_Visit1_Zm['VBM']) > 3.5])
```

|  | SubjectID | GroupID | HoneyW | Posner | CMot | VA | Stroop | Con | VBM |
| --- | --- | --- | --- | --- | --- | --- | --- | --- | --- |
| 9 | 10-110 | 10 | 94.604075 | 0.004082 | NaN | 1.377834 | 0.121817 | 2.143014 | 133.880596 |
| 16 | 10-117 | 10 | 273.359250 | 0.151526 | 16.110786 | 1.445981 | 0.123676 | 2.741708 | 67.515036 |
| 27 | 90-107 | 90 | 301.912975 | 0.061024 | 22.363128 | 1.672976 | -0.045888 | 1.667325 | 99.679644 |
| 33 | 90-113 | 90 | 223.640325 | 0.080687 | 32.069484 | 1.526266 | 0.043523 | 2.074717 | 65.842126 |

In [36]:

```
## See who is the outlier
if len(df_Visit1_Zm.loc[np.abs(df_Visit1_Zm['HoneyW']) > 3.5]) > 0:
    display(df_Visit1.loc[np.abs(df_Visit1_Zm['HoneyW']) > 3.5])
```

|  | SubjectID | GroupID | HoneyW | Posner | CMot | VA | Stroop | Con | VBM |
| --- | --- | --- | --- | --- | --- | --- | --- | --- | --- |
| 10 | 10-111 | 10 | 443.02375 | 0.042764 | 14.382256 | 2.004251 | 0.023907 | 1.541988 | 18.000030 |
| 32 | 90-112 | 90 | 523.02640 | 0.010877 | 31.830562 | 0.681665 | 0.109132 | 3.074182 | NaN |
| 35 | 90-115 | 90 | 437.04035 | 0.001237 | 8.410354 | 1.988682 | -0.000960 | 1.665102 | 19.317992 |

In [37]:

```
# 2. Remove outliers from raw datasets
def outlier_removal(df, df_Zm, variables, return_Zm):
    
    if return_Zm:
        df_noOut = df_Zm.copy()
    else:
        df_noOut = df.copy()   
    
    for var in variables:
        df_noOut.loc[np.abs(df_Zm[var])>3.5, var] = np.nan

    return df_noOut
```

In [38]:

```
df_Visit1_noOut = outlier_removal(df_Visit1, df_Visit1_Zm, variables, False)
```

In [39]:

```
print(df_Visit1['CMot'].isna().sum())
print(df_Visit1_noOut['CMot'].isna().sum())
```

```
1
4
```

### 2. Yeo–Johnson power transformation¶

- 1. Find best lambda on Visit1 data without outliers (df\_Visit1\_noOut) to maximize normality
- 1. Transform Visit1 data (df\_Visit1) with best lamba found in 1

In [40]:

```
def Optimal_lambda(df, variables):
    
    lambdas_df = pd.DataFrame()
    ol = PowerTransformer(method='yeo-johnson', standardize=False, copy=True)
    
    for var in variables:
        lambdas_df[var] = ol.fit(df[var].values.reshape(-1,1)).lambdas_
    
    lambdas_df.rename(index={0: "Opt_lambda"}, inplace = True)
    
    return lambdas_df
```

In [41]:

```
lambdas_Visit1_df = Optimal_lambda(df_Visit1_noOut, variables)
print(lambdas_Visit1_df.round(3))
```

```
               VA    Con   CMot    VBM  HoneyW  Stroop  Posner
Opt_lambda  3.436 -2.451  0.055 -1.628  -0.176  -2.291   1.149
```

In [42]:

```
def PowerTransf(df, lambdas_df):
    
    variables = list(lambdas_df.columns)
    df_PT = df.copy()
    
    for var in variables:
        lambda_ = lambdas_df[var].values
        df_PT[var] = stats.yeojohnson(df[var], lmbda=lambda_)
    
    return df_PT
```

In [43]:

```
df_Visit1_PT = PowerTransf(df_Visit1, lambdas_Visit1_df)
```

### 3. Standardize (Zm) Power transformed data and remove outliers¶

In [44]:

```
df_Visit1_PT_Zm = modified_z_score(df_Visit1_PT, variables)
```

In [45]:

```
nbSubj_Outliers_Visit1_PT = df_Visit1_PT_Zm.loc[:,variables][np.abs(df_Visit1_PT_Zm.loc[:,variables])>3.5].count(axis=0)
print(nbSubj_Outliers_Visit1_PT)
```

```
VA        0
Con       0
CMot      0
VBM       0
HoneyW    0
Stroop    0
Posner    0
dtype: int64
```

In [46]:

```
# see who is the outlier
if len(df_Visit1_PT_Zm.loc[np.abs(df_Visit1_PT_Zm['CMot']) > 3.5]) > 0:
    print("Subjects with outliers for "+'CMot_Visit1')
    display(df_Visit1_PT_Zm.loc[np.abs(df_Visit1_PT_Zm['CMot']) > 3.5])
```

In [47]:

```
df_Visit1_PT_Zm_noOut = outlier_removal(df_Visit1_PT_Zm, df_Visit1_PT_Zm, variables, True) # final dataset
```

In [48]:

```
# just get easier name
df_Visit1_final = df_Visit1_PT_Zm_noOut.copy()
```

### Describe raw data without outliers¶

In [49]:

```
descriptive_stats_table(df_Visit1, variables)
```

Out[49]:

|  | Controls | Patients |
| --- | --- | --- |
| VA | 1.635 ± 0.422 | 1.829 ± 0.385 |
| Con | 2.016 ± 0.373 | 2.005 ± 0.345 |
| CMot | 16.951 ± 9.85 | 16.365 ± 16.227 |
| VBM | 34.961 ± 21.903 | 31.471 ± 30.079 |
| HoneyW | 220.918 ± 135.94 | 149.949 ± 98.417 |
| Stroop | 0.006 ± 0.055 | 0.023 ± 0.051 |
| Posner | 0.056 ± 0.038 | 0.047 ± 0.06 |

### 4. Switch sign of all but VA and HoneyW to have all high=better perf¶

In [50]:

```
df_Visit1_final.loc[:, ['Con', 'CMot', 'VBM', 'Stroop', 'Posner']] = -1*df_Visit1_final.loc[:, ['Con', 'CMot', 'VBM', 'Stroop', 'Posner']]
```

### 5. Check Floor and ceiling effects¶

In [51]:

```
def my_FloorCeiling(df, variable):
    result = pd.Series(df.loc[:, variable]).value_counts().values[0]/len(df.loc[:, variable])*100
    measure = pd.Series(df.loc[:, variable]).value_counts().index[0]
    df_FloorCeiling = pd.DataFrame(data=[result.round(2), measure], columns=[variable])
    df_FloorCeiling.rename(index={0:"FloorCeiling", 1:"Measure"}, inplace=True)
    return df_FloorCeiling
```

In [52]:

```
df_FloorCeiling = pd.DataFrame()

for var in variables:
    result = my_FloorCeiling(df_Visit1_final, var) 
    df_FloorCeiling = pd.concat([df_FloorCeiling, result], axis = 1)

df_FloorCeiling
```

Out[52]:

|  | VA | Con | CMot | VBM | HoneyW | Stroop | Posner |
| --- | --- | --- | --- | --- | --- | --- | --- |
| FloorCeiling | 2.700000 | 2.700000 | 2.700000 | 2.700000 | 2.700000 | 2.700000 | 2.7 |
| Measure | 0.813577 | -0.975219 | -1.180687 | 0.683168 | -1.140795 | -2.069289 | -0.0 |

## Normality assumption clean data¶

In [53]:

```
fig, axs = plt.subplots(2, 4, figsize=(30, 18), sharey=False, sharex=False)

for var, ax in zip(variables, axs.reshape(-1)):

    sb.histplot(data=df_Visit1_final.dropna(how = 'any', subset = [var]), x = var, hue= 'GroupID', palette=['orangered', 'steelblue'], alpha = 0.5, element="step", ax = ax)
    
    ax.set_title(var, fontsize = 40)
    ax.set_xlabel(None)
    ax.set_ylabel('Count', fontsize=35)
    ax.get_legend().remove()
    ax.tick_params(axis='both', which='major', labelsize=32)
    
plt.savefig('./Figures/3.Histograms_CleanVar.jpg')
```

In [54]:

```
norm_Visit1_clean = pg.normality(df_Visit1_final[variables])
norm_Visit1_clean.rename(columns = {'W':'W_test1', 'pval':'pval_test1'}, inplace=True)
norm_Visit1_clean.round(3)
```

Out[54]:

|  | W\_test1 | pval\_test1 | normal |
| --- | --- | --- | --- |
| VA | 0.953 | 0.132 | True |
| Con | 0.985 | 0.893 | True |
| CMot | 0.977 | 0.627 | True |
| VBM | 0.830 | 0.000 | False |
| HoneyW | 0.971 | 0.518 | True |
| Stroop | 0.951 | 0.103 | True |
| Posner | 0.986 | 0.912 | True |

In [55]:

```
## Missing data
df_Visit1_final[variables].isna().sum().sum()/((20+17)*7)*100
```

Out[55]:

```
5.405405405405405
```

## Controls vs. patients¶

In [56]:

```
def tTest(feature, df_1, df_2): #Student t-test
    # Save: T, dof, p-val, cohen-d
    tt_results = pg.ttest(df_1[feature], df_2[feature], correction=False).round(3) 
    final_results = tt_results.loc[:,['T','dof','p-val','cohen-d']].copy()
    final_results = final_results.rename(columns={'T':'T-test t'})
    final_results.insert(loc = 0, column = 'Variable:', value = feature)
    return final_results
```

In [57]:

```
#compute t-Tests: controls vs. patients

df_results = pd.DataFrame()

for var in variables:
    result = tTest(var, df_Visit1_final.loc[(df_Visit1_final['GroupID']==10)], df_Visit1_final.loc[(df_Visit1_final['GroupID']==90)])    
    df_results = pd.concat([df_results, result], axis = 0)

#correct p-values for multiple comparisons with BH (for study eye and other eye separately)
reject, pvals_corr = pg.multicomp(df_results.loc[:, 'p-val'], alpha=0.05, method='holm')

# add pvals_corr to df_results
df_results['p-BH'] = pvals_corr
df_results
```

Out[57]:

|  | Variable: | T-test t | dof | p-val | cohen-d | p-BH |
| --- | --- | --- | --- | --- | --- | --- |
| T-test | VA | 1.544 | 34 | 0.132 | 0.516 | 0.756 |
| T-test | Con | 0.080 | 33 | 0.937 | 0.027 | 1.000 |
| T-test | CMot | 0.960 | 34 | 0.344 | 0.321 | 1.000 |
| T-test | VBM | 1.577 | 29 | 0.126 | 0.567 | 0.756 |
| T-test | HoneyW | -1.837 | 31 | 0.076 | 0.640 | 0.532 |
| T-test | Stroop | -1.047 | 35 | 0.302 | 0.346 | 1.000 |
| T-test | Posner | 0.518 | 35 | 0.607 | 0.171 | 1.000 |

In [58]:

```
def group_comparison(df, features): #barplots but can change kind to have boxplots, violinplots....

    df_temp = df.loc[:, features+['GroupID']].copy()
    df_temp = pd.melt(df_temp, id_vars = ['GroupID'], var_name = 'variable', value_name = 'value') 
    
    #g = sb.catplot(data=df_temp, kind="box", x="variable", y="value", hue="GroupID", palette=['orangered', 'steelblue'], height = 6, aspect = 2, errorbar='se')
    g = sb.catplot(data=df_temp, kind="bar", x="variable", y="value", hue="GroupID", palette=['orangered', 'steelblue'], height = 6, aspect = 2, errorbar='se')
    
    #kind="bar"
    
    g.set_axis_labels("", "Standardized score", fontsize = 18)
    g.despine(left=True)
    g.legend.set_title("")
    #g.set(ylim=(-0.8, 0.8))
    g.set_xticklabels(fontsize = 18)
    g.set_yticklabels(fontsize = 17)
```

In [59]:

```
group_comparison(df_Visit1_final, variables) 

#plt.savefig('./Figures/4.Pointsplot_CtrVsPat.jpg') 
plt.savefig('./Figures/4.Boxplot_CtrVsPat.jpg')
```

## Between-variables correlations¶

In [60]:

```
def p_val_matrix(df, method = 'spearman'):
    #Create a p-values matrix corresponding to the correlation matrix we would obtain for df
    
    p_matrix = np.zeros(shape=(df.shape[1], df.shape[1]))
    
    for col in df.columns:
        for col2 in df.drop(col,axis=1).columns:
            p = pg.corr(df[col], df[col2], method = method).loc[method,'p-val']
            p_matrix[df.columns.to_list().index(col), df.columns.to_list().index(col2)] = p
    
    return p_matrix
```

In [61]:

```
def corStat(df, variables):
    
    cor_stat = pd.DataFrame()
    
    # % of significant corr
    nbCor = len(variables)*(len(variables)-1)/2
    cor_stat['pSig'] = [sum(sum(np.tril(p_val_matrix(df[variables])<0.05, -1)))/nbCor*100] #-1 not to include diagonal
    
    # Percentiles (25th/50th/75th)
    corr = df[variables].corr(method = 'spearman')
    corr.values[np.triu_indices_from(corr, 0)] = np.nan #get only lower tri without diagonal
    cor_stat['0.25'] = [np.nanpercentile(corr, 25)]
    cor_stat['0.50'] = [np.nanpercentile(corr, 50)]
    cor_stat['0.75'] = [np.nanpercentile(corr, 75)]
    
    # Var explained by 1st eigenvalue (PCA)
    eigen_vals, eigen_vecs = np.linalg.eig(df[variables].corr(method = 'spearman'))
    cor_stat['VarExp_1V'] = [eigen_vals[0]/sum(eigen_vals)*100]
    
    return cor_stat.round(3)
```

In [62]:

```
df_all = df_Visit1_final[variables]
df_P = df_Visit1_final.loc[(df_Visit1_final['GroupID']==10), variables]
df_C = df_Visit1_final.loc[(df_Visit1_final['GroupID']==90), variables]
```

### Groups merged¶

In [63]:

```
plt.figure(figsize=(7, 7))

#Define mask (bool matrix) that will allow us to put significant p-values in bold 
mask_bold = np.invert(np.tril(p_val_matrix(df_all)<0.05)) 
np.fill_diagonal(mask_bold,'True') 
mask = np.triu(df_all.corr()) - np.invert(mask_bold)

#We benefit from the fact that each plotting is superimposed until the next "plt.show()"  
#and successively plot the lower triangle values and the bold lowerTri values
g = sb.heatmap(df_all.corr(method = 'pearson').round(2), cmap="PiYG", cbar_kws={'label': "Pearson's correlation","shrink": 0.35},annot_kws={"fontsize":15}, vmin=-1, vmax=1, annot = True,square = True, mask = mask,linewidths=2.5)
sb.heatmap(df_all.corr(method = 'pearson').round(2), cmap="PiYG", cbar = False, annot_kws={"fontsize":15, "weight": "bold"}, vmin=-1, vmax=1, annot = True,square = True, mask = mask_bold,linewidths=2.5)

#we increase the fontsize of:
cbar = g.collections[0].colorbar
cbar.ax.tick_params(labelsize=12) #the numbers of the colorbar
g.figure.axes[-1].yaxis.label.set_size(20) #the label of the colorbar
g.set_xticklabels(g.get_xmajorticklabels(), fontsize = 12) #the correlation matrix xlabels
g.set_yticklabels(g.get_ymajorticklabels(), fontsize = 12) #and ylabels
```

Out[63]:

```
[Text(0, 0.5, 'VA'),
 Text(0, 1.5, 'Con'),
 Text(0, 2.5, 'CMot'),
 Text(0, 3.5, 'VBM'),
 Text(0, 4.5, 'HoneyW'),
 Text(0, 5.5, 'Stroop'),
 Text(0, 6.5, 'Posner')]
```

### Groups seperated¶

In [64]:

```
## Plot variables corr for patients (lower triangle) and controls (upper triangle)
plt.figure(figsize=(7, 7))

#Define mask (bool matrix) that will allow us to put significant p-values in bold 
mask_bold_P = np.invert(np.tril(p_val_matrix(df_P)<0.05)) #tril refers to the lower triangle matrix
np.fill_diagonal(mask_bold_P,'True') #We don't want to highlight the diagonal
mask_P = np.triu(df_P.corr()) - np.invert(mask_bold_P)

mask_bold_C = np.invert(np.triu(p_val_matrix(df_C)<0.05)) #triu refers to the upper triangle matrix
np.fill_diagonal(mask_bold_C,'True') 
mask_C = np.tril(df_C.corr()) - np.invert(mask_bold_C)

#We benefit from the fact that each plotting is superimposed until the next "plt.show()"  
#and successively plot the lower triangle values (controls), the bold lowerTri values, the upper triangle values (patients) and bold upperTri values
g = sb.heatmap(df_P.corr(method = 'spearman').round(2), cmap="PiYG", cbar_kws={'label': "Spearman's correlation","shrink": 0.35},annot_kws={"fontsize":15}, vmin=-1, vmax=1, annot = True,square = True, mask = mask_P,linewidths=2.5)
sb.heatmap(df_P.corr(method = 'spearman').round(2), cmap="PiYG", cbar = False, annot_kws={"fontsize":15, "weight": "bold"}, vmin=-1, vmax=1, annot = True,square = True, mask = mask_bold_P,linewidths=2.5)
sb.heatmap(df_C.corr(method = 'spearman').round(2), cmap="PiYG", cbar = False, annot_kws={"fontsize":15}, vmin=-1, vmax=1, annot = True,square = True, mask = mask_C,linewidths=2.5)
sb.heatmap(df_C.corr(method = 'spearman').round(2), cmap="PiYG", cbar = False, annot_kws={"fontsize":15, "weight": "bold"}, vmin=-1, vmax=1, annot = True,square = True, mask = mask_bold_C,linewidths=2.5)

#we increase the fontsize of:
cbar = g.collections[0].colorbar
cbar.ax.tick_params(labelsize=12) #the numbers of the colorbar
g.figure.axes[-1].yaxis.label.set_size(20) #the label of the colorbar
g.set_xticklabels(g.get_xmajorticklabels(), fontsize = 12) #the correlation matrix xlabels
g.set_yticklabels(g.get_ymajorticklabels(), fontsize = 12) #and ylabels

plt.savefig('./Figures/5.SpearmanCorr_Psychophysics_PatDown_CtrlUp.jpg')
```

In [65]:

```
cor_stat_all = corStat(df_all, variables)
cor_stat_P = corStat(df_P, variables)
cor_stat_C = corStat(df_C, variables)
pd.concat([cor_stat_all, cor_stat_P, cor_stat_C], axis = 0, keys=['all_Visit1','patients_Visit1', 'controls_Visit1'])
```

Out[65]:

|  |  | pSig | 0.25 | 0.50 | 0.75 | VarExp\_1V |
| --- | --- | --- | --- | --- | --- | --- |
| all\_Visit1 | 0 | 9.524 | -0.012 | 0.139 | 0.232 | 27.339 |
| patients\_Visit1 | 0 | 0.000 | -0.075 | 0.140 | 0.329 | 28.698 |
| controls\_Visit1 | 0 | 0.000 | -0.032 | 0.075 | 0.212 | 1.812 |

### Compare the pearson's corr matrices controls vs. patients¶

In [66]:

```
# Compare the pearson's corr matrices controls vs. patients

def independent_corr_comparison(xy, ab, n, n2 = None, twotailed=True, conf_level=0.95):
    #copied by: https://github.com/psinger/CorrelationStats/blob/master/corrstats.py
    # check by what I used to do with R and looks the same: (R: To make the correlation coefficients comparable across groups, i.e., to account for the differences in the sample sizes, 
# use the Fisher's r-to-Z transformation Z =  1/2*(ln(1+r)-ln(1-r)) (Fisher, 1921) and compare the z test statistics across two groups as: 
# Z=  (z_1-z_2)/squrt((1/(n_1-3)+ 1/(n_2-3))) where z1 and z2 are the two transformed correlations to compare and n1 and n2 are the sample sizes of the two groups 
## We can use cocor.indep.groups fct of cocor library)
    
    #Calculates the statistic significance between two independent correlation coefficients
    #param xy: correlation coefficient between x and y
    #param xz: correlation coefficient between a and b
    #param n: number of elements in xy
    #param n2: number of elements in ab (if distinct from n)
    #param twotailed: whether to calculate a one or two tailed test, only works for 'fisher' method
    #param conf_level: confidence level, only works for 'zou' method
    #param method: defines the method uses, 'fisher' or 'zou'
    #return: z and p-val

    xy_z = 0.5 * np.log((1 + xy)/(1 - xy))
    ab_z = 0.5 * np.log((1 + ab)/(1 - ab))
    if n2 is None:
        n2 = n

    se_diff_r = np.sqrt(1/(n - 3) + 1/(n2 - 3))
    diff = xy_z - ab_z
    #z = abs(diff / se_diff_r)
    z = diff / se_diff_r #SG changed so that I can see which one is larger
    z_p = abs(diff / se_diff_r)
    p = (1 - stats.norm.cdf(z_p))
    if twotailed:
        p *= 2

    return z, p


def independent_cor_df(cor1_df, cor2_df, nbC, nbP):
    
    indep_cor_df_p = pd.DataFrame()
    indep_cor_df_z = pd.DataFrame()
    #indep_cor_matrix_p = np.zeros(shape=(cor1_df.shape[1], cor1_df.shape[1]))
    #indep_cor_matrix_z = np.zeros(shape=(cor1_df.shape[1], cor1_df.shape[1]))
    
    for col in cor1_df.columns:
        for col2 in cor1_df.drop(col,axis=1).columns:
            indep_cor_z, indep_cor_p = independent_corr_comparison(cor1_df.loc[col, col2], cor2_df.loc[col, col2], nbC, nbP)
            indep_cor_df_z.loc[col, col2] = indep_cor_z
            indep_cor_df_p.loc[col, col2] = indep_cor_p
            #indep_cor_matrix_z[cor1_df.columns.to_list().index(col), cor1_df.columns.to_list().index(col2)] = indep_cor_z
            #indep_cor_matrix_p[cor1_df.columns.to_list().index(col), cor1_df.columns.to_list().index(col2)] = indep_cor_p
        
    return indep_cor_df_z, indep_cor_df_p
```

In [67]:

```
cor_df_comparison_z, cor_df_comparison_p = independent_cor_df(df_C.corr(method = 'spearman'), df_P.corr(method = 'spearman'), 17, 18)
```

In [68]:

```
cor_df_comparison_p
```

Out[68]:

|  | Con | CMot | VBM | HoneyW | Stroop | Posner | VA |
| --- | --- | --- | --- | --- | --- | --- | --- |
| VA | 0.972093 | 0.116299 | 0.452427 | 0.342330 | 0.974532 | 0.303287 | NaN |
| Con | NaN | 0.723863 | 0.721939 | 0.424338 | 0.193921 | 0.259197 | 0.972093 |
| CMot | 0.723863 | NaN | 0.598068 | 0.830461 | 0.739114 | 0.359502 | 0.116299 |
| VBM | 0.721939 | 0.598068 | NaN | 0.425681 | 0.126518 | 0.837953 | 0.452427 |
| HoneyW | 0.424338 | 0.830461 | 0.425681 | NaN | 0.925106 | 0.322870 | 0.342330 |
| Stroop | 0.193921 | 0.739114 | 0.126518 | 0.925106 | NaN | 0.808557 | 0.974532 |
| Posner | 0.259197 | 0.359502 | 0.837953 | 0.322870 | 0.808557 | NaN | 0.303287 |

In [69]:

```
cor_matrix_comparison_p = cor_df_comparison_p.iloc[:,[6,0,1,2,3,4,5]].values
cor_df_comparison_z_plot = cor_df_comparison_z.iloc[:,[6,0,1,2,3,4,5]]
```

In [70]:

```
plt.figure(figsize=(7, 7))

mask_bold_z = np.invert(np.tril(cor_matrix_comparison_p<0.05)) 
np.fill_diagonal(mask_bold_z,'True') 
mask_z = np.triu(cor_df_comparison_z_plot) - np.invert(mask_bold_z)

g = sb.heatmap(cor_df_comparison_z_plot.round(2), cmap="PiYG", cbar_kws={'label': "Z-test statistic","shrink": 0.35},annot_kws={"fontsize":15}, vmin=-3.5, vmax=3.5, annot = True,square = True, mask = mask_z,linewidths=2.5)
sb.heatmap(cor_df_comparison_z_plot.round(2), cmap="PiYG", cbar = False, annot_kws={"fontsize":15, "weight": "bold"}, vmin=-1, vmax=1, annot = True,square = True, mask = mask_bold_z,linewidths=2.5)

cbar = g.collections[0].colorbar
cbar.ax.tick_params(labelsize=12) #the numbers of the colorbar
g.figure.axes[-1].yaxis.label.set_size(20) #the label of the colorbar
g.set_xticklabels(g.get_xmajorticklabels(), fontsize = 12) #the correlation matrix xlabels
g.set_yticklabels(g.get_ymajorticklabels(), fontsize = 12) #and ylabels
```

Out[70]:

```
[Text(0, 0.5, 'VA'),
 Text(0, 1.5, 'Con'),
 Text(0, 2.5, 'CMot'),
 Text(0, 3.5, 'VBM'),
 Text(0, 4.5, 'HoneyW'),
 Text(0, 5.5, 'Stroop'),
 Text(0, 6.5, 'Posner')]
```

In [71]:

```
# Var explained by eigenvalues (PCA) 
eigen_vals_all, eigen_vecs_all = np.linalg.eig(df_all.corr(method = 'spearman'))
eigen_vals_P, eigen_vecs_P = np.linalg.eig(df_P.corr(method = 'spearman'))
eigen_vals_C, eigen_vecs_C = np.linalg.eig(df_C.corr(method = 'spearman'))

var_expl = pd.DataFrame(columns=['All_Visit1','Patients_Visit1', 'Controls_Visit1'], index=range(len(eigen_vals_P)))

eigen_vals_all.sort()
eigen_vals_P.sort()
eigen_vals_C.sort()

for i in range (len(eigen_vals_P)):
    var_expl.iloc[6-i, 0] = eigen_vals_all[i]/sum(eigen_vals_all)*100
    var_expl.iloc[6-i, 1] = eigen_vals_P[i]/sum(eigen_vals_P)*100
    var_expl.iloc[6-i, 2] = eigen_vals_C[i]/sum(eigen_vals_C)*100

var_expl
```

Out[71]:

|  | All\_Visit1 | Patients\_Visit1 | Controls\_Visit1 |
| --- | --- | --- | --- |
| 0 | 27.338905 | 28.698136 | 27.057499 |
| 1 | 22.460219 | 20.69527 | 23.572938 |
| 2 | 16.48872 | 18.359739 | 19.829791 |
| 3 | 15.053209 | 15.149948 | 15.127995 |
| 4 | 8.940902 | 10.080619 | 8.009991 |
| 5 | 6.262319 | 6.013857 | 4.589782 |
| 6 | 3.455726 | 1.002431 | 1.812005 |

## Save clean Psychophysical data¶

In [72]:

```
#excel_writer = pd.ExcelWriter("./1.Visit1_clean.xlsx", engine='xlsxwriter')
#df_Visit1_final.to_excel(excel_writer, sheet_name='Visit1')
#excel_writer.save()
```

# Symptom's data¶

In [73]:

```
df_Symptoms = pd.read_excel('./0.ZurichSymptoms_Visit1.xlsx', sheet_name='Visit1', header = 0)
```

In [74]:

```
symptom_var_continuous = ['VS_ys_duration', 'Severity of VS (VAS)', 'Palinopsia', 'BlueField_EntopticPhenomena', 'Spontaneous photopsia', 'Floaters', 'Flashes in darkness', 'Photophobia', 'Nyctalopia', \
                          'Density', 'Speed', 'SurfaceDependence', 'Distraction', 'TimeCourse', 'Size', 'Outdoor_SunnyDay', 'Outdoor_CloudyDay', 'Outdoor_RainyDay', 'Indoor', 'FluorescentLighting', 'Outdoor_NightTime'] 

symptom_var_binary = ['Tinnitus', 'Migraine', 'Migraine with aura'] #comorbidities
```

In [75]:

```
## Add a column "ColorID": 1 = bw / 2=anything else
for i, row in df_Symptoms.iterrows():
    if row['Color'] == 1:
        df_Symptoms.loc[i, 'ColorID'] = 1
    else:
        df_Symptoms.loc[i, 'ColorID'] = 2
```

In [76]:

```
df_Symptoms.head()
```

Out[76]:

|  | GroupID | SubjectID | Tinnitus | Migraine | Migraine with aura | VS\_ys\_duration | Severity of VS (VAS) | Palinopsia | BlueField\_EntopticPhenomena | Spontaneous photopsia | Floaters | Flashes in darkness | Photophobia | Nyctalopia | Outdoor\_SunnyDay | Outdoor\_CloudyDay | Outdoor\_RainyDay | Indoor | FluorescentLighting | Outdoor\_NightTime | Density | Speed | SurfaceDependence | Distraction | TimeCourse | Color | Size | ColorID |
| --- | --- | --- | --- | --- | --- | --- | --- | --- | --- | --- | --- | --- | --- | --- | --- | --- | --- | --- | --- | --- | --- | --- | --- | --- | --- | --- | --- | --- |
| 0 | 10 | 10-101 | 1 | 0 | NaN | 3.0 | 7.5 | 0.0 | 6.5 | 4.5 | 7.5 | 6.5 | 4.0 | 0.0 | 3.0 | 3.0 | 2.0 | 5.0 | NaN | NaN | 4.23 | 2.87 | 2.57 | 3.63 | 4.00 | 3 | 5.00 | 2.0 |
| 1 | 10 | 10-102 | 0 | 1 | 1.0 | 1.0 | 4.0 | 0.0 | 7.0 | 2.0 | 7.0 | 5.0 | 7.0 | 3.0 | 6.0 | 5.0 | 3.0 | 4.0 | NaN | NaN | 2.27 | 4.00 | 2.43 | 2.33 | 2.17 | 2 | 1.97 | 2.0 |
| 2 | 10 | 10-103 | 1 | 0 | NaN | 2.0 | 6.0 | 1.0 | 6.0 | 0.0 | 9.0 | 4.0 | 5.0 | 4.0 | 3.0 | 5.0 | 6.0 | 4.0 | 2.0 | 1.0 | 3.03 | 3.10 | 3.30 | 2.53 | 4.00 | 3 | 2.00 | 2.0 |
| 3 | 10 | 10-104 | 0 | 0 | NaN | 2.0 | 4.0 | 1.5 | 7.0 | 1.5 | 6.0 | 2.0 | 6.5 | 3.0 | 4.0 | 1.0 | 7.0 | 5.0 | 3.0 | 4.0 | 2.53 | 2.87 | 2.83 | 2.30 | 1.70 | 6 | 2.90 | 2.0 |
| 4 | 10 | 10-105 | 0 | 1 | 0.0 | 2.0 | 6.0 | 0.0 | 0.0 | 3.0 | 4.5 | 4.0 | 4.0 | 3.5 | 3.0 | 4.0 | 7.0 | 2.0 | 2.0 | 1.0 | 2.60 | 2.93 | 2.37 | 2.30 | 1.70 | 1 | 2.90 | 1.0 |

## Descriptive table¶

In [77]:

```
def descriptive_symptom_table(df, variables_continuous, variables_binary, dict_row_names = None):
    
    _table = pd.DataFrame()
     
    #continuous variables (mean +- std)
    df_temp_cont = df[variables_continuous].copy()
    continuous_vars = df_temp_cont.mean().round(3).astype(str) + ' ± ' + df_temp_cont.std().round(3).astype(str)
        
    #binary variables (counts)
    df_temp_bin = df[variables_binary].copy()
    binary_vars = (df_temp_bin[variables_binary]==0).sum(axis=0).astype(str) + ' / ' + (df_temp_bin[variables_binary]==1).sum(axis=0).astype(str)          
          
    _table = pd.concat([continuous_vars, binary_vars], axis=0) 
            
    #We can change the names of some of the rows using a dictionary
    if dict_row_names != None:
        _table = _table.rename(dict_row_names)
    
    #display(demographics_table)
    return _table
```

In [78]:

```
# dict_raw_names
dict_row_names={'Tinnitus':'Tinnitus(n/y)', 'Migraine':'Migraine(n/y)','Migraine with aura':'Migraine with aura(n/y)'}
table_dem = descriptive_symptom_table(df_Symptoms, symptom_var_continuous, symptom_var_binary, dict_row_names)
```

In [79]:

```
## add colorID
table_dem.loc['Color (bw/others)'] = (df_Symptoms['ColorID']==1).sum(axis=0).astype(str) + ' / ' + (df_Symptoms['ColorID']==2).sum(axis=0).astype(str)   
table_dem
```

Out[79]:

```
VS_ys_duration                 6.525 ± 8.424
Severity of VS (VAS)            4.64 ± 1.523
Palinopsia                      2.92 ± 2.616
BlueField_EntopticPhenomena     4.025 ± 2.94
Spontaneous photopsia            2.75 ± 2.98
Floaters                        5.125 ± 2.68
Flashes in darkness            3.925 ± 2.556
Photophobia                      4.4 ± 3.255
Nyctalopia                     2.658 ± 2.438
Density                         2.73 ± 0.903
Speed                           3.07 ± 0.777
SurfaceDependence                2.9 ± 0.782
Distraction                    2.659 ± 0.661
TimeCourse                     2.814 ± 1.055
Size                           2.379 ± 1.187
Outdoor_SunnyDay               5.111 ± 1.779
Outdoor_CloudyDay              3.667 ± 1.847
Outdoor_RainyDay               4.222 ± 1.517
Indoor                         3.556 ± 1.464
FluorescentLighting            2.562 ± 1.413
Outdoor_NightTime                2.5 ± 1.897
Tinnitus(n/y)                         4 / 16
Migraine(n/y)                         11 / 9
Migraine with aura(n/y)                2 / 7
Color (bw/others)                     8 / 12
dtype: object
```

## Check normality assumption continuous variables¶

In [80]:

```
fig, axs = plt.subplots(3, 7, figsize=(70, 40), sharey=False, sharex=False)

for var, ax in zip(symptom_var_continuous, axs.reshape(-1)):

    sb.histplot(data=df_Symptoms[[var]].dropna(how = 'any', subset = [var]), x = var, color = 'orangered', alpha = 0.5, element="step", ax = ax)
    
    ax.set_title(var, fontsize = 40)
    ax.set_xlabel(None)
    ax.set_ylabel('Count', fontsize=35)
    ax.tick_params(axis='both', which='major', labelsize=32)

plt.savefig('./Figures/6.Histograms_RawDemog.jpg')
```

In [81]:

```
norm_symptoms = pg.normality(df_Symptoms.loc[:, symptom_var_continuous])
norm_symptoms.rename(columns = {'W':'W_test1', 'pval':'pval_test1'}, inplace=True)
norm_symptoms.round(3)
```

Out[81]:

|  | W\_test1 | pval\_test1 | normal |
| --- | --- | --- | --- |
| VS\_ys\_duration | 0.661 | 0.000 | False |
| Severity of VS (VAS) | 0.956 | 0.470 | True |
| Palinopsia | 0.907 | 0.057 | True |
| BlueField\_EntopticPhenomena | 0.918 | 0.092 | True |
| Spontaneous photopsia | 0.836 | 0.003 | False |
| Floaters | 0.983 | 0.969 | True |
| Flashes in darkness | 0.942 | 0.260 | True |
| Photophobia | 0.917 | 0.087 | True |
| Nyctalopia | 0.878 | 0.020 | False |
| Density | 0.961 | 0.564 | True |
| Speed | 0.901 | 0.043 | False |
| SurfaceDependence | 0.936 | 0.203 | True |
| Distraction | 0.931 | 0.159 | True |
| TimeCourse | 0.879 | 0.017 | False |
| Size | 0.867 | 0.010 | False |
| Outdoor\_SunnyDay | 0.893 | 0.043 | False |
| Outdoor\_CloudyDay | 0.896 | 0.049 | False |
| Outdoor\_RainyDay | 0.925 | 0.159 | True |
| Indoor | 0.939 | 0.282 | True |
| FluorescentLighting | 0.884 | 0.045 | False |
| Outdoor\_NightTime | 0.799 | 0.003 | False |

## Between-symptoms Correlations¶

In [82]:

```
df_Corr_Sympt = df_Symptoms.loc[:, symptom_var_continuous]
```

In [83]:

```
## Patients spearman's corr

## Plot variables corr for controls (lower triangle) and patients (upper triangle)
plt.figure(figsize=(21, 21))

#Define mask (bool matrix) that will allow us to put significant p-values in bold 
mask_bold = np.invert(np.tril(p_val_matrix(df_Corr_Sympt, method = 'spearman')<0.05)) #tril refers to the lower triangle matrix
np.fill_diagonal(mask_bold,'True') #We don't want to highlight the diagonal
mask_ = np.triu(df_Corr_Sympt.corr(method = 'spearman')) - np.invert(mask_bold)

#We benefit from the fact that each plotting is superimposed until the next "plt.show()"  
#and successively plot the lower triangle values (controls), the bold lowerTri values, the upper triangle values (patients) and bold upperTri values
g = sb.heatmap(df_Corr_Sympt.corr(method = 'spearman').round(2), cmap="PiYG", cbar_kws={'label': "Spearman's correlation","shrink": 0.35},annot_kws={"fontsize":15}, vmin=-1, vmax=1, annot = True,square = True, mask = mask_,linewidths=2.5)
sb.heatmap(df_Corr_Sympt.corr(method = 'spearman').round(2), cmap="PiYG", cbar = False, annot_kws={"fontsize":15, "weight": "bold"}, vmin=-1, vmax=1, annot = True,square = True, mask = mask_bold,linewidths=2.5)

#we increase the fontsize of:
cbar = g.collections[0].colorbar
cbar.ax.tick_params(labelsize=15) #the numbers of the colorbar
g.figure.axes[-1].yaxis.label.set_size(22) #the label of the colorbar
g.set_xticklabels(g.get_xmajorticklabels(), fontsize = 18) #the correlation matrix xlabels
g.set_yticklabels(g.get_ymajorticklabels(), fontsize = 18) #and ylabels

plt.savefig('./Figures/7.SpearmanCorr_Symptoms_Pat.jpg')
```

In [84]:

```
def corStatAbs(df, variables):
    
    cor_stat = pd.DataFrame()
    
    # % of significant corr
    nbCor = len(variables)*(len(variables)-1)/2
    cor_stat['pSig'] = [sum(sum(np.tril(p_val_matrix(df[variables])<0.05, -1)))/nbCor*100] #-1 not to include diagonal
    
    # Percentiles (25th/50th/75th)
    corr = df[variables].corr(method = 'spearman')
    corr.values[np.triu_indices_from(corr, 0)] = np.nan #get only lower tri without diagonal
    cor_stat['0.25'] = [np.nanpercentile(abs(corr), 25)]
    cor_stat['0.50'] = [np.nanpercentile(abs(corr), 50)]
    cor_stat['0.75'] = [np.nanpercentile(abs(corr), 75)]
    
    # Var explained by 1st eigenvalue (PCA)
    eigen_vals, eigen_vecs = np.linalg.eig(df[variables].corr(method = 'spearman'))
    cor_stat['VarExp_1V'] = [eigen_vals[0]/sum(eigen_vals)*100]
    
    return cor_stat.round(3)
```

In [85]:

```
corStatAbs(df_Corr_Sympt, symptom_var_continuous)
```

Out[85]:

|  | pSig | 0.25 | 0.50 | 0.75 | VarExp\_1V |
| --- | --- | --- | --- | --- | --- |
| 0 | 15.238 | 0.087 | 0.202 | 0.367 | 27.984 |

In [86]:

```
corStat(df_Corr_Sympt, symptom_var_continuous)
```

Out[86]:

|  | pSig | 0.25 | 0.50 | 0.75 | VarExp\_1V |
| --- | --- | --- | --- | --- | --- |
| 0 | 15.238 | -0.086 | 0.082 | 0.29 | 27.984 |

# Demographical data¶

In [87]:

```
df_Demographical = pd.read_excel('./0.ZurichDemographics.xlsx', sheet_name='Participants_visit1', header = 0) 
demog_var_continuous = ['Age'] 
demog_var_binary = ['Gender', 'Handedness']
```

In [88]:

```
df_Demographical.replace({'Gender': {'f': 0, 'm':1}}, regex=True, inplace=True)
df_Demographical.replace({'Handedness': {'right': 0, 'left':1}}, regex=True, inplace=True)
df_Demographical.replace({'Education': {'Matura': 1, 'Bachelor': 2, 'Master':3, 'PhD':4}}, regex=True, inplace=True)
```

In [89]:

```
df_Demographical.head()
```

Out[89]:

|  | GroupID | SubjectID | Age | Gender | Education | Handedness |
| --- | --- | --- | --- | --- | --- | --- |
| 0 | 10 | 10-101 | 26 | 1 | 2.0 | 0.0 |
| 1 | 10 | 10-102 | 31 | 0 | 3.0 | 0.0 |
| 2 | 10 | 10-103 | 25 | 1 | 2.0 | 0.0 |
| 3 | 10 | 10-104 | 28 | 0 | 2.0 | 1.0 |
| 4 | 10 | 10-105 | 35 | 1 | 4.0 | 0.0 |

## Descriptive tables¶

In [90]:

```
def descriptive_demog_table(df, variables_continuous, variables_binary, dict_row_names = None):
    
    demographics_table = pd.DataFrame()
    
    for j, group in enumerate(Group_IDs):    
        
        #continuous variables (mean +- std)
        df_temp_cont = df.loc[(df['GroupID'] == group), variables_continuous].copy()
        continuous_vars = df_temp_cont.mean().round(3).astype(str) + ' ± ' + df_temp_cont.std().round(3).astype(str)
        
        #binary variables (counts)
        df_temp_bin = df.loc[(df['GroupID'] == group), variables_binary].copy()
        binary_vars = (df_temp_bin[variables_binary]==0).sum(axis=0).astype(str) + ' / ' + (df_temp_bin[variables_binary]==1).sum(axis=0).astype(str)          
        
        #useful to add a sample size row
        N = pd.Series(len(df.loc[df['GroupID'] == group]), index=['N'])
        
        column = pd.concat([N, continuous_vars, binary_vars], axis=0) 
        demographics_table[Group_labels[j]] = column
            
    #We can change the names of some of the rows using a dictionary
    if dict_row_names != None:
        demographics_table = demographics_table.rename(dict_row_names)
    
    #We create multilevel column headers
    group_headers = [group for group in Group_labels]
    demographics_table.columns = [group_headers]
    
    #Replace all missing values by blank spaces:
    demographics_table = demographics_table.replace('nan ± nan','').replace('nan / nan','')
    demographics_table = demographics_table.replace('0 / 0','').replace('0 / 0','')
    
    #display(demographics_table)
    return demographics_table
```

In [91]:

```
# dict_raw_names
dict_row_names={'Gender':'Gender (F/M)', 'Handedness':'Handedness (R/L)'}
table_demog = descriptive_demog_table(df_Demographical, demog_var_continuous, demog_var_binary, dict_row_names)
```

In [92]:

```
for j, group in enumerate(Group_IDs):
    df_temp = df_Demographical.loc[(df_Demographical['GroupID'] == group), ['Education']].copy()
    table_demog.loc['Education (matura/bachelor/master/phd)', Group_labels[j]] = (df_temp['Education']==1).sum(axis=0).astype(str) + ' / ' + (df_temp['Education']==2).sum(axis=0).astype(str) + ' / ' + (df_temp['Education']==3).sum(axis=0).astype(str) + ' / ' + (df_temp['Education']==4).sum(axis=0).astype(str)

display(table_demog)
```

|  | Controls | Patients |
| --- | --- | --- |
| N | 17 | 20 |
| Age | 26.0 ± 5.339 | 31.55 ± 6.549 |
| Gender (F/M) | 12 / 5 | 7 / 13 |
| Handedness (R/L) | 11 / 0 | 16 / 4 |
| Education (matura/bachelor/master/phd) | 7 / 3 / 1 / 0 | 3 / 7 / 9 / 1 |

## Check normality assumption continuous variables¶

In [93]:

```
fig, axs = plt.subplots(1, 2, figsize=(10, 5), sharey=False, sharex=False)

for var, ax in zip(demog_var_continuous, axs.reshape(-1)):

    sb.histplot(data=df_Demographical.loc[(df_Demographical['GroupID']==10), [var]].dropna(how = 'any', subset = [var]), x = var, alpha = 0.5, element="step", ax = ax)
    
    ax.set_title(var, fontsize = 40)
    ax.set_xlabel(None)
    ax.set_ylabel('Count', fontsize=35)
    ax.tick_params(axis='both', which='major', labelsize=32)

#plt.savefig('./Figures/Histograms_RawDemog.jpg')
```

In [94]:

```
norm_demog = pg.normality(df_Demographical.loc[(df_Demographical['GroupID']==10), demog_var_continuous])
norm_demog.rename(columns = {'W':'W_test1', 'pval':'pval_test1'}, inplace=True)
norm_demog.round(3)
```

Out[94]:

|  | W\_test1 | pval\_test1 | normal |
| --- | --- | --- | --- |
| Age | 0.941 | 0.252 | True |

In [95]:

```
## Decided not to do any transformation to keep meaningful x axis. However, compute both Spearman and Pearson corr!
#lambdas_demog = Optimal_lambda(df_Demographical.loc[(df_Demographical['GroupID']==10)], ['VS_ys_duration', 'Spontaneous photopsia', 'Nyctalopia'])
#print(lambdas_demog.round(3))
#df_demog_PT = PowerTransf(df_Demographical.loc[(df_Demographical['GroupID']==10), ['VS_ys_duration', 'Spontaneous photopsia', 'Nyctalopia']], lambdas_demog)
#norm_dem_PT = pg.normality(df_demog_PT)
#norm_dem_PT.rename(columns = {'W':'W_test1', 'pval':'pval_test1'}, inplace=True)
#norm_dem_PT.round(3)
```

# Psychophysics (df\_Visit1\_final) vs. Symptoms (df\_Symptoms)¶

## Test performances vs. continuous symptoms (Spearman correlations)¶

In [96]:

```
df_Symptoms_continuous = df_Symptoms.loc[:, symptom_var_continuous]
df_Symptoms_continuous.head()
```

Out[96]:

|  | VS\_ys\_duration | Severity of VS (VAS) | Palinopsia | BlueField\_EntopticPhenomena | Spontaneous photopsia | Floaters | Flashes in darkness | Photophobia | Nyctalopia | Density | Speed | SurfaceDependence | Distraction | TimeCourse | Size | Outdoor\_SunnyDay | Outdoor\_CloudyDay | Outdoor\_RainyDay | Indoor | FluorescentLighting | Outdoor\_NightTime |
| --- | --- | --- | --- | --- | --- | --- | --- | --- | --- | --- | --- | --- | --- | --- | --- | --- | --- | --- | --- | --- | --- |
| 0 | 3.0 | 7.5 | 0.0 | 6.5 | 4.5 | 7.5 | 6.5 | 4.0 | 0.0 | 4.23 | 2.87 | 2.57 | 3.63 | 4.00 | 5.00 | 3.0 | 3.0 | 2.0 | 5.0 | NaN | NaN |
| 1 | 1.0 | 4.0 | 0.0 | 7.0 | 2.0 | 7.0 | 5.0 | 7.0 | 3.0 | 2.27 | 4.00 | 2.43 | 2.33 | 2.17 | 1.97 | 6.0 | 5.0 | 3.0 | 4.0 | NaN | NaN |
| 2 | 2.0 | 6.0 | 1.0 | 6.0 | 0.0 | 9.0 | 4.0 | 5.0 | 4.0 | 3.03 | 3.10 | 3.30 | 2.53 | 4.00 | 2.00 | 3.0 | 5.0 | 6.0 | 4.0 | 2.0 | 1.0 |
| 3 | 2.0 | 4.0 | 1.5 | 7.0 | 1.5 | 6.0 | 2.0 | 6.5 | 3.0 | 2.53 | 2.87 | 2.83 | 2.30 | 1.70 | 2.90 | 4.0 | 1.0 | 7.0 | 5.0 | 3.0 | 4.0 |
| 4 | 2.0 | 6.0 | 0.0 | 0.0 | 3.0 | 4.5 | 4.0 | 4.0 | 3.5 | 2.60 | 2.93 | 2.37 | 2.30 | 1.70 | 2.90 | 3.0 | 4.0 | 7.0 | 2.0 | 2.0 | 1.0 |

In [97]:

```
## Spearman's corr

df_results = pd.DataFrame(columns=variables, index=symptom_var_continuous)

for idxP, varP in enumerate(variables):
    for idxS, varS in enumerate(symptom_var_continuous):
        corr_results = pg.corr(df_Visit1_final.loc[(df_Visit1_final['GroupID']==10), varP], df_Symptoms_continuous.loc[:, varS], method='spearman').round(3)
        r_results = corr_results.loc[:,'r'][0].round(2).copy().astype(str) + ', ' + corr_results.loc[:,'p-val'][0].round(2).copy().astype(str)
        df_results.iloc[idxS, idxP] = r_results

#df_results
```

In [98]:

```
## recompute VS_ys_duration corr correcting for Age (partial corr)
df_results.loc['VS_ys_duration_partial', :] = None

for idxP, varP in enumerate(variables):
    # Merge the DataFrames based on SubjectID
    merged_df = pd.merge(df_Visit1_final.loc[(df_Visit1_final['GroupID']==10), ['SubjectID',varP]], df_Symptoms.loc[:, ['SubjectID','VS_ys_duration']], on='SubjectID').merge(df_Demographical.loc[(df_Demographical['GroupID']==10), ['SubjectID','Age']], on='SubjectID')

    # Compute partial correlation controlling for 'age'
    partial_corr_result = pg.partial_corr(merged_df, varP, 'VS_ys_duration', 'Age', method='spearman').round(3)
    r_results = partial_corr_result.loc[:,'r'][0].round(2).copy().astype(str) + ', ' + partial_corr_result.loc[:,'p-val'][0].round(2).copy().astype(str)
    df_results.loc['VS_ys_duration_partial', varP] = r_results

df_results
```

Out[98]:

|  | VA | Con | CMot | VBM | HoneyW | Stroop | Posner |
| --- | --- | --- | --- | --- | --- | --- | --- |
| VS\_ys\_duration | 0.1, 0.68 | -0.4, 0.09 | -0.4, 0.09 | -0.54, 0.03 | -0.19, 0.48 | 0.05, 0.83 | -0.09, 0.71 |
| Severity of VS (VAS) | -0.18, 0.45 | -0.7, 0.0 | -0.55, 0.01 | -0.19, 0.48 | -0.36, 0.17 | 0.03, 0.91 | -0.09, 0.72 |
| Palinopsia | -0.22, 0.36 | -0.15, 0.54 | -0.56, 0.01 | -0.43, 0.09 | 0.2, 0.46 | 0.32, 0.17 | -0.25, 0.29 |
| BlueField\_EntopticPhenomena | -0.02, 0.93 | -0.24, 0.33 | -0.06, 0.82 | 0.02, 0.96 | 0.34, 0.19 | -0.32, 0.16 | -0.14, 0.56 |
| Spontaneous photopsia | -0.46, 0.05 | -0.56, 0.01 | -0.45, 0.05 | -0.32, 0.23 | -0.1, 0.71 | -0.05, 0.83 | -0.18, 0.45 |
| Floaters | 0.38, 0.1 | -0.33, 0.17 | -0.15, 0.53 | 0.17, 0.53 | 0.23, 0.4 | -0.29, 0.21 | 0.08, 0.74 |
| Flashes in darkness | -0.07, 0.77 | -0.48, 0.04 | -0.25, 0.3 | -0.08, 0.76 | -0.14, 0.6 | -0.07, 0.78 | -0.06, 0.79 |
| Photophobia | -0.06, 0.8 | -0.39, 0.1 | -0.13, 0.6 | -0.25, 0.36 | 0.08, 0.78 | -0.04, 0.88 | 0.05, 0.85 |
| Nyctalopia | -0.2, 0.43 | -0.23, 0.36 | 0.19, 0.44 | 0.18, 0.52 | -0.01, 0.97 | -0.16, 0.52 | -0.0, 0.98 |
| Density | -0.01, 0.97 | -0.74, 0.0 | -0.62, 0.0 | -0.18, 0.51 | -0.36, 0.17 | 0.14, 0.55 | 0.03, 0.89 |
| Speed | 0.2, 0.41 | -0.16, 0.51 | 0.05, 0.85 | -0.1, 0.72 | -0.28, 0.3 | 0.06, 0.8 | 0.42, 0.06 |
| SurfaceDependence | 0.06, 0.8 | -0.5, 0.03 | -0.6, 0.01 | -0.04, 0.88 | -0.14, 0.6 | 0.36, 0.12 | 0.06, 0.81 |
| Distraction | 0.01, 0.97 | -0.66, 0.0 | -0.7, 0.0 | -0.38, 0.15 | -0.49, 0.05 | 0.21, 0.37 | 0.08, 0.75 |
| TimeCourse | 0.33, 0.17 | -0.45, 0.05 | -0.73, 0.0 | -0.01, 0.97 | -0.15, 0.58 | 0.21, 0.36 | 0.11, 0.64 |
| Size | -0.22, 0.38 | -0.33, 0.17 | -0.29, 0.23 | -0.08, 0.78 | -0.3, 0.26 | 0.09, 0.7 | -0.0, 0.99 |
| Outdoor\_SunnyDay | -0.19, 0.46 | -0.0, 1.0 | -0.09, 0.74 | -0.37, 0.19 | 0.31, 0.28 | 0.21, 0.41 | -0.23, 0.36 |
| Outdoor\_CloudyDay | -0.14, 0.59 | -0.14, 0.6 | -0.1, 0.7 | -0.41, 0.15 | -0.08, 0.79 | 0.02, 0.93 | 0.35, 0.15 |
| Outdoor\_RainyDay | -0.25, 0.34 | 0.15, 0.56 | 0.28, 0.28 | 0.22, 0.45 | 0.1, 0.73 | 0.16, 0.52 | 0.14, 0.59 |
| Indoor | 0.49, 0.04 | -0.12, 0.64 | -0.01, 0.98 | 0.55, 0.04 | -0.16, 0.59 | 0.21, 0.41 | 0.06, 0.8 |
| FluorescentLighting | 0.27, 0.34 | -0.25, 0.38 | -0.12, 0.68 | 0.14, 0.65 | -0.12, 0.7 | 0.14, 0.6 | -0.34, 0.2 |
| Outdoor\_NightTime | 0.26, 0.35 | 0.73, 0.0 | 0.16, 0.57 | 0.48, 0.1 | 0.34, 0.28 | 0.08, 0.78 | 0.21, 0.44 |
| VS\_ys\_duration\_partial | 0.14, 0.56 | -0.55, 0.02 | -0.28, 0.26 | -0.35, 0.2 | -0.18, 0.53 | -0.06, 0.81 | -0.22, 0.36 |

In [99]:

```
pg.corr(df_Visit1_final.loc[(df_Visit1_final['GroupID']==10), 'Con'], df_Symptoms_continuous.loc[:, 'Distraction'], method='spearman')
```

Out[99]:

|  | n | r | CI95% | p-val | power |
| --- | --- | --- | --- | --- | --- |
| spearman | 19 | -0.6611 | [-0.86, -0.3] | 0.002056 | 0.90076 |

In [100]:

```
## Plot1: corr variables vs. VS_ys_duration, Severity of VS (VAS)

fig, axs = plt.subplots(2, 7, figsize=(20, 6), sharey=False, sharex=False)

for i, varS in enumerate(symptom_var_continuous[0:2]):
    for j, varP in enumerate(variables):
        ax = axs[i,j]
        ax.scatter(df_Symptoms_continuous.loc[:, varS], df_Visit1_final.loc[df_Visit1_final['GroupID'] == 10, varP],  color='orangered', alpha=0.6)
        #ax.set_xlabel(varP, fontsize=13)
        #ax.set_ylabel(varS, fontsize=13)
        ax.tick_params(axis='both', which='major', labelsize=15)

#plt.tight_layout()       
#plt.show()

plt.savefig('./Figures/8.Scatterplots_VS_Visual.jpg', dpi=300)
```

In [101]:

```
## Plot2: corr variables vs. VSS symptoms (7 variables)

fig, axs = plt.subplots(7, 7, figsize=(20, 21), sharey=False, sharex=False)

for i, varS in enumerate(symptom_var_continuous[2:9]):
    for j, varP in enumerate(variables):
        ax = axs[i, j]
        ax.scatter(df_Symptoms_continuous.loc[:, varS], df_Visit1_final.loc[df_Visit1_final['GroupID'] == 10, varP],  color='orangered', alpha=0.6)
        #ax.set_xlabel(varP, fontsize=13)
        #ax.set_ylabel(varS, fontsize=13)
        ax.tick_params(axis='both', which='major', labelsize=15)

#plt.tight_layout()       
#plt.show()

plt.savefig('./Figures/9.Scatterplots_VSSsymptoms_Visual.jpg', dpi=300)
```

In [102]:

```
## Plot3: corr variables vs. VSS characteristics (6 variables)

fig, axs = plt.subplots(6, 7, figsize=(20, 18), sharey=False, sharex=False)

for i, varS in enumerate(symptom_var_continuous[9:15]):
    for j, varP in enumerate(variables):
        ax = axs[i, j]
        ax.scatter(df_Symptoms_continuous.loc[:, varS], df_Visit1_final.loc[df_Visit1_final['GroupID'] == 10, varP],  color='orangered', alpha=0.6)
        #ax.set_xlabel(varP, fontsize=13)
        #ax.set_ylabel(varS, fontsize=13)
        ax.tick_params(axis='both', which='major', labelsize=15)

#plt.tight_layout()       
#plt.show()

plt.savefig('./Figures/10.Scatterplots_VSScharacteristics_Visual.jpg', dpi=300)
```

In [103]:

```
## Plot4: corr variables vs. VSS severity (6 variables)

fig, axs = plt.subplots(6, 7, figsize=(20, 18), sharey=False, sharex=False)

for i, varS in enumerate(symptom_var_continuous[15: ]):
    for j, varP in enumerate(variables):
        ax = axs[i, j]
        ax.scatter(df_Symptoms_continuous.loc[:, varS], df_Visit1_final.loc[df_Visit1_final['GroupID'] == 10, varP],  color='orangered', alpha=0.6)
        #ax.set_xlabel(varP, fontsize=13)
        #ax.set_ylabel(varS, fontsize=13)
        ax.tick_params(axis='both', which='major', labelsize=15)

#plt.tight_layout()       
#plt.show()

plt.savefig('./Figures/11.Scatterplots_VSSseverity_Visual.jpg', dpi=300)
```

## Test performance vs. binary symptoms (Welch's t-Test) - Comorbidities¶

In [104]:

```
def tTest_Welch_symptomTasks(feature, df_1, df_2): # Welch's t-test
    # Save: T, dof, p-val, cohen-d
    tt_results = pg.ttest(df_1[feature], df_2[feature], correction=True).round(3) 
    final_results = tt_results.loc[:,['T','dof','p-val','cohen-d']].round(2).copy()
    final_results = final_results.rename(columns={'T':'Welch T-test t'})
    final_results.insert(loc = 0, column = 'Variable:', value = feature + " vs " + var_sym)
    return final_results
```

In [105]:

```
df_Symptoms_binary = df_Symptoms.loc[:, symptom_var_binary]
df_Symptoms_binary.head()
```

Out[105]:

|  | Tinnitus | Migraine | Migraine with aura |
| --- | --- | --- | --- |
| 0 | 1 | 0 | NaN |
| 1 | 0 | 1 | 1.0 |
| 2 | 1 | 0 | NaN |
| 3 | 0 | 0 | NaN |
| 4 | 0 | 1 | 0.0 |

In [106]:

```
df_results = pd.DataFrame()

for var in variables:
    for var_sym in symptom_var_binary:
        
        df_tmp = pd.merge(df_Visit1_final.loc[(df_Visit1_final['GroupID']==10), var], df_Symptoms_binary.loc[:, var_sym], left_index=True, right_index=True)
        
        result = tTest_Welch_symptomTasks(var, df_tmp.loc[(df_tmp[var_sym]==0)], df_tmp.loc[(df_tmp[var_sym]==1)])    
        df_results = pd.concat([df_results, result], axis = 0)
    
df_results
```

```
C:\Users\garobbio\Anaconda3\lib\site-packages\pingouin\parametric.py:257: RuntimeWarning: Degrees of freedom <= 0 for slice
  vx, vy = x.var(ddof=1), y.var(ddof=1)
C:\Users\garobbio\Anaconda3\lib\site-packages\numpy\core\_methods.py:256: RuntimeWarning: invalid value encountered in double_scalars
  ret = ret.dtype.type(ret / rcount)
C:\Users\garobbio\Anaconda3\lib\site-packages\numpy\core\fromnumeric.py:3757: RuntimeWarning: Degrees of freedom <= 0 for slice
  return _methods._var(a, axis=axis, dtype=dtype, out=out, ddof=ddof,
C:\Users\garobbio\Anaconda3\lib\site-packages\pingouin\effsize.py:803: RuntimeWarning: Degrees of freedom <= 0 for slice
  poolsd = np.sqrt(((nx - 1) * x.var(ddof=1) + (ny - 1) * y.var(ddof=1)) / dof)
```

Out[106]:

|  | Variable: | Welch T-test t | dof | p-val | cohen-d |
| --- | --- | --- | --- | --- | --- |
| T-test | VA vs Tinnitus | -0.5 | 3.50 | 0.65 | 0.39 |
| T-test | VA vs Migraine | 0.15 | 14.91 | 0.88 | 0.07 |
| T-test | VA vs Migraine with aura | -0.5 | 1.08 | 0.7 | 0.7 |
| T-test | Con vs Tinnitus | 2.07 | 12.20 | 0.06 | 0.75 |
| T-test | Con vs Migraine | 0.33 | 16.99 | 0.75 | 0.14 |
| T-test | Con vs Migraine with aura | 1.14 | 1.90 | 0.38 | 0.88 |
| T-test | CMot vs Tinnitus | 1.76 | 7.97 | 0.12 | 0.75 |
| T-test | CMot vs Migraine | -0.6 | 15.73 | 0.55 | 0.28 |
| T-test | CMot vs Migraine with aura | 0.51 | 1.66 | 0.67 | 0.43 |
| T-test | VBM vs Tinnitus | 0.38 | 3.15 | 0.73 | 0.23 |
| T-test | VBM vs Migraine | 0.49 | 9.11 | 0.64 | 0.27 |
| T-test | VBM vs Migraine with aura | NaN | 1.00 | NaN | NaN |
| T-test | HoneyW vs Tinnitus | 0.43 | 7.48 | 0.68 | 0.21 |
| T-test | HoneyW vs Migraine | 0.17 | 9.69 | 0.87 | 0.08 |
| T-test | HoneyW vs Migraine with aura | -0.03 | 1.13 | 0.98 | 0.03 |
| T-test | Stroop vs Tinnitus | 1.18 | 17.92 | 0.26 | 0.35 |
| T-test | Stroop vs Migraine | 0.64 | 17.66 | 0.53 | 0.28 |
| T-test | Stroop vs Migraine with aura | 0.96 | 6.91 | 0.37 | 0.41 |
| T-test | Posner vs Tinnitus | -0.12 | 5.55 | 0.91 | 0.06 |
| T-test | Posner vs Migraine | -0.24 | 17.92 | 0.81 | 0.1 |
| T-test | Posner vs Migraine with aura | -1.52 | 4.81 | 0.19 | 0.78 |

In [107]:

```
def binary_comparison(df, features, GroupID): #barplots but can change kind to have boxplots, violinplots....

    df_temp = df.loc[:,  features + [GroupID]].copy()
    df_temp = pd.melt(df_temp, id_vars = GroupID, var_name = 'variable', value_name = 'value') 
    
    g = sb.catplot(data=df_temp, kind="bar", x="variable", y="value", hue=GroupID, palette=['blue','red'], height = 4, aspect = 2, errorbar='se')
    #kind="bar"
    
    g.set_axis_labels("", "Standardized score", fontsize = 18)
    g.despine(left=True)
    g.legend.set_title("")
    #g.set(ylim=(-0.8, 0.8))
    g.set_xticklabels(fontsize = 18)
    g.set_yticklabels(fontsize = 17)
```

In [108]:

```
df_variables_binary = pd.merge(df_Visit1_final.loc[(df_Visit1_final['GroupID']==10), variables], df_Symptoms_binary.loc[:, symptom_var_binary], left_index=True, right_index=True)
df_variables_binary.head()
```

Out[108]:

|  | VA | Con | CMot | VBM | HoneyW | Stroop | Posner | Tinnitus | Migraine | Migraine with aura |
| --- | --- | --- | --- | --- | --- | --- | --- | --- | --- | --- |
| 0 | 0.813577 | -0.975219 | -1.180687 | NaN | -1.140795 | -2.069289 | -0.000000 | 1 | 0 | NaN |
| 1 | 1.094719 | 0.596308 | 0.890983 | 0.683168 | -0.214802 | 0.140614 | 1.276362 | 0 | 1 | 1.0 |
| 2 | 1.586915 | -1.089512 | 0.023606 | 0.683169 | NaN | -0.000000 | 2.119311 | 1 | 0 | NaN |
| 3 | -0.242210 | 0.757120 | 0.821452 | 0.683169 | 0.464628 | 0.349020 | -0.569603 | 0 | 0 | NaN |
| 4 | -1.413431 | -0.101919 | 1.624025 | NaN | -0.929333 | -0.001609 | -0.870642 | 0 | 1 | 0.0 |

In [109]:

```
binary_comparison(df_variables_binary, variables, 'Tinnitus')
plt.savefig('./Figures/12.Barplots_Tinnitus.jpg')
```

In [110]:

```
binary_comparison(df_variables_binary, variables, 'Migraine')
plt.savefig('./Figures/13.Barplots_Migraine.jpg')
```

In [111]:

```
binary_comparison(df_variables_binary, variables, 'Migraine with aura')
plt.savefig('./Figures/14.Barplots_Migraine with aura.jpg')
```

# Psychophysics vs. Demographical data¶

## Test performances controlling for Gender¶

In [112]:

```
## add Gender column from df_Demographical to df_Visit1_final
df_Visit1Gender = pd.merge(df_Visit1_final, df_Demographical.loc[:, ['SubjectID','Gender']], on='SubjectID', how='outer')
```

In [113]:

```
df_Visit1Gender.head()
```

Out[113]:

|  | SubjectID | GroupID | HoneyW | Posner | CMot | VA | Stroop | Con | VBM | Gender |
| --- | --- | --- | --- | --- | --- | --- | --- | --- | --- | --- |
| 0 | 10-101 | 10 | -1.140795 | -0.000000 | -1.180687 | 0.813577 | -2.069289 | -0.975219 | NaN | 1 |
| 1 | 10-102 | 10 | -0.214802 | 1.276362 | 0.890983 | 1.094719 | 0.140614 | 0.596308 | 0.683168 | 0 |
| 2 | 10-103 | 10 | NaN | 2.119311 | 0.023606 | 1.586915 | -0.000000 | -1.089512 | 0.683169 | 1 |
| 3 | 10-104 | 10 | 0.464628 | -0.569603 | 0.821452 | -0.242210 | 0.349020 | 0.757120 | 0.683169 | 0 |
| 4 | 10-105 | 10 | -0.929333 | -0.870642 | 1.624025 | -1.413431 | -0.001609 | -0.101919 | NaN | 1 |

In [114]:

```
#compute ANCOVA

df_results_ANCOVA = pd.DataFrame()

for var in variables:
    ancova_result = pg.anova(dv=var, between=['GroupID', 'Gender'], data=df_Visit1Gender)  
    df_results_ANCOVA = pd.concat([df_results_ANCOVA, ancova_result], axis = 0)

#correct p-values for multiple comparisons with BH (for study eye and other eye separately)
#reject, pvals_corr = pg.multicomp(df_results_ANCOVA.loc[:, 'p-val'], alpha=0.05, method='holm')

# add pvals_corr to df_results
#df_results_ANCOVA['p-BH'] = pvals_corr
df_results_ANCOVA.round(5)
```

Out[114]:

|  | Source | SS | DF | MS | F | p-unc | np2 |
| --- | --- | --- | --- | --- | --- | --- | --- |
| 0 | GroupID | 1.28296 | 1.0 | 1.28296 | 1.87009 | 0.18099 | 0.05521 |
| 1 | Gender | 0.01170 | 1.0 | 0.01170 | 0.01705 | 0.89693 | 0.00053 |
| 2 | GroupID \* Gender | 0.02381 | 1.0 | 0.02381 | 0.03471 | 0.85339 | 0.00108 |
| 3 | Residual | 21.95338 | 32.0 | 0.68604 | NaN | NaN | NaN |
| 0 | GroupID | 0.30720 | 1.0 | 0.30720 | 0.34325 | 0.56220 | 0.01095 |
| 1 | Gender | 1.70166 | 1.0 | 1.70166 | 1.90131 | 0.17780 | 0.05779 |
| 2 | GroupID \* Gender | 0.88881 | 1.0 | 0.88881 | 0.99309 | 0.32670 | 0.03104 |
| 3 | Residual | 27.74482 | 31.0 | 0.89499 | NaN | NaN | NaN |
| 0 | GroupID | 0.54371 | 1.0 | 0.54371 | 0.45943 | 0.50276 | 0.01415 |
| 1 | Gender | 0.44361 | 1.0 | 0.44361 | 0.37485 | 0.54470 | 0.01158 |
| 2 | GroupID \* Gender | 0.21877 | 1.0 | 0.21877 | 0.18486 | 0.67011 | 0.00574 |
| 3 | Residual | 37.87027 | 32.0 | 1.18345 | NaN | NaN | NaN |
| 0 | GroupID | 1.43638 | 1.0 | 1.43638 | 4.01067 | 0.05535 | 0.12933 |
| 1 | Gender | 0.75451 | 1.0 | 0.75451 | 2.10675 | 0.15817 | 0.07238 |
| 2 | GroupID \* Gender | 0.15126 | 1.0 | 0.15126 | 0.42233 | 0.52127 | 0.01540 |
| 3 | Residual | 9.66980 | 27.0 | 0.35814 | NaN | NaN | NaN |
| 0 | GroupID | 1.03339 | 1.0 | 1.03339 | 1.37427 | 0.25062 | 0.04524 |
| 1 | Gender | 1.25783 | 1.0 | 1.25783 | 1.67275 | 0.20610 | 0.05454 |
| 2 | GroupID \* Gender | 0.03535 | 1.0 | 0.03535 | 0.04702 | 0.82986 | 0.00162 |
| 3 | Residual | 21.80675 | 29.0 | 0.75196 | NaN | NaN | NaN |
| 0 | GroupID | 0.22526 | 1.0 | 0.22526 | 0.14839 | 0.70255 | 0.00448 |
| 1 | Gender | 4.44634 | 1.0 | 4.44634 | 2.92914 | 0.09638 | 0.08153 |
| 2 | GroupID \* Gender | 0.04187 | 1.0 | 0.04187 | 0.02758 | 0.86911 | 0.00084 |
| 3 | Residual | 50.09286 | 33.0 | 1.51797 | NaN | NaN | NaN |
| 0 | GroupID | 0.15290 | 1.0 | 0.15290 | 0.16219 | 0.68974 | 0.00489 |
| 1 | Gender | 0.03801 | 1.0 | 0.03801 | 0.04032 | 0.84209 | 0.00122 |
| 2 | GroupID \* Gender | 0.39736 | 1.0 | 0.39736 | 0.42151 | 0.52068 | 0.01261 |
| 3 | Residual | 31.10969 | 33.0 | 0.94272 | NaN | NaN | NaN |

In [115]:

```
df_Visit1Gender.groupby("Gender").mean()
```

Out[115]:

|  | GroupID | HoneyW | Posner | CMot | VA | Stroop | Con | VBM |
| --- | --- | --- | --- | --- | --- | --- | --- | --- |
| Gender |  |  |  |  |  |  |  |  |
| 0 | 60.526316 | 0.365804 | -0.184832 | -0.282106 | 0.108392 | 0.313350 | 0.170215 | 0.138288 |
| 1 | 32.222222 | -0.210606 | -0.067428 | 0.042357 | 0.282310 | -0.487741 | -0.229991 | -0.032406 |

In [116]:

```
def tTest_Welch(feature, df_1, df_2): # Welch's t-test
    # Save: T, dof, p-val, cohen-d
    tt_results = pg.ttest(df_1[feature], df_2[feature], correction=True).round(3) 
    final_results = tt_results.loc[:,['T','dof','p-val','cohen-d']].copy()
    final_results = final_results.rename(columns={'T':'Welch T-test t'})
    final_results.insert(loc = 0, column = 'Variable:', value = feature)
    return final_results
```

In [117]:

```
#compute t-Tests: males vs females in patients

df_results_pat = pd.DataFrame()
df_Visit1GenderPat = df_Visit1Gender.loc[(df_Visit1Gender['GroupID']==10)]

for var in variables:
    result = tTest_Welch(var, df_Visit1GenderPat.loc[(df_Visit1GenderPat['Gender']==0)], df_Visit1GenderPat.loc[(df_Visit1GenderPat['Gender']==1)])    
    df_results_pat = pd.concat([df_results_pat, result], axis = 0)
    
#correct p-values for multiple comparisons with BH (for study eye and other eye separately)
reject, pvals_corr = pg.multicomp(df_results_pat.loc[:,'p-val'], alpha=0.05, method='holm')

# add pvals_corr to df_results
df_results_pat['p-BH'] = pvals_corr.tolist()
df_results_pat
```

Out[117]:

|  | Variable: | Welch T-test t | dof | p-val | cohen-d | p-BH |
| --- | --- | --- | --- | --- | --- | --- |
| T-test | VA | 0.031 | 16.615 | 0.976 | 0.013 | 1.00 |
| T-test | Con | 0.360 | 12.237 | 0.725 | 0.163 | 1.00 |
| T-test | CMot | -0.534 | 7.817 | 0.608 | 0.301 | 1.00 |
| T-test | VBM | 1.963 | 13.956 | 0.070 | 0.811 | 0.42 |
| T-test | HoneyW | 0.952 | 13.564 | 0.358 | 0.406 | 1.00 |
| T-test | Stroop | 2.050 | 13.720 | 0.060 | 0.715 | 0.42 |
| T-test | Posner | -0.492 | 12.109 | 0.632 | 0.232 | 1.00 |

In [118]:

```
#compute t-Tests: males vs females in controls

df_results_con = pd.DataFrame()
df_Visit1GenderCon = df_Visit1Gender.loc[(df_Visit1Gender['GroupID']==90)]

for var in variables:
    result = tTest_Welch(var, df_Visit1GenderCon.loc[(df_Visit1GenderCon['Gender']==0)], df_Visit1GenderCon.loc[(df_Visit1GenderCon['Gender']==1)])    
    df_results_con = pd.concat([df_results_con, result], axis = 0)
    
#correct p-values for multiple comparisons with BH (for study eye and other eye separately)
reject, pvals_corr = pg.multicomp(df_results_con.loc[:,'p-val'], alpha=0.05, method='holm')

# add pvals_corr to df_results
df_results_con['p-BH'] = pvals_corr.tolist()
df_results_con
```

Out[118]:

|  | Variable: | Welch T-test t | dof | p-val | cohen-d | p-BH |
| --- | --- | --- | --- | --- | --- | --- |
| T-test | VA | -0.208 | 6.802 | 0.841 | 0.116 | 1.000 |
| T-test | Con | 2.344 | 13.996 | 0.034 | 0.952 | 0.238 |
| T-test | CMot | -0.158 | 14.937 | 0.876 | 0.062 | 1.000 |
| T-test | VBM | 0.604 | 10.392 | 0.559 | 0.301 | 1.000 |
| T-test | HoneyW | 1.050 | 7.186 | 0.328 | 0.572 | 1.000 |
| T-test | Stroop | 0.976 | 8.637 | 0.356 | 0.490 | 1.000 |
| T-test | Posner | 0.468 | 7.346 | 0.654 | 0.252 | 1.000 |

# Test performance depending on VS color¶

In [119]:

```
# Add ColorID to df_Visit1_final
df_Subgroups = pd.merge(df_Visit1_final, df_Symptoms.loc[:, ['SubjectID','ColorID']], on='SubjectID', how='outer')
```

In [120]:

```
#compute ANOVA: controls vs. b/w vs. others

def run_welch_anova_t_tests(df, columns_to_analyze, group_column):
    # Filter the DataFrame to exclude rows of controls
    #df_filtered = df[df[group_column].isin([1.0, 2.0])]
    df_filtered = df

    # Store significant ANOVA and t-test results
    df_results = pd.DataFrame()

    # Perform Welch's one-way ANOVA for each column
    for column in columns_to_analyze:
        welch_anova_results = pg.welch_anova(data=df_filtered, dv=column, between=group_column)

        final_results = welch_anova_results.loc[:,['ddof1', 'ddof2', 'F', 'p-unc', 'np2']]
        final_results = final_results.rename(columns={'ddof1':'dof1', 'ddof2':'dof2', 'p-unc':'p-value'})
        
        final_results.insert(loc = 0, column = 'variable', value = column)
        
        #Generate all pairs of groups
        test_string_map = {'0.0':'controls','1.0':'b/w','2.0':'others'}
        pairs = [[a, b] for idx, a in enumerate(list(test_string_map.values())) for b in list(test_string_map.values())[idx + 1:]]
    
        #Create empty (nan values) post-hocs dataframe
        post_hocs_cols = [[a[0]+' vs '+a[1]+' dof']+[a[0]+' vs '+a[1]+' T']+[a[0]+' vs '+a[1]+' p-value']+[a[0]+' vs '+a[1]+' p-BHcorrected']+[a[0]+' vs '+a[1]+' effect size'] for a in pairs]
        post_hocs_cols = [element for sublist in post_hocs_cols for element in sublist]
        post_hocs = pd.DataFrame(np.nan, index = [0], columns = post_hocs_cols)
    
        #when there is a need for post-hocs (significant p-value) then the post-hocs cells are filled, otherwise those cells remain empty
        if final_results['p-value'].values[0] < 0.05: 
        #if final_results['p-value'].values[0] < 1: #exploratory.. 
            #Paired t-tests 
            pg_post_hocs = pg.pairwise_tests(data=df_filtered, dv=column, between=group_column, padjust='holm', effsize='cohen')
        
            for i,row in pg_post_hocs.iterrows():
                name_comp = test_string_map[str(row['A'])]+' vs '+test_string_map[str(row['B'])]
                post_hocs[name_comp+' dof'] = row['dof']
                post_hocs[name_comp+' T'] = row['T']
                post_hocs[name_comp+' p-value'] = row['p-unc']
                post_hocs[name_comp+' p-BHcorrected'] = row['p-corr']
                post_hocs[name_comp+' effect size'] = row['cohen']
            
            #we append the ANOVA with the post-hocs tests
            final_results = pd.concat([final_results,post_hocs], axis = 1)

        #gather all results together 
        df_results = pd.concat([df_results, final_results], axis = 0)
    
    return df_results
```

In [121]:

```
## add ColorID=0 to controls

for i, row in df_Subgroups.iterrows():
    if row['GroupID'] == 90:
        df_Subgroups.loc[i, 'ColorID'] = 0
```

In [122]:

```
df_results = run_welch_anova_t_tests(df_Subgroups, variables, 'ColorID')
df_results = df_results.round(2)
df_results
```

Out[122]:

|  | variable | dof1 | dof2 | F | p-value | np2 | controls vs b/w dof | controls vs b/w T | controls vs b/w p-value | controls vs b/w p-BHcorrected | controls vs b/w effect size | controls vs others dof | controls vs others T | controls vs others p-value | controls vs others p-BHcorrected | controls vs others effect size | b/w vs others dof | b/w vs others T | b/w vs others p-value | b/w vs others p-BHcorrected | b/w vs others effect size |
| --- | --- | --- | --- | --- | --- | --- | --- | --- | --- | --- | --- | --- | --- | --- | --- | --- | --- | --- | --- | --- | --- |
| 0 | VA | 2 | 15.62 | 1.14 | 0.35 | 0.07 | NaN | NaN | NaN | NaN | NaN | NaN | NaN | NaN | NaN | NaN | NaN | NaN | NaN | NaN | NaN |
| 0 | Con | 2 | 17.44 | 0.42 | 0.66 | 0.02 | NaN | NaN | NaN | NaN | NaN | NaN | NaN | NaN | NaN | NaN | NaN | NaN | NaN | NaN | NaN |
| 0 | CMot | 2 | 14.97 | 2.85 | 0.09 | 0.14 | NaN | NaN | NaN | NaN | NaN | NaN | NaN | NaN | NaN | NaN | NaN | NaN | NaN | NaN | NaN |
| 0 | VBM | 2 | 12.77 | 1.85 | 0.20 | 0.13 | NaN | NaN | NaN | NaN | NaN | NaN | NaN | NaN | NaN | NaN | NaN | NaN | NaN | NaN | NaN |
| 0 | HoneyW | 2 | 14.61 | 2.19 | 0.15 | 0.13 | NaN | NaN | NaN | NaN | NaN | NaN | NaN | NaN | NaN | NaN | NaN | NaN | NaN | NaN | NaN |
| 0 | Stroop | 2 | 20.51 | 5.28 | 0.01 | 0.19 | 20.42 | -0.87 | 0.4 | 0.4 | -0.32 | 26.75 | 2.22 | 0.04 | 0.07 | 0.8 | 16.89 | 3.2 | 0.01 | 0.02 | 1.41 |
| 0 | Posner | 2 | 16.13 | 0.83 | 0.45 | 0.06 | NaN | NaN | NaN | NaN | NaN | NaN | NaN | NaN | NaN | NaN | NaN | NaN | NaN | NaN | NaN |

In [123]:

```
# Plot

# Replace NaN values of controls with 0
df_Subgroups['ColorID'] = df_Subgroups['ColorID'].fillna(0)
```

In [124]:

```
def color_comparison(df, features): #barplots but can change kind to have boxplots, violinplots....

    df_temp = df.loc[:, features+['ColorID']].copy()
    df_temp = pd.melt(df_temp, id_vars = ['ColorID'], var_name = 'variable', value_name = 'value') 
    
    g = sb.catplot(data=df_temp, kind='strip', x="variable", y="value", hue="ColorID", 
                   palette=['steelblue', 'black', 'violet'], 
                   height = 10, aspect = len(features) * 0.3, errorbar='se', dodge=True)
    #kind="bar"
    
    g.set_axis_labels("", "Standardized score", fontsize = 18)
    g.despine(left=True)
    g.legend.set_title("")
    #g.set(ylim=(-3, 3))
    g.set_xticklabels(fontsize = 25)
    g.set_yticklabels(fontsize = 25)
```

In [125]:

```
color_comparison(df_Subgroups, variables)

#plt.savefig('./Figures/16.Barplot_CtrVsPatColors.jpg') 
plt.savefig('./Figures/16.Strip_CtrVsPatColors2.jpg')
```

In [126]:

```
def stroop_comparison(df, features): #barplots but can change kind to have boxplots, violinplots....

    df_temp = df.loc[:, features+['ColorID']].copy()
    df_temp = pd.melt(df_temp, id_vars = ['ColorID'], var_name = 'variable', value_name = 'value') 
    
    g = sb.catplot(data=df_temp, kind='box', x="variable", y="value", hue="ColorID", 
                   palette=['steelblue', 'black', 'red'], 
                   height = 6, aspect = 1, errorbar='se', dodge=True)
    
    g.set_axis_labels("", "Standardized score", fontsize = 18)
    g.despine(left=True)
    g.legend.set_title("")
    #g.set(ylim=(-0.8, 0.8))
    g.set_xticklabels(fontsize = 18)
    g.set_yticklabels(fontsize = 17)
```

In [127]:

```
stroop_comparison(df_Subgroups, ['Stroop'])

plt.savefig('./Figures/16.Pointsplot_CtrVsPatStroop.jpg')
```
